# Outcome of Central Nervous System Relapses In Childhood Acute Lymphoblastic Leukaemia – Prospective Open Cohort Analyses of the ALLR3 Trial

**DOI:** 10.1371/journal.pone.0108107

**Published:** 2014-10-03

**Authors:** Ashish Narayan Masurekar, Catriona A. Parker, Milensu Shanyinde, Anthony V. Moorman, Jeremy P. Hancock, Rosemary Sutton, Philip J. Ancliff, Mary Morgan, Nicholas J. Goulden, Chris Fraser, Peter M. Hoogerbrugge, Tamas Revesz, Philip J. Darbyshire, Shekhar Krishnan, Sharon B. Love, Vaskar Saha

**Affiliations:** 1 Children’s Cancer Group, Centre for Paediatric, Teenage and Young Adult Cancer, Institute of Cancer, Manchester Academic Health Science Centre, Central Manchester University Hospitals Foundation Trust, The University of Manchester, Manchester, United Kingdom; 2 Centre for Statistics in Medicine, University of Oxford, Oxford, United Kingdom; 3 Leukaemia Research Cytogenetics Group, Northern Institute for Cancer Research, Newcastle University, Newcastle upon Tyne, United Kingdom; 4 Bristol Genetics Laboratory, Southmead Hospital, Bristol, United Kingdom; 5 Children’s Cancer Institute Australia, Lowy Cancer Research Centre, University of New South Wales, Sydney, Australia; 6 Great Ormond Street Hospital, London, United Kingdom; 7 Child Oncology and Haematology Centre, Southampton General Hospital, Southampton, United Kingdom; 8 Queensland Children's Cancer Centre, Brisbane, Australia; 9 Childrens Hospital, Radboud University Nijmegen Medical Centre, Nijmegen, The Netherlands Dutch Childhood Oncology Group, The Hague, The Netherlands; 10 Department of Haematology-Oncology, SA Pathology at Women’s and Children’s Hospital and University of Adelaide, Adelaide, Australia; 11 Department of Haematology, Birmingham Children’s Hospital, Birmingham, United Kingdom; 12 Paediatric Oncology, Tata Translational Cancer Research Centre, Kolkata, India; University of Maryland, United States of America

## Abstract

The outcomes of Central Nervous System (CNS) relapses in children with acute lymphoblastic leukaemia (ALL) treated in the ALL R3 trial, between January 2003 and March 2011 were analysed. Patients were risk stratified, to receive a matched donor allogeneic transplant or fractionated cranial irradiation with continued treatment for two years. A randomisation of Idarubicin with Mitoxantrone closed in December 2007 in favour of Mitoxantrone. The estimated 3-year progression free survival for combined and isolated CNS disease were 40.6% (25·1, 55·6) and 38.0% (26.2, 49.7) respectively. Univariate analysis showed a significantly better survival for age <10 years, progenitor-B cell disease, good-risk cytogenetics and those receiving Mitoxantrone. Adjusting for these variables (age, time to relapse, cytogenetics, treatment drug and gender) a multivariate analysis, showed a poorer outcome for those with combined CNS relapse (HR 2·64, 95% CI 1·32, 5·31, p = 0·006 for OS). ALL R3 showed an improvement in outcome for CNS relapses treated with Mitoxantrone compared to Idarubicin; a potential benefit for matched donor transplant for those with very early and early isolated-CNS relapses.

**Trial Registration:**

Controlled-Trials.com ISRCTN45724312

## Introduction

Acute lymphoblastic leukaemia (ALL) of childhood is a systemic disease with a propensity for post-therapeutic recurrence in the central nervous system (CNS) [1]. Modern chemotherapeutic regimens have significantly decreased the incidence of all types of relapses [2]–[9], primarily by intensification of systemic rather than CNS directed therapy (cranial irradiation and intrathecal therapy) [10], [11]. Nevertheless, 20–40% of relapses continue to occur in the CNS as either isolated (i-CNS) or combined (c-CNS) disease [10]. Management of these relapses remains problematic. In general, early and combined relapses have a poorer prognosis when compared with late and isolated CNS disease [12]–[15]. A combination of 6–12 months systemic therapy with intrathecal methotrexate, followed by cranial irradiation has yielded good results [16]–[21]. Other approaches used have either focused on intensifying CNS directed therapy, such as craniospinal irradiation [20], or intensifying systemic therapy with autologous [18] and allogeneic stem cell transplantation (allo-SCT) [16], [17], [21].

As a significant proportion of patients relapsing off UK frontline trials had CNS disease [10], we designed CNS directed therapy into the ALL R3 relapse trial [22]. Drugs reported to cross the blood brain barrier e.g. dexamethasone, high dose methotrexate and high dose cytarabine were used in systemic chemotherapy along with intrathecal methotrexate. At the end of three blocks of chemotherapy, lasting 13 weeks, patients were either eligible for cranial radiotherapy with continuing chemotherapy or allo-SCT without cranial radiotherapy, based on risk stratification and minimal residual disease (MRD) levels at the end of induction. Patients with late isolated CNS relapses (more than 6 months after stopping therapy) were not eligible for the allo-SCT option, based on previous observations showing that the majority are cured with chemotherapy and CNS directed radiotherapy [14], [15]. All other patients were treated on an uniform strategy for relapsed disease. Very early CNS relapses (within 18 months from first diagnosis) and early (more than 18 months from first diagnosis but within 6 months of stopping therapy) or late c-CNS relapses with high MRD were eligible for allo-SCT. In patients with early or late c-CNS disease where MRD results were not available, based on our previous reported experience [14], allo-SCT was offered to all those in whom relapse had occurred within 24 months of stopping therapy. Relapses occurring 24 months after stopping therapy were not eligible for an allo-SCT. Using these criteria, all early i-CNS relapses were eligible for an allo-SCT. In ALL R3, a randomisation of Mitoxantrone and Idarubicin was performed. The Idarubicin metabolite idarubicinol is thought to cross the blood brain barrier and Mitoxantrone to be active against quiescent cells. Thus the former could be more active in CNS disease and the latter in systemic relapses. We recently reported on the premature closure of the randomisation due to overall benefits of Mitoxantrone in all categories of relapsed ALL [22]. What remained unclear, in the light of the different properties of the drugs, whether this extended to those with CNS relapse? Though the randomisation is now closed, the trial continues to recruit to answer secondary objectives.

We now report on a prospective cohort analysis of the subgroup of patients with CNS relapses treated on the ALL R3 trial. The analysis thus includes patients recruited in both the randomised and non-randomised phases of the trial.

## Materials and Methods

The protocol for this trial and supporting CONSORT checklist are available as supportive information; see Checklist S1 and Protocol S1.

### Patients

Patients aged 1–18 years with a first relapse of ALL, who had not received an allo-SCT in first complete remission (CR1), were eligible for the trial and were recruited from centres of the Children’s Cancer and Leukaemia Group in UK and Ireland; Australian and New Zealand Children’s Haematology and Oncology Group and the Dutch Children’s Oncology Group. This reports includes patients recruited between 1^st^ January 2003 and 31st March 2011, with a median follow up of 46 months. The trial was registered (ISRCTN 45724312 http://www.controlled-trials.com/ISRCTN45724312/ALLR3) on 1^st^ October 2003, after recruitment started. At the time of trial start, it was recommended that trials be registered but not necessarily prior to subject recruitment, hence the short delay in ISRCTN registration. The authors confirm that all ongoing and related trials for this drug/intervention are registered.

### Ethics Statement

Ethics approval obtained from Research Ethics Committee for Wales in the UK, Research Ethics Committee, Our Lady’s Children's Hospital in Ireland, Committee for Human Research (Arnhem – Nimegen Region) in the Netherlands and the Multi-Region Ethics Committee in New Zealand. The following Institutional Review Boards approved the study in Australia: Ethics Committee, The Children’s Hospital at Westmead; Children, Youth and Women’s Health Service Research Ethics Committee, Government of South Australia; Ethics in Human Research Committee, The Royal Children’s Hospital, Melbourne; Royal Children’s Hospital and Health Service District Ethics Committee, Queensland Government; Human Research Ethics Committee C, Southern Health; Hunter New England Human Research Ethics Committee, New South Wales Health; Mater Health Services in Human Research Ethics Committee, Brisbane. Written informed consent was obtained from patients or from parents or legal guardians.

### Definitions

i-CNS disease was defined as more than 5 or more white blood cells per µl of CSF with morphological evidence of blasts or biopsy proven recurrence in the eye or brain with no morphological bone marrow involvement. A c-CNS relapse was defined as presence of CNS disease with ≥5% blasts in a concomitant bone marrow aspirate. An isolated bone marrow (i-BM) relapse was defined as the presence of ≥25% blasts in the bone marrow. Patients were defined as having achieved a second complete remission (CR2) if they had <5% blasts in the marrow; no blasts in the CSF or regression of extramedullary disease including ocular infiltration at the end of induction. Time to relapse was defined as, very early, within 18 months from first diagnosis; early, after 18 months of first diagnosis but within 6 months after end of therapy and late if more than 6 months after end of therapy. Progression Free Survival (PFS) was defined as time from registration date to the first of induction failure (≥5% blasts in the bone marrow or persistence of CSF blasts at the end of induction), relapse, death from any cause or a second malignancy. Overall Survival (OS) was defined as time from registration to date of death from any cause. For risk stratification at first diagnosis, the presence of <25% blasts in the marrow after 8 days (4-drug induction, NCI High Risk) or 15 days (3-drug induction, NCI Standard Risk) were defined as a rapid early response; ≥25% blasts in the same categories was defined as a slow early response.

### MRD

MRD was measured from marrow samples obtained at relapse for those with c-CNS at the end of induction using clonotypic markers for Ig/TCR rearrangements, by quantitative PCR as previously described [22]. MRD^lo^ was defined as fewer than 10^−4^ cells with two sensitive markers (quantitative range 10^−4^) and MRD^hi^ as at least one marker of 10^−4^ cells or more at the end of induction. End of induction MRD analysis was not possible in patients with i-CNS [23].

### Cytogenetics

Cytogenetic analysis was done in local laboratories and reviewed centrally by the Leukaemia Research Cytogenetic Group at the Northern Institute for Cancer Research, University of Newcastle. Diagnostic and relapse karyotypes were used to classify patients with progenitor B-cell into good, intermediate and poor risk cytogenetic subgroups based on the presence of specific chromosomal abnormalities and characterised by their relapse risk [24]. T-cell patients were characterised separately.

### Treatment

The therapeutic protocol has been published [22] and available at http://www.medicine.manchester.ac.uk/images/cancer/PAYAC/documents/pdf/R3Protocolv4_31Aug2007.pdf. As per the trial design, all very early relapses and early and late c-CNS relapses with MRD levels ≥10^−4^ (MRD^hi^) at the end of induction were eligible for allo-SCT as were early i-CNS relapses where an end of induction MRD assessment is uninformative. Where MRD was unavailable for technical reasons in patients with early or late c-CNS relapse, allo-SCT was recommended if disease recurrence was within 24 months of stopping therapy. Early and late c-CNS relapses with MRD <10^−4^ (MRD^lo^) and late isolated CNS relapses were not eligible for transplantation, received 24 Gy as fractionated cranial irradiation prior to week 14 and continued on chemotherapy for a total of 104 weeks with no further intrathecal methotrexate. Transplanted patients were conditioned with cyclophosphamide and total body irradiation, There was a day 1 randomisation of Mitoxantrone versus Idarubicin. As previously reported, this randomisation closed in December 2007 after 212 patients had been recruited and all subsequent patients registered on the trial have received Mitoxantrone. Thus the patients analysed represent those in the randomised cohort as well as those enrolled post randomisation.

### Statistical Analyses

This is a cohort analysis of those with CNS relapse from the ALL R3 trial. PFS and OS, time was calculated in months and the primary hypothesis generating analysis was performed using the Kaplan Meier method and log rank test. Cox regression analysis was done to assess the effect of type of CNS relapse after adjusting for pre-specified prognostic covariates; age group (<10, ≥10 years), immunophenotype (B-cell, T-cell), time to relapse (very early, early and late), cytogenetics (poor, intermediate, good, failed/unknown and T-cell), treatment drug (Idarubicin, Mitoxantrone) and sex. The Cox model for PFS was used to separately assess interactions of the treatment drug with the following variables; time to relapse, relapse site, immunophenotype and actual SCT. Interactions with the type of CNS relapse were assessed using the following covariates; time to relapse, immunophenotype and CSF blast count (≤50, 51–99, ≥100). For MRD at day 35, only intermediate risk patients were included. The proportional hazards assumption was assessed using Schoenfeld residuals and was found to be acceptable in all cases. This analysis was performed using STATA version 11.

The cumulative distribution function was used to describe potential differences between age groups by treatment related events, defined as post induction failure and toxic-related deaths. For censored patients, time from registration to date last known alive or date of death if the patient did not have a disease related event was used. For toxic deaths, time to event was defined as time from date of registration to date of death if cause of death was due to toxicity.

## Results

**Figure 1:**
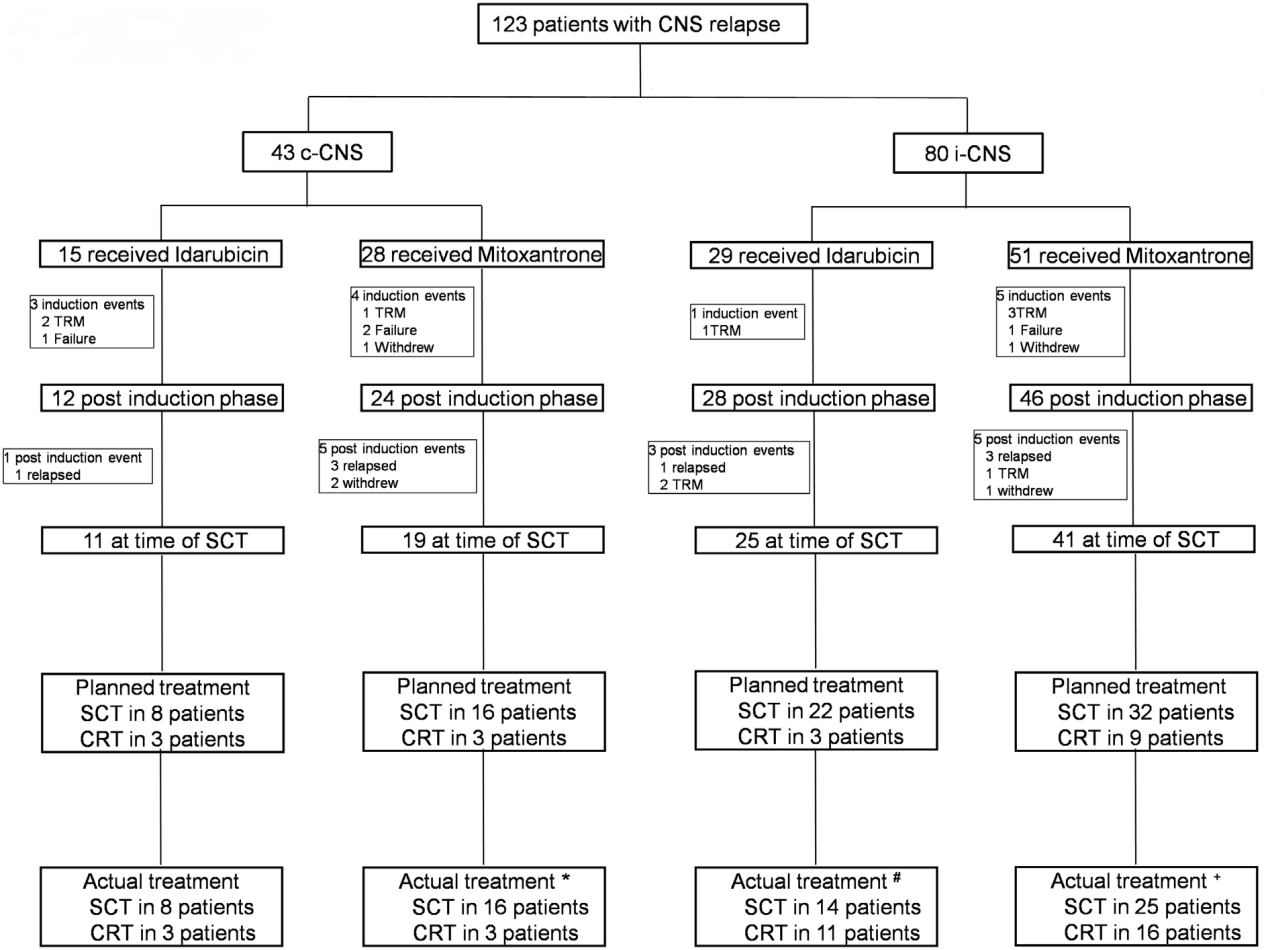
Consort diagram. Showing the details of patients with central nervous system (CNS) relapse. c-CNS = combined bone marrow and CNS relapse; i-CNS = isolated CNS relapse; TRM = treatment related mortality; SCT = allogenic bone marrow transplant; CRT = chemo-radiotherapy. *1 patient planned for SCT received CRT and vice-versa; #9 patients planned for SCT received CRT; +9 patients planned for SCT received CRT and 2 patients planned for CRT got SCT. No patients with i-CNS had detectable MRD in the marrow at the end of induction. Twenty four patients with c-CNS has detectable marrow disease at week 5; 13 MRD positive and 9 MRD negative.

**Figure 2:**
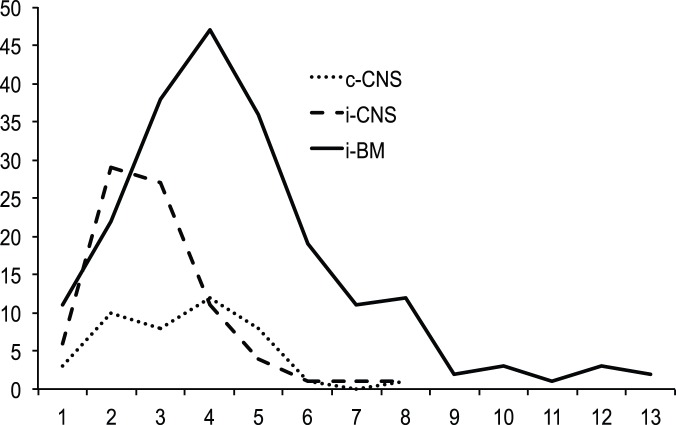
Temporal pattern of relapses. Isolated (iCNS) and combined (c-CNS) relapses occur significantly earlier than those with isolated bone marrow (iBM) relapses (Log rank p = <0.001). Figures in parenthesis show mean duration of remission in months for each group with ± standard deviation.

Three hundred and thirty children were recruited to the trial between January 1, 2003 and March 31, 2011. Of these, 207 had i-BM; 43 c-CNS and 80 i-CNS relapses (Table 1 and Figure 1). Comparing the two groups, CNS recurrence, in particular i-CNS was significantly higher for males (p = <0·001). Of the 44 T-cell relapses, 26 (59%) had recurrence in the CNS compared to 97 (34%) of 286 progenitor B-cell relapses (p = 0·001). The temporal patterns of CNS relapses were similar and peaked before i-BM disease (p = <0.001) (Figure 2). The median follow up was 46·1 (39·8–52·7) months for all patients (95% CI, reverse Kaplan-Meier method). The estimated 3-year PFS and OS of the whole group (n = 330) were 51·2% (45·3, 56·9) and 58·3% (52·3, 63·9) respectively. Further analyses with respect to this report are restricted to the outcome of those with a CNS relapse (n = 123). The estimated 3-year PFS for those with c-CNS and i-CNS were 40·6% (25·1, 55·6) and 38·0% (26·2, 49·7) p = 0.876 respectively. The estimated 3-year OS for those with c-CNS and i-CNS were 40·9% (24·9, 56·3), 50·8% (37·5, 62·6) p = 0·303 respectively.

**Table 1:** Characteristics of those with isolated (i-CNS) or combined (c-CNS) CNS relapses compared with those with an isolated bone marrow relapse (i-BM).

	c-CNS	i-CNS	i-BM
N	43	80	207
*Age at relapse in years*			
median (25^th^−75^th^)	8·7 (6·8–13·7)	8·4 (6·1–11·5)	9·75 (7–13·4)
Range	2·5–18·8	2·0–17·9	1·1–18·8
*Gender*			
Male:Female	27∶16	61∶19	104∶103
Ratio	1·7∶1	3·2∶1	1∶1
*Time to Relapse in months*			
median (25^th^−75^th^)	37 (23–47)	27 (21–34·3)	44 (33–61)
Range	6–92	5–90	5–155
Late	20 (47%)	12 (15%)	142 (69%)
Early	16 (37%)	55 (69%)	48 (23%)
Very Early	7 (16%)	13 (16%)	17 (8%)
*Risk category (%)*			
Standard	-	12 (15%)	-
Intermediate	20 (47%)	68 (85%)	135 (65%)
High	23 (53%)	-	72 (35%)
*Immunophenotype*			
B:T	36∶7	61∶19	189∶18
Ratio	5∶1	3∶1	11∶1
*CSF blast count*			
median (range)	97 (14–188)	97 (27–392)	NA
*Bone Marrow Blast %*			
median (range)	83·5 (48·5–95)	NA	90 (78–95)
*Cytogenetics (%)*			
progenitor B			
Good	15 (35)	28 (35)	77 (37)
Intermediate	11 (25)	18 (23)	63 (30)
Poor	5 (12)	6 (8)	34 (16)
Unknown	5 (12)	9 (11)	15 (7)
T-cell	7 (16)	19 (24)	18 (9)

Relapses within 18 months of first diagnosis were termed as Very Early; after 18 months but within 6 months of stopping therapy as Early and after 6 months of stopping therapy as Late. B = progenitor B cell; T = T-cell; CSF = cerebrospinal fluid. CSF blast count is presented as cells/µl of CSF. Risk stratification and cytogenetic classification have previously been described.

A schema of the risk stratified treatment approach, based on duration of first remission, immunophenotype and site of relapse, for those with CNS disease is shown in Table S1 in File S1, along with the numbers of patients in each risk group and the allocation for allo-SCT or chemoradiotherapy. Table 2 shows the progression with therapy. 30/43 (70%) and 62/77 (81%) evaluable c-CNS and i-CNS patients completed 3 blocks of intensive therapy to reach the time to transplant (chi-squared test, p = 0.182). Excluding the 13 patients who were withdrawn or had an event during induction, as per the stratification adopted in the trial, 91 patients were eligible for allo-SCT and 19 for chemoradiotherapy.

**Table 2:** Differences in outcome in patients with CNS relapse, allocated either transplantation or chemoradiotherapy according to whether they received Idarubicin or Mitoxantrone.

	c-CNS		i-CNS	
n	43		80	
	Ida	Mito	Ida	Mito
**Induction**				
**n**	**15**	**28**	**29**	**51**
TRM	2	1	1	3
Failure	1	2	0	1
Withdrawn	0	1	0	1
Allocated Treatment*				
**n**	**12**	**24**	**28**	**46**
SCT	9	21	25	36
No SCT	3	3	3	10
**Post Induction**				
**n**	**12**	**24**	**28**	**46**
TRM	0	0	2	2
Relapse	1	3	2	1
Withdrawn	0	2	0	2
**SCT Time point**				
Not Reached	0	0	0	3
Reached	11	19	24	38
**Outcome**				
**SCT Group**	8	16	21	30
*Actual SCT*	8	15	13	21
CR2	3	12	5	16
TRM	3	1	1	2
Relapse	2	2	6	3
Accidental Death	0	0	1	0
*No-SCT*	0	1	8	9
CR2	0	0	0	4
TRM	0	1	0	0
Relapse	0	0	8	5
**Non-SCT Group**	3	3	3	8
*Actual non-SCT*	3	2	3	6
CR2	1	1	3	5
TRM	1	0	0	0
Relapse	1	1	0	1
*SCT*	0	1	0	2
CR2	0	1	0	1
Second Malignancy	0	0	0	1

c-CNS = combined CNS relapse; i-CNS = isolated CNS relapse; TRM = Therapy related mortality; SCT = allogeneic transplantation; CR2 = second remission; Ida = Idarubicin; Mito = Mitoxantrone. *Patients allocated SCT were, all those with very early relapse; early i-CNS relapses and early and late c-CNS relapses with MRD ≥10^−4^ at the end of induction. Where MRD was not evaluable, decision to transplant was based on duration of CR1. All other patients were allocated chemoradiotherapy.

We have previously reported a better outcome for 87 of the 123 patients with CNS relapse who received Mitoxantrone [22]. This continues to the case in this extended cohort with a significant (log rank p = 0.042) increase in disease recurrence in patients who received Idarubicin (Figure S1 in File S1). Mitoxantrone was associated with a significant PFS and OS 3-year survival rates advantage in patients eligible for/or who received an allo-SCT when compared to those patients eligible for/who received Idarubicin (Table 3 and Figure S2 in File S1).

**Table 3:** Endpoint of PFS and OS in patients with CNS relapse grouped by treatment allocation, and according to drug received Idarubicin vs Mitoxantrone.

	n	3 yr PFS (95% CI)	p-value	3 yr OS (95% CI)	p-value
Intended SCT	91				
Idarubicin	34	22·2% (9·9, 37·4)		34·0 (18·7, 49·9)	
Mitoxantrone	57	55·0% (39·4, 68·1)	0·02	63·0% (46·7, 75·5)	0·02
Intended No SCT	19				
Idarubicin	6	66·7% (19·5, 90·4)		66·7% (19·5, 90·4)	
Mitoxantrone	13	61·5% (26·6, 83·7)	0·90	68·6% (21·3, 91·2)	0·81
Actual SCT	60				
Idarubicin	21	36·4% (16·6, 56·6)		35·7% (16·1, 56·0)	
Mitoxantrone	39	72·2% (52·8, 84·7)	0·02	76·0% (55·6, 87·9)	0·01
Actual No SCT	32				
Idarubicin	14	28·6% (8·8, 52·4)		50·0% (22·9, 72·2)	
Mitoxantrone	18	50·4% (22·6, 72·9)	0·20	59·2% (17·6, 85·4)	0·39

Fifty one i-CNS patients, with very early or early relapse and eligible for allo-SCT reached the time point for transplantation (week 13). In the Idarubicin group, 3 of 4 very early and 10 of 17 early relapses were transplanted. In the Mitoxantrone group, all 5 very early and 16 of 25 early relapses were transplanted. The 3-year PFS and OS for 21 i-CNS patients treated with Idarubicin was 21.2 [11.9, 30.5] and 35.6 [24.9, 46.3]; and for 30 patients treated with Mitoxantrone were 60.7 [50.8, 70.6] and 70.8 [61.4, 80.2] respectively (p = 0.027 for PFS and p = 0.032 for OS). Of those eligible for but not transplanted, 13/17 have relapsed. In contrast, there has been only 1 relapse in the 11 patients with late i-CNS relapses not eligible for allo-SCT. For those with c-CNS relapses, the numbers are small, but 15 of 24 patients eligible for allo-SCT are in CR2 (3-year PFS 62.2 [51.4, 73], 3-year OS 65.5 [54.6, 76.4]).

Table 4 shows the 3-year PFS, OS and the univariate unadjusted hazard ratio (HR) for PFS and OS for various factors contributing to outcome. From the analysed risk factors at original diagnosis, presenting age, but not white cell count or early response to therapy were predictive of outcome at CNS relapse. At relapse, late recurrence, age less than 10 years, progenitor B-cell disease, standard and intermediate risk groups, good risk cytogenetics and those who received Mitoxantrone had a better prognosis. As the randomisation was terminated early there was an imbalance in patients receiving Mitoxantrone. Adjusting for the variables (age at relapse, time to relapse, cytogenetics, treatment drug and gender) in a multivariate analysis, showed a poorer outcome for those with c-CNS relapse (HR 1·82, 95% CI 0·98, 3·40, p = 0·059 c-index = 0.70 95% CI 0.64,0.75 for PFS and HR 2·64, 95% CI 1·32, 5·31, p = 0·006 c-index = 0.72 95% CI 0.66,0.78 for OS).

**Table 4:** Outcome of those with a CNS relapses in ALL R3 by risk parameters defined at first diagnosis, and at relapse.

				Univariate Analysis			
				PFS		OS	
	N	%PFS	%OS	Hazard ratio	p-value	Hazard ratio	p-value
**At Original Diagnosis**							
*Presenting WC*							
WC <50×10^9^/l	73	53·8 (40·2, 65·6)	46·6 (34·1,58·1)	1		1	
WC ≥50×10^9^/l	35	42·9 (25·0, 59·6)	31·6 (16·0–48·3)	1·37 (0·81, 2·32)	**0·246**	1·19 (0·67, 2·13)	**0·549**
Unknown	15	-	-				
*Age at Diagnosis*							
<10 years	97	54·8 (43·1, 65·1)	46·0 (34·8, 56·4)	1		1	
≥10 years	26	15·5 (2·9, 37·3)	9·9 (0·9,31·9)	3·03 (1·79, 5·12)	**<0·001**	2·76 (1·57, 4·87)	**<0·001**
*Early Response*							
Rapid Early Response	67	47·5 (33·7, 60·0)	39·6 (27·1, 51·8)	1		1	
Slow Early Response	29	42·1 (23·5, 59·7)	35·8 (18·6, 53·4)	1·40 (0·79, 2·46)	**0·245**	1·41 (0·77, 2·58)	**0·261**
Unknown	27	-	-				
**At Relapse**							
*Age at relapse*							
<10 years	78	48·2 (36·6, 59·8)	55·4 (42·1, 66·7)	1		1	
≥10 years	45	22·9 (10·7, 37·9)	33·1 (17·9, 49·0)	2·11 (1·31, 3·40)	**0·002**	1·94 (1·15, 3·24)	**0·012**
*Gender*							
Female	35	44·5 (26·1, 61·4)	42·3 (23·9, 59·5)	1		1	
Male	88	36·5 (25·6, 47·6)	49·1 (36·8, 60·3)	1·30 (0·75, 2·50)	**0·354**	0·98 (0·56, 1·72)	**0·94**
*Immunophenotype*							
B-cell	97	41·9 (31·1, 52·3)	51·7 (39·9, 62·2)	1		1	
T-cell	26	33·9 (16·7, 52·0)	33·3 (14·8, 53·1)	1·95 (1·12, 3·41)	**0·019**	2·09 (1·16, 3·76)	**0·014**
*Time to Relapse*							
Late	32	64·0 (44·2, 78·3)	69·1 (48·7, 82·7)	1		1	
Early	71	27·1 (15·8, 39·8)	36·2 (22·9, 49·7)	2·45 (1·29, 4·70)		2·56 (1·26, 5·17)	
Very Early	20	32·0 (12·3, 53·8)	41·9 (19·5, 63·0)	2·95 (1·34, 6·52)	**0·012**	2·53 (1·07, 5·98)	**0·029**
*Site of Relapse*							
i-CNS	80	38·0 (26·2, 49·7)	50·8 (37·5, 62·6)	1		1	
c-CNS	43	40·6 (25·1, 55·6)	40·9 (24·9, 56·3)	1·04 (0·634, 1·704)	**0·876**	1·32 (0·78, 2·22)	**0·304**
*CNS Blast Count (/µl)*							
<50	42	35·9 (20·0, 52·0)	39·3 (22·4, 55·9)	1		1	
50–99	13	-	16·2 (1·1, 48·1)	1·59 (0·75, 3·36)		1·31 (0·57, 2·97)	
≥100	54	39·4 (25·1, 53·4)	52·8 (36·6, 66·7)	0·93 (0·54, 1·60)	**0·335**	0·69 (0·38, 1·25)	**0·238**
Unknown	14	-	-				
*Risk category*							
Standard	12	64·8 (31·0, 85·2)	80·2 (40·3, 94·8)	1		1	
Intermediate	88	41·4 (30·0, 52·3)	50·5 (38·3, 61·5)	1·87 (0·67, 5·21)		3·29 (0·79, 13·63)	
High	23	15·5 (3·0, 37·2)	11·6 (1·0, 36·9)	3·99 (1·34, 11·94)	**0·008**	7·85 (1·79, 34·43)	**0·002**
*Cytogenetics*							
**progenitor B**							
Good	43	54.8 (38.1, 68.8)	63.5 (45.0, 77.3)	1		1	
Intermediate	29	32.8 (16.0, 50.8)	41.8 (22.7, 59.9)	1.71 (0.89,3.27)		1.96 (0.95, 4.01)	
Poor	11	10.2 (1.0,36.4)	35.1 (8.4, 64.1)	4.38 (1.95,9.82)		3.55 (1.43, 8.82)	
Unknown	14	43.5 (8.3, 75.7)	43.5 (8.3, 75.7)	1.21 (0.45,3.25)		1.95 (0.70, 5.43)	
**T-cell**	26	33.9 (16.7, 52.0)	33.3 (14.8, 53.1)	2.85 (1.46,5.54)	**0.0018**	3.31 (1.60, 6.84)	**0.0126**
*Therapy*							
Idarubicin	44	26·5 (14·4, 40·1)	35·5 (21·7,49·5)	1		1	
Mitoxantrone	79	48·9 (36·0, 60·6)	56·9 (42·9,68·7)	0·65 (0·40,1·04)	0·075	0·57 (0·34, 0·97)	**0·036**

Rapid early response = <25% blasts in the marrow aspirate on day 8/15 of induction; Slow early response = ≥25% blasts in the marrow aspirate on day 8/15 of induction.

**Figure 3:**
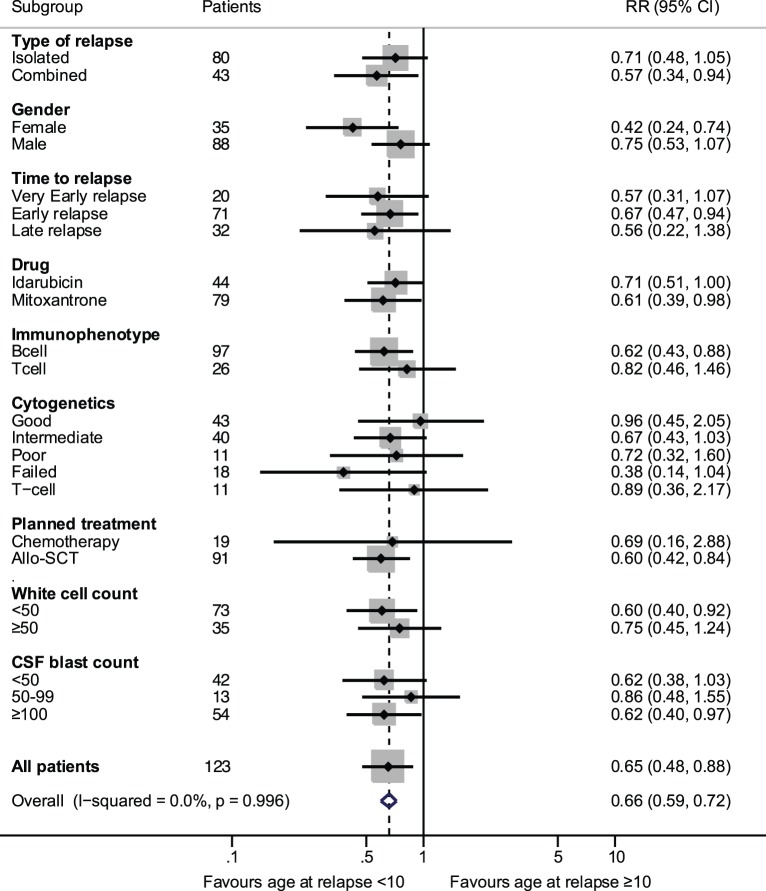
Age at relapse (<10 or ≥10 years) effect on progression-free survival by patient characteristics, from Cox models with interactions. White cell count is the presenting count (×10^9^/L) at first diagnosis. Relative risk ratios indicate that all subgroups show a poorer outcome in those ≥10 years, except good risk cytogenetics.

**Figure 4:**
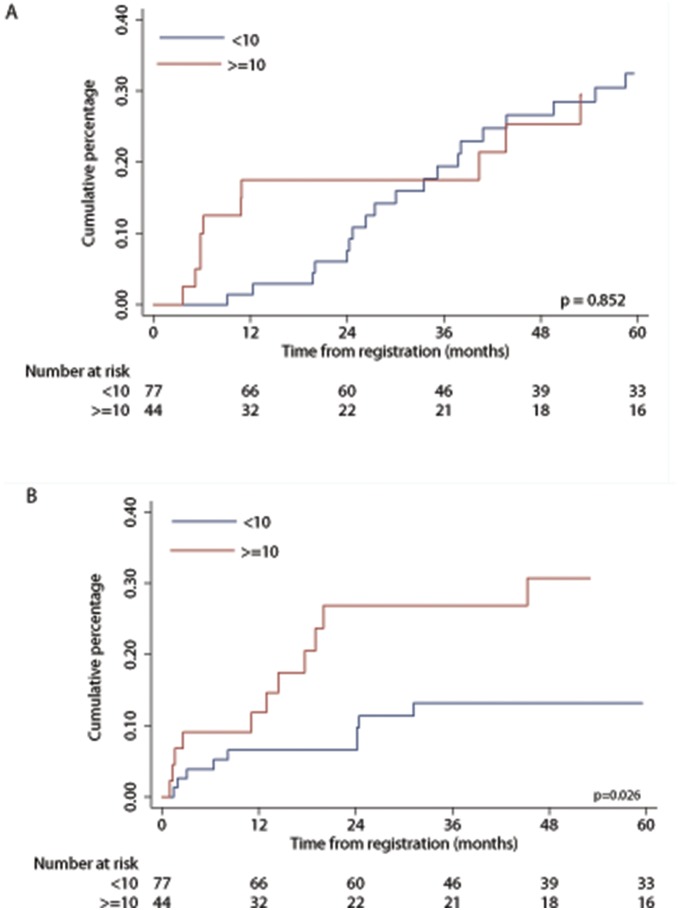
Cumulative percentage of events, in those aged <10 and ≥10 years at first relapse. (A) Therapeutic failure, which includes both induction failures and second relapses. (B) Treatment related deaths.

The late isolated relapses in the i-CNS group have the best outcome (10 of 11 patients are in CR2) and a better salvage rate, so a difference in the adjusted outcome is to be expected. Additionally for PFS multivariate analyses separate assessments of interaction of the treatment drug with time to relapse, relapse site, immunophenotype and actual SCT were not statistically significant. Similarly, the interaction of type of CNS with time to relapse, immunophenotype and CSF blast count were not statistically significant. Exploratory multivariate analysis using pre-specified subgroups showed that age at relapse ≥10 years had an unfavourable outcome (HR 1.91, 95% CI 1.11, 3.28, p = 0.018 for PFS and HR 1.90, 95% CI 1.07, 3.35, p = 0.027 for OS). We tested the effect of potential imbalances of prognostic factors in the two age groups. There were proportionately more c-CNS, males, standard risk, T-cell immunophenotype and fewer good risk cytogenetic patients in the ≥10 year age group (Table 5). Figure 3, shows the risk ratios for PFS, for age at relapse according to the different characteristics. Patients age ≥10 years had a significantly worse outcome in all categories except in those who had good risk cytogenetics. To investigate whether differences seen between the two age groups was due to disease progression and/or side effects of treatment we examined the cumulative percentage of events. As shown in Figure 4 although disease related events are similar between the two groups 24/77 (31%) and 13/44 (30%) for age groups <10 and > = 10 respectively (p = 0.852), older patients relapsed earlier. Overall treatment related mortality was higher in older patients in all phases of therapy 10/77 (13%) and 13/44 (30%) for age groups <10 and > = 10 respectively (p = 0.026).

**Table 5:** Characteristics of patients aged younger than 10, or 10 and older with CNS relapses.

Age at Relapse (years)	<10	≥10
n	78	45
***CNS relapse (%)***		
Isolated	53 (68)	27 (60)
Combined	25 (32)	18 (40)
***Gender***		
Male: Female	51∶27	37∶8
***Time to Relapse (%)***		
median in months (range)	29 (5–92)	33 (6–90)
Late	18 (23)	14 (31)
Early	48 (62)	23 (51)
Very Early	12 (15)	8 (18)
***Risk category***		
Standard	4 (5)	8 (18)
Intermediate	63 (81)	25 (55)
High	11 (14)	12 (27)
***Immunophenotype***		
B:T	66∶12	31∶14
***Cytogenetics (%)***		
progenitor-B cell		
Good	32 (41)	11 (24)
Intermediate	18 (23)	11 (24)
Poor	5 (6)	6 (13)
Unknown	11 (14)	3 (7)
T-cell	12 (15)	14 (22)

Of the 36 second relapses, five (14%) (Four with i-CNS and one with c-CNS) have subsequently achieved CR3 (Table S2 in File S1). Of the 28 patients who relapsed after either receiving a transplant or cranial radiotherapy, there were 19 CNS (17 i-CNS and 2 c-CNS) and 9 i-BM relapses. Thirteen of the 19 CNS relapses were patients who had not been transplanted but had received cranial irradiation. Six of the nine post transplant relapses in the i-CNS group occurred in those with a very early relapse. Thus, salvage after a CNS relapse remains low with a high frequency of disease recurrence in those who received cranial irradiation.

## Discussion

The survival rates of patients treated with mitoxantrone reported here are better than we have previously reported for c-CNS and comparable for those with i-CNS [14]. All relapses described here are of children treated on protocols in existence from 2000 onwards. In the UK these are primarily ALL97 [25] and ALL2003 [26]. This analysis suggests that there has been a change in pattern of CNS relapses in the post 2000 era. While we previously reported i-CNS to c-CNS ratio of 1·1[10], in this report it is 1·9. There also appears to be a temporal change in the pattern of i-CNS relapses with a decrease in very early from 42% to 16% and a proportionate increase in early to 69% from 45%. Thus with time there is an apparent shift towards earlier and c-CNS relapses both of which are associated with an inferior outcome.

The rarity of relapse makes subgroup analyses problematical. However relapsed ALL remains the leading cause of death in children with cancer and our observations may help physicians managing such patients. The key findings of this study are the benefits of, Mitoxantrone for all patients and allo-SCT for early and very early i-CNS relapses. We have previously suggested that the benefit of Mitoxantrone may be related to its toxic effect on the microenvironment [22]. Allogeneic transplantation may have a similar effect, though this study is not designed to answer this question. What is evidenced is that after intensive systemic therapy, consolidation of remission with allo-SCT offers a better curative approach over targeted CNS directed therapy for very early and early CNS relapses. In those with early and very early i-CNS disease, 71% of those allocated allo-SCT but not transplanted (receiving chemoradiotherapy) suffered a second relapse, while recurrence rates were 21% in those transplanted. Late i-CNS relapses occurring off modern intensive chemotherapy regimens continue to be curable with chemo-radiotherapy. There were only a few patients with c-CNS disease not eligible for transplantation. Of the 5 not transplanted, 2 are in CR2. Of the 23 transplanted, 15 are in CR2. Given the poor salvage rate after a second relapse, a more simple approach would be to offer an allo-SCT to all CNS relapses except those with a late i-CNS recurrence.

The poorer outcome of adolescent patients with CNS relapse has been previously reported in a retrospective analysis. Therapy was not uniform and other potential contributing factors to the worse outcome were not examined [9]. In our study, a uniform therapeutic approach, stratified for risk, was taken for all patients, with good results in those aged under 10 but not for older children. The older age group had proportionately more patients with T-cell disease and less with good-risk cytogenetics. The inferior survival of very early and T-cell relapses has been a consistent theme [13]–[15] and in this study, T-cell relapses occurred earlier and in older patients. Good-risk cytogenetics are associated with late relapses [24] and a better prognosis in older patients [27]. Thus both immunophenotype and cytogenetics contributed to the earlier relapse pattern observed in those ≥10 years of age.

The worse outcomes for the ≥10 year age group in this trial were primarily related to toxicity. Paediatric type protocols have considerably improved the outcome of adolescents and young adults newly diagnosed with ALL [28], [29]. This improvement comes at a price of increased toxicity, primarily that of sepsis, osteonecrosis and thrombosis [29], [30]. In ALL R3 we shortened the duration of steroid exposure and used a pegylated derivative to decrease the dose of asparaginase. Neither thrombosis nor osteonecrosis have been of significance though sepsis in older children remains a problem. As older patients tend to relapse early further cytotoxic intensification is not possible. The successor trial for ALLR3, IntReALL 2010 [31] will use the non-toxic targeted drug Epratuzamab in conjunction with post induction therapy patients with late and early CNS relapses. Though Epratuzamab does not cross the blood-brain, our data suggests that systemic therapy is key to maintaining remission in those with a CNS relapse. Thus IntReALL will evaluate if a non-toxic intervention is able to improve the outcome of older patients with relapses in the CNS.

The pathogenesis of CNS disease in ALL remains enigmatic, hindering the development of more targeted less toxic strategies. Recent evidence suggests that leukemic blasts circulate in the blood to the CNS, where they transgress blood-brain and blood-CSF barriers by diapedesis rather than by haemorrhage [32]. The subarachnoid tissue may then provide a chemoprotective microenvironment for lymphoblasts [33] giving rise to recurrence once therapy is discontinued. This supports the use of systemic therapy to prevent and treat CNS recurrence. As illustrated by this report, while the management of relapsed CNS disease using intensive systemic approaches has met with some success, it remains less than satisfactory. We need new agents and novel approaches, based on a better understanding of the disease process. A good proportion of patients eligible for early phase trials are in the older age group. Thus when evaluating new agents, we will also need to allow for age related variations in the therapeutic response, both with regards to efficacy and toxicity, lest we come to inexact conclusions.

## Supporting Information

File S1 **Additional information on survival and further relapse rates by drug (Idarubicin & Mitoxantrone).** Details of risk stratification and treatment allocation and also outcome following second relapse.
(DOCX)

Checklist S1 **CONSORT checklist.**
(DOC)

Protocol S1 **Trial Protocol.**
(PDF)
