# Supplementary material for: Outcome of Central Nervous System Relapses In Childhood Acute Lymphoblastic Leukaemia – Prospective Open Cohort Analyses of the ALLR3 Trial
Source: PLoS One. 2014 Oct 3;9(10):e108107. doi: 10.1371/journal.pone.0108107 (PMC4184796; doi:10.1371/journal.pone.0108107)
Supplement: File S1 — Additional information on survival and further relapse rates by drug (Idarubicin & Mitoxantrone). Details of risk stratification and treatment allocation and also outcome following second relapse. (DOCX) [file pone.0108107.s001.docx]

**Figure S1. Cumulative distribution of second relapses by drug received**

**Table S1. Therapeutic risk stratification of CNS relapses, showing allocated treatment modality and numbers of patients in each group.** SCT = allogeneic stem cell transplantation; Chemo+RT = systemic chemotherapy with CNS directed radiotherapy. * Minimal Residual Disease determined modality of treatment. Those with a MRD ≥ 10^-4^ at the end of induction were allocated allo-SCT. Where MRD could not be assessed, those who had relapsed within 24 months of stopping therapy were also allocated allo-SCT, others received Chemo+RT. One late and 4 early combined pre B patients did not reach the MRD decision point and are not included in this table.

**Figure S2. Kaplan Meier estimation of progression free (A) and overall (B) survival of patients with CNS relapse by drug received.** Figures in parentheses represent 95% confidence intervals

**Table S2.** **Outcome of patients with a second relapse.**
