# Supplementary material for: Outcome of Central Nervous System Relapses In Childhood Acute Lymphoblastic Leukaemia – Prospective Open Cohort Analyses of the ALLR3 Trial
Source: PLoS One. 2014 Oct 3;9(10):e108107. doi: 10.1371/journal.pone.0108107 (PMC4184796; doi:10.1371/journal.pone.0108107)
Supplement: Protocol S1 — Trial Protocol. (PDF) [file pone.0108107.s003.pdf]

Title:

ALLR3

An International Collaborative Trial for Relapsed and Refractory Acute Lymphoblastic Leukaemia (ALL)

AMENDMENT 3 (PROTOCOL VERSION 4) DATED 31 AUG 2007
AMENDMENT 2 (PROTOCOL VERSION 3) DATED 28 APR 2006
AMENDMENT 1 (PROTOCOL VERSION 2) DATED 09 OCT 2003
ORIGINAL APPROVED (PROTOCOL VERSION 1) DATED 21 OCT 2002

Start Date: 17 JAN 2003
MREC Approval Number: 02/9/21

NOT FOR CLINICAL USE

Foreword

ALLR3 is a trial for refractory and relapsed childhood ALL, planned in close collaboration with the I-BFM Study Group. The prime objective of this protocol is to utilise a new comprehensive chemotherapeutic approach. The strategy offers better use of stratification by risk group and therapy directed by the presence of minimal residual disease. The treatment comprises of an intensive induction protocol, prior to continuing therapy or bone marrow transplantation. Pharmacokinetics and biological questions have been built into this protocol. The results of these will not influence treatment at this stage but will be used to plan any required changes and future therapy.

This document is intended to describe an international based collaborative study into relapsed and refractory ALL in children and young adults. The Childhood Leukaemia Working Party does not intend the protocol to be used as an *aide-memoir* or guide to treatment of other patients. Every care was taken in its drafting, but corrections or amendments may be necessary and these will be circulated to regional representatives. Centres entering patients for the first time are advised to contact the Trial Manager.

The trial coordinators have obtained MREC approval for the study but before entering patients into the trial, clinicians must ensure that the trial protocol has received clearance from their local research ethical committee. Clinicians are asked to read the whole protocol before starting treatment and to contact the trial manager if there are any doubts or queries about the protocol.
The trial is currently recruiting in the UK and Ireland and will shortly begin recruitment in The Netherlands, Australia and New Zealand.

Risks of intrathecal therapy

All medical staff involved in the care of patients with leukaemia MUST be aware that the inadvertent administration of vincristine, other than the intravenous route, is invariably FATAL. This protocol has deliberately changed the schedule of vincristine so that it is not administered on the same day as intrathecal methotrexate as per United Kingdom guidelines.

NOT FOR CLINICAL USE

Author
Professor Vaskar Saha, on behalf of the **United Kingdom Childrens Cancer and Leukaemia Group**

Keywords
relapse, acute lymphoblastic leukaemia, minimal residual disease, bone marrow transplant, paediatric.

TrialManagement

Chief Investigator
Professor Vaskar Saha
Paediatric and Adolescent Oncology Unit
Christie Hospital
Manchester M20 4BX
Tel: (+44) 161 446 3094
Fax: (+44) 161 446 3092
Email: *vaskar.saha@manchester.ac.uk*

Co-Investigator
Dr Phil J Darbyshire
Birmingham Children's Hospital
Steelhouse Lane
Birmingham B4 6NH
Tel: (+44) 121 333 9844
Fax: (+44) 121 333 9841
Email: *phil.darbyshire@bch.nhs.uk*

Minimal Residual Disease
Dr Nicholas J Goulden
Great Ormond Street Hospital
London WC1N 3JN
Tel: (+44) 207 405 9200
Fax: (+44) 207 813 8410
Email: *GouldN@gosh.nhs.uk*

Transplant Advisor
Dr Jacqueline M Cornish
Head of Division, Women's and Children's Services
Bristol Royal Hospital for Children
Bristol
Tel: (+44) 117 3428877
Fax: (+44) 117 342 8846
Email: *Jacqueline.Cornish@ubht.nhs.uk*

Associate Investigator
Dr Mary Morgan
Southampton General Hospital
Southampton S016 6YD
Tel: (+44) 238 079 4101
Fax: (+44) 238 079 4962
Email: *morgam1@suht.swest.nhs.uk*

Associate Investigator
Dr Phil J Ancliff
Great Ormond Street Hospital
London WC1N 3JN
Tel: (+44) 207 405 9200 Extn 5361
Fax: (+44) 207 813 8410
Email: *anclip@gosh.nhs.uk*

New Agents and Phase II
Dr Pamela R Kearns
The Institute of Child Health 4th Floor
Whittall Street
Birmingham B4 6NH
Tel: (+44) 121 333 8238
Fax: (+44) 121 333 8701
Email: *p.r.kearns@bham.ac.uk*

Statistics
Sharon Love
Centre for Statistics in Medicine
Wolfson College, Winton Road
Oxford, OX2 6UD
Tel: (+44) 186 528 4414
Fax: (+44) 186 528 4424
Email: *Sharon.love@cancer.org.uk*

PI – Australia and New Zealand
Professor Támás Revesz
Women's and Children's Hospital
72 King William Road
North Adelaide, SA 5006
Australia
Tel: (+61) 18 8161 7327
Fax: (+61) 18 8161 6567
Email: *tom.revesz@cywhs.sa.gov.au*

PI – The Netherlands
Professor Peter Hoogerbrugge
P.O. Box 9101
6500 HB Nijmegen
Netherlands
Tel: (+31) 24 361 6928
Fax: (+31) 24 361 7489
Email: *p.hoogerbrugge@cukz.umcn.nl*

Cytogenetics
Dr Anthony Moorman
Leukaemia Research Cytogenetics Group,
University of Southampton,
MP822, Duthie Building,
Southampton General Hospital,
Tremona Road, Southampton, SO16 6YD
Tel: (+44) 23 8079 8403
Email: *A.V.Moorman@soton.ac.uk*

Trial Manager
Carly Leighton
Paediatric and Adolescent Oncology Unit
Christie Hospital
Manchester M20 4BX
Tel: (+44) 161 446 3093
Fax: (+44) 161 446 3092
Email: *carly.leighton@cancer.org.uk*

NOT FOR CLINICAL USE

SPONSOR INFORMATION PAGE

Title:	ALLR3 : An International Collaborative Trial for Relapsed and Refractory Acute Lymphoblastic Leukaemia (ALL)
Sponsor Contact Details:	University Hospitals of Leicester NHS Trust<br>Trust Headquarters<br>Gwendolen House<br>Gwendolen Road<br>Leicester LE5 4QF
Type of Trial	Phase III
ISCRTN	45724312
EudraCT	2004-000052-16

DATA MONITORING COMMITTEE

Mike Stevens
CLIC Professor of Paediatric Oncology
Institute of Child Life & Health
UBHT Education Centre
Upper Maudlin Street
Bristol BS2 8AE

Moira Stewart
The Western General Hospital
Cancer Research Building
Crewe Road South
Edinburgh EH4 2XR

Dr Rob Edwards
Wolfson Institute for Preventive Medicine
Charterhouse Square
London EC1M 6BQ

NOT FOR CLINICAL USE

SUMMARY OF CHANGES (V4 31ST AUGUST 2007)

1. Change of Sponsor from Barts and the London NHS Trust to University Hospitals of Leicester NHS Trust.
2. Change of Principal Investigators at two participating centres: Bristol and Manchester
3. Change of contact details for Chief Investigator in the UK + Trial Manager in the UK
4. Inclusion of a GP Information Sheet
5. Inclusion of Asparaginase study – detailed sample requirements.
6. Change to SAE report form

NOT FOR CLINICAL USE

TABLE OF CONTENTS

PAGE

FOREWORD 2

TRIALMANAGEMENT 3

SPONSOR INFORMATION PAGE 4

INVESTIGATOR PROTOCOL AMENDMENT AGREEMENT PAGE.......**ERROR! BOOKMARK NOT DEFINED.**

SUMMARY OF CHANGES (V4 28TH JULY 2006) 5

1. TRIAL OVERVIEW AND RATIONALE 11
   1.1. Introduction 11
   1.2. Risk Stratification 11
      1.2.1. Standard Risk 12
      1.2.2. Intermediate Risk 12
      1.2.3. High Risk 12
   1.3. The UKCCLG – I-BFM collaborative approach using MRD 14
      1.3.1. Guidelines for Intermediate Risk Patients where the MRD status is unknown 14
   1.4. Study Design 15
   1.5. Therapeutic Strategies for different risk groups 15
   1.6. Chemotherapy 17
      1.6.1. Induction (Phase I) 17
      1.6.2. Consolidation Block (Phase II) 18
      1.6.3. Intensification Block (Phase III) 18
   1.7. Bone Marrow Transplant Guidelines 18
      1.7.1 Cytoreduction Pre-Transplant 19
      1.7.2 Prevention of Infection during pre transplant cytoreduction 20
      1.7.3 Toxicity Monitoring 20
      1.7.4 Immunotherapy post transplantation 20
   1.8. Chimerism after SCT for ALL 21
   1.9. Special Categories 22
      1.9.1. CNS Disease at relapse and proceeding to chemotherapy alone 22
      1.9.2. Testicular disease at relapse and proceeding to chemotherapy alone 22

2. REFERENCES 23

3. OBJECTIVES 26
   3.1. Primary 26

NOT FOR CLINICAL USE

3.2. Secondary 26

4. STUDY POPULATION 26
4.1. Number of Subjects 26

5. ELIGIBILITY CRITERIA 26
5.1. Inclusion Criteria 26
5.2. Exclusion Criteria 26

6. ASSESSMENT PRIOR TO STARTING TREATMENT 27

7. DEFINITIONS OF MARROW STATUS AND RESPONSE 27

8. CHEMOTHERAPY SCHEDULES 28
INDUCTION 28
8.1. Remission Induction : Phase I : Weeks 1-4 30
CONSOLIDATION 32
8.2. Consolidation : Phase II : Weeks 5 – 8 34
INTENSIFICATION 37
8.3. Intensification : Phase III : Weeks 9-13 39
FLAD 41
Phase IV 41
8.4. Pre SCT Cytoreduction – Phase IV FLAD 43
INTERIM MAINTENANCE 44
8.5. Interim Maintenance : Phase V : Weeks 14-29 49
MAINTENANCE 51
8.6. Phase VI : Maintenance 54

9. ALLOGENEIC STEM CELL TRANSPLANT GUIDELINES 56
9.1. Eligibility 56
9.2. Protocol Outline 56
9.3. Definition of Matching 56
9.4. Cell Dose 56
9.5. Conditioning Regimens and GvHD Prophylaxis 57
9.5.1. Matched Sibling Marrow Transplants (Protocol A) 57
9.5.2. Matched Unrelated Marrow Transplants (Protocol B) 57
9.5.3. Mismatched Unrelated Marrow Transplants (Protocol C) 58
9.5.4. Haplo-identical Transplants (Protocol D) 58
9.5.5. Sibling PBSC and Matched Unrelated PBSC Transplants 58

10. ADOPTIVE IMMUNOTHERAPY 59
10.1. Eligibility 59
10.2. Definition of Chimeric Status 59
10.3. Grades of GvHD 59
10.4. Definition of Response 59
10.5. Cessation of Immune Suppression 59

NOT FOR CLINICAL USE

10.6. Immunotherapy Study Design ... 60
10.7. DLI Schedule ... 60
10.8. Data Collection ... 61

11. TRIAL MANAGEMENT AND REPORTING ... 62
11.1. National Participating Centres (United Kingdom and Ireland) ... 62
11.2. International Participating Centres and Contact Details ... 62
11.3. Randomisation ... 63
11.4. Data Monitoring Committee (DMC) ... 63
11.5. Reports ... 63
11.6. Toxicity Monitoring ... 63
11.6.1. Adverse Events (AE) ... 64
11.6.2. Adverse Reaction (AR) ... 64
11.6.3. Unexpected Adverse Reaction (UAR) ... 64
11.6.4. Serious Adverse Event (SAE) ... 64

12. STATISTICAL CONSIDERATIONS ... 67
12.1. Primary Analysis ... 67
12.2. Secondary Analysis ... 67
12.3. Sample Size and Analyses ... 67
12.4. Stopping Rule ... 68

APPENDICES ... 70

APPENDIX 1 : ANTIFUNGAL PROPHYLAXIS ... 70

APPENDIX 2 : BIOLOGICAL SAMPLES AND TESTS ... 72

APPENDIX 3 : GUIDELINES FOR INTERMEDIATE DOSE INTRAVENOUS ADMINISTRATION OF METHOTREXATE ... 77
Glucarpidase (formerly Carboxypeptidase) and Methotrexate Nephrotxicity79

APPENDIX 4 : RADIOTHERAPY ... 80

APPENDIX 5 : MERCAPTOPURINE AND METHOTREXATE DOSE ALTERATIONS ... 84

APPENDIX 6 : PNEUMOCYSTIS CARINII PNEUMONITIS (PCP) PROPHYLAXIS ... 86

APPENDIX 7 : GUIDANCE ON THE USE OF ERWINIA ... 88

APPENDIX 8 : ASPARAGINASE STUDY ... 89

ASPARAGINASE AND AEP STUDY FORM ALLR3 ... 91

NOT FOR CLINICAL USE

APPENDIX 9: GUIDELINES FOR THE INVESTIGATION AND MANAGEMENT OF THROMBO-EMBOLIC EVENTS 95

APPENDIX 10 : DRUG DETAILS 99

APPENDIX 11: DETERMINING WEIGHT FOR DOSAGE CALCULATIONS .. 107

APPENDIX 12 : DRUG INTERACTIONS WITH CYTOTOXIC AGENTS 109

APPENDIX 13: DRUG TOXICITIES AND DOSAGE MODIFICATIONS 111

13A. DEXAMETHASONE 111

13B. VINCRISTINE 111

13C. ASPARAGINASE 112

13D. IDARUBICIN 112

13E. MITOXANTRONE 113

13F. INTRATHECAL METHOTREXATE 113

13G. INTRAVENOUS METHOTREXATE 114

13H. ORAL HIGH DOSE METHOTREXATE 114

13I. CYCLOPHOSPHAMIDE 114

13J. HIGH DOSE CYTARABINE (ARA-C) 114

13K ETOPOSIDE 115

13.L. MERCAPTOPURINE 115

APPENDIX 14: HEALTH ECONOMIC CONSIDERATIONS 116

APPENDIX 15: ETHICAL COMMITTEES 117

APPENDIX 16: PATIENT INFORMATION SHEETS AND INFORMED CONSENT FORMS 119

APPENDIX 17: COMMON TOXICITY CRITERIA 142

APPENDIX 18: DECLARATION OF HELSINKI 144

NOT FOR CLINICAL USE

ABBREVIATIONS

Allo –SCT	Allogeneic Stem Cell Transplant
ALL	Acute Lymphoblastic Leukaemia
BFM	Berlin-Frankfürt-Münster
BMT	Bone Marrow Transplant
CCR	Complete Clinical Remission
CNS	Central Nervous System
CR	Complete Remission
CSF	Cerebrospinal Fluid
DFS	Disease Free Survival
DLI	Donor Lymphocyte Infusions
DMC	Data Monitoring Committee
DNA	Deoxyribonucleic Acid
DNR	Daunorubicin
DNX	DaunoXome
ECG	Electrocardiogram
EFS	Event Free Survival
FLAD	Fludarabine, High dose Cytosine and Liposomal Daunorubicin
FLAG	Fludarabine, Cytosine with GCSF
G-CSF	Granulocyte-Colony Stimulating Factor
GvHD	Graft versus Host Disease
GvL	Graft versus Leukaemia
kg	kilograms
KM	Kaplan Meier
MC	Mixed Chimerism
mg	milligrams
MHRA	Medicines and Healthcare Products Regulatory Authority
MRC	Medical Research Council
MRD	Minimal Residual Disease
MREC	Multi-centre Research Ethics Committee
MTD	Maximum Tolerated Dose
PCR	Polymerase Chain Reaction
PEG	Polyethylene glycol
PFS	Progression Free Survival
RNA	Ribonucleic Acid
SAE	Serious Adverse Event
SCT	Stem Cell Transplant
SUSAR	Suspected Unexpected Serious Adverse Reaction
TRM	Treatment Related Mortality
UKCCSG	United Kingdom Childhood Cancer Study Group
NOW CALLED CCLG	CHILDRENS CANCER AND LEUKAEMIA GROUP
UKCLWP	United Kingdom Childhood Leukaemia Working Party

NOT FOR CLINICAL USE

1. TRIAL OVERVIEW AND RATIONALE

1.1. *Introduction*

This protocol standardises the treatment of children with relapsed ALL who fail induction or relapse once a morphological remission has been obtained. It is evident from past experience that these children do not form one single clinical or biological entity. The proposed new therapy builds upon the results achieved in the MRC R1 protocol (1) and also utilises risk group stratification adopted by the BFM group (2).

Our current understanding of acute lymphoblastic leukaemia (ALL) suggests that initial induction and intensification results in a dramatic decrease in the tumour load (3). The more speedily this is achieved, the better the therapeutic outcome (4). However, at least a two-year period of maintenance therapy may be required to maintain remission (5). It is likely therefore, for maintenance to work that the level of disease must be decreased to an optimum level, currently below the level of standard diagnostic tools. The key drugs and the dosages used in maintenance therapy are not specifically cytocidal for lymphoblasts but do appear to achieve a state of immunosuppression. It is possible that they stimulate immune-mediated pathways or alter the stroma upon which malignant cells thrive in order to eradicate or control the malignant clone. To be able to do so effectively requires a minimum lymphoblast: effector cell ratio. From our past experience with relapsed ALL, such an effect is probably strongest in the marrow and less so in extramedullary sites.

Given this background, those who relapse early are likely to have more drug-resistant disease as primary treatment failed to decrease the initial tumour load adequately. These children may benefit from more intense induction, intensification and possibly from bone marrow transplantation (6). Those who relapse later may do so because they are unable to mount a proper immune response. Such children may benefit from a more direct lymphotoxic maintenance schedule.

The place of bone marrow transplant remains uncertain in ALL. However results from the MRC and BFM suggest the groups that benefit most are those with early bone marrow relapse and those with T cell disease. The CCLG BMT group guidelines are proposed as an integral part of this protocol in order to standardise therapy throughout the United Kingdom and Ireland.

1.2. *Risk Stratification*

The working party has previously identified the risk categories for relapsed ALL on an intent to treat basis. In our comparison of risk stratifications, we find that the International BFM Study Group (I-BFM-SFG) has the most similar model of treatment. To establish a common approach to these children throughout Europe, the R3 protocol adopted a simplified BFM classification in 2003 at the time of the launch of the R3 protocol. This classified the patients into Standard, Intermediate and High Risk groups, based on the time to relapse, site of relapse and immunophenotype. In this classification, those relapsing with bone marrow disease 18 months after diagnosis were classified as High Risk. Patients with very early extramedullary relapse were considered to be at Intermediate Risk. Subsequent analysis of patients treated on the R2 protocol between the years 1995-2002 suggests however that very early isolated extramedullary disease has a poor outcome (7). This has been subsequently supported by observations made in the first 3-years of the R3 trial and observations made by other groups in Europe (unpublished meeting reports, I-BFM Resistant Disease Subgroup).

NOT FOR CLINICAL USE

Thus, a new modified risk stratification has been adopted as shown below.

Figure 1

	Non-T			T-cell		
	Isolated EM	Combined	Isol Marrow	Isolated EM	Combined	Isol Marrow
Very Early	**H**	**H**	**H**	**H**	**H**	**H**
Early	**I**	**I**	**H**	**I**	**H**	**H**
Late	**S**	**I**	**I**	**S**	**H**	**H**

Very Early: Within 18 months of diagnosis; Early: After 18 months of diagnosis but within 6 months of stopping treatment; Late: More than 6 months after stopping treatment. EM= Extramedullary. Combined= Marrow and extramedullary involvement, Isol = Ioslated

1.2.1. *Standard Risk*

This category of patients will be treated with chemotherapy and localised radiotherapy, as per the ALLR3 protocol.

1.2.2. *Intermediate Risk*

Our previous analysis showed that this is the single largest group, comprising 70% of relapsed childhood ALL (7). The overall outcome of those in the intermediate risk group during 1995-2002 was 64% (7). However some patients in this group were treated with chemotherapy alone and others received an allogeneic stem cell transplant (allo-SCT). The considerable heterogeneity within the group precludes a single therapeutic option. In agreement with the I-BFM group, we speculate that those who clear disease quickly, as measured by minimal residual disease (MRD) techniques, are most likely to sustain remission without an allogeneic stem cell transplant (allo-SCT). Thus, in ALLR3 it is intended that those children who have a disease level of $<10^{-4}$ by 2 loci at the end of induction (day 35) will continue on chemotherapy with targeted radiotherapy if there is involvement of an extramedullary site. For those who have disease levels of $\geq10^{-4}$, if there is a matched donor, then the intention is for an allo-SCT. The BFM have adopted a similar strategy, but with a cut-off level of $<10^{-3}$.

About 30% of those in the intermediate-risk group have isolated extramedullary disease. In these patients, currently it is not possible to stratify according to MRD. A proportion of these patients have now been re-classified as high risk(7). Therapeutic options for the remainder will depend on time to relapse. For those relapsing within two years of completing therapy, allo-SCT is intended if there is a matched donor. For others the intention is to carry on with chemotherapy and targeted radiotherapy.

1.2.3. *High Risk*

For the non-T cell group, the previous recommended UK treatment was re-induction with R2 (identical to R1) and to proceed to either a matched sibling or unrelated donor transplant. In the absence of a donor, the recommendation was to continue with R2 chemotherapy. However, since these recommendations were made, haploidentical transplants are now being successfully performed by a number of units (8). Moreover, the recently published results of the R1 trial (1), suggests a survival advantage in those transplanted over those who received chemotherapy in the

NOT FOR CLINICAL USE

high-risk group (9). In the ALLR3 protocol, the recommendation for this group is to proceed to transplant after chemotherapy [see guidelines for allogeneic-Stem Cell Transplant] (10).

A number of studies have shown that those with high levels of disease prior to allo-SCT usually relapse (11-13). Thus in common with the I-BFM, the ALLR3 patients will have a MRD assessment at week 13. Those with disease levels of $\geq 10^{-3}$, will be offered an additional intensive phase, namely FLAD, prior to allo-SCT.

The treatment plan for those with isolated extramedullary disease has been debated in the I-BFM-SG Resistant disease committee. Our understanding is that systemic therapy is the key to the control of disease, whether medullary or extramedullary. Unpublished observations from previous UK trials suggest that a matched donor allo-SCT may be the best therapeutic option, as clearly chemotherapy results are poor (7). Thus for these children, where there is a matched donor available, the recommended treatment is an allo-SCT.

NOT FOR CLINICAL USE

1.3. *The UKCCLG – I-BFM collaborative approach using MRD*

This protocol is based on the premise that detection of disease at the molecular level, at specified time-points will influence the outcome of treatment. In collaboration with the I-BFM the following have been agreed on:

MRD assessment will be done using Real-Time PCR for IgH and TCR rearrangements according to pan European guidelines. Four laboratories have been designated to carry this out in the UK and will be coordinated by Dr Nick Goulden. The MRD result from week 5 will be available by week 8 and for week 13/14 MRD, results will be available by week 14/15.

ii. MRD assessment time points will be the same for all patients treated on the I-BFM and the ALLR3 protocol.

The same risk categories as earlier defined will be used:

All standard risk children will be treated with chemotherapy and radiotherapy.

For intermediate risk patients, allo-SCT will be offered to those who are MRD positive ($>10^{-4}$) at the end of induction (week 5). Intermediate risk patients who are MRD negative (2 targets sensitive to 10^{-4}) will receive continuing chemotherapy. If MRD $>10^{-3}$ at Week 13, they will be recommended FLAD prior to allo-SCT.

All high risk patients will be offered an allo-SCT irrespective of MRD status at week 5. If MRD $>10^{-3}$ at Week 13, they will be recommended FLAD prior to allo-SCT.

All patients scheduled to go onto allo-SCT will have MRD assessment at count recovery at the end of the consolidation block (week 13-14). In those with MRD $\geq 10^{-3}$, an additional block of treatment using Fludarabine, High-dose Cytosine and liposomal Daunorubicin (FLAD) will be used to see if we can decrease tumour load further and if this decrease improves outcome of allo-SCT compared with historical controls.

1.3.1. *Guidelines for Intermediate Risk Patients where the MRD status is unknown*

In some patients it will not be possible to identify a target for MRD. These will be defined as MRD Indeterminate. This category includes: (i) all those with isolated extramedullary disease; (ii) those in whom MRD assessment failed; (iii) those in whom MRD of $<10^{-4}$ was detected at only one loci.

Bone Marrow Relapse

If relapse has occurred within 48 (girls) or 60 (boys) months of the first diagnosis and a matched donor is available, proceed to allo-SCT.

If relapse has occurred after 48 (girls) or 60 (boys) months from the first diagnosis, or a matched donor is not available, continue on chemotherapy.

Isolated Extramedullary Relapse

If relapse has occurred within 48 (girls) or 60 (boys) months of the first diagnosis and a matched donor is available, proceed to allo-SCT.

NOT FOR CLINICAL USE

If the relapse has occurred after 48 (girls) or 60 (boys) months from the first diagnosis, or a matched donor is not available, continue on chemotherapy and directed radiotherapy.

In children who have previously received cranial irradiation, high dose methotrexate is considered inadequate therapy. However, two courses of cranial irradiation is toxic. In a previous survey, 20% of such relapses could be salvaged but 60-70% subsequently required special schooling. In these rare cases, please consult one of the Trial Co-ordinators.

1.4. *Study Design*

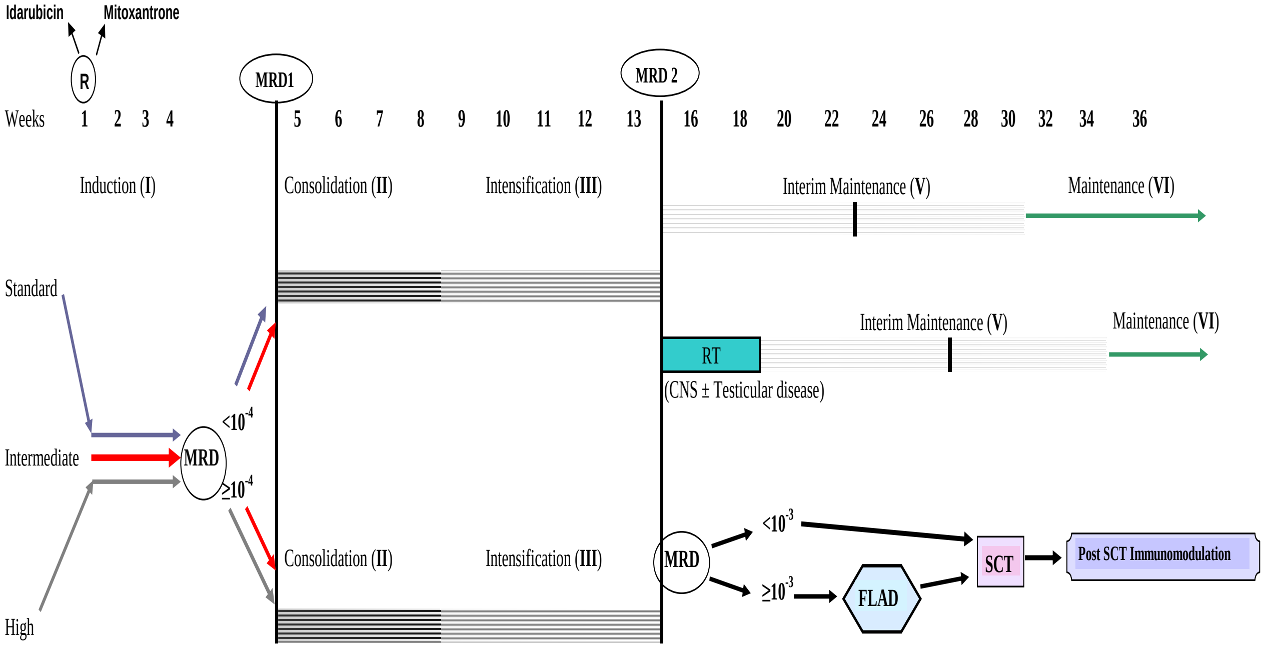

Figure 2.

NOT FOR

1.5. *Therapeutic Strategies for different risk groups*

Standard ⇨ Induction, Consolidation, Intensification & Maintenance

Intermediate ⇨ Induction, Consolidation and Intensification followed by

MRD $<10^{-4}$ (Timepoint 1) continue chemotherapy with Phase V (Interim Maintenance) and then Phase VI (Maintenance).

MRD $\geq 10^{-4}$ (Timepoint 1), proceed to allo-SCT if matched donor available and reassess MRD at Week 13/14 (after consolidation).

High ⇨ Induction, Consolidation and Intensification followed by

MRD assessment Timepoint 2 (post Consolidation Week 13/14).

MRD $<10^{-3}$ proceed to allo-SCT post Intensification (Phase III).

MRD $\geq 10^{-3}$ proceed to FLAD (Phase IV) followed by allo-SCT.

Donor options will include matched Sibling, Unrelated or Haploidentical donors. In those children where MRD status is unknown, only high risk children should be considered for haploidentical donors at present.

All patients are eligible on study entry for randomisation to receive either Idarubicin or Mitoxantrone during Induction.

NOT FOR CLINICAL USE

1.6. *Chemotherapy*

1.6.1. *Induction (Phase I)*

Choice of Steroids and dosing schedule

ALLR3 will use orally administered dexamethasone throughout instead of prednisolone. Data suggests that Dexamethasone has better CNS penetration because it is less protein bound (14) and may be more effective than prednisolone in preventing CNS relapse, improving event-free survival (15, 16). Dexamethasone appears to be more cytotoxic to lymphoblasts than prednisolone but the difference ranges from 5.5 to 16.2 fold, and the optimal dosage is unknown (17, 18). Evidence however suggests that a higher dose of steroids improves outcome (19).

There are two possible ways of administering the steroid. Historically, short duration steroid exposure has been shown to be less effective as a single agent for induction. However, intermittent pulses of dexamethasone have been employed in BFM relapse trials, where dexamethasone has been given at 20mg/m^2 for 5 days during each block. Potentially pulsed dosing may diminish receptor down-regulation seen with prolonged glucocorticoid exposure and decrease toxicity (20). We have therefore decided to retain the BFM block of Dexamethasone at 20 mg/m^2/day (in two divided doses) for 5 days from day 1-5 and again on days 15-19 of induction. Once remission has been achieved, 5-day blocks of Dexamethasone will be used at 6mg/m^2/day in two divided doses for 5 days and stopped without tapering.

Idarubicin vs. Mitoxantrone

Idarubicin is an anthracycline analogue with demonstrated efficacy in the treatment of ALL. Our present ATP assay data in ALL, particularly in relapsed or refractory patients suggest that both Idarubicin and Mitoxantrone have increased efficacy against lymphoblasts when compared to the more commonly used Daunorubicin and Doxorubicin. Idarubicin is thought to be less cardiotoxic (21). Its superior efficacy when compared to other anthracyclines has been attributed to its active metabolite, idarubicinol, which persists in the circulation and penetrates the CNS (22). The CCG 1884 trial for relapsed ALL compared idarubicin (12.5mg/m^2/week) to doxorubicin (45mg/m^2/week) during re-induction. Two year EFS was significantly better among patients who received idarubicin (27+/-18%) compared to those who received doxorubicin (10+/-8%). However, this dosing schedule resulted in unacceptable haematopoietic toxicity with a 19% induction death rate due to infection (23). To decrease toxicity we propose to administer idarubicin on the initial two days of induction. This dosing schedule should minimise prolonged myelosuppression. The dose of Idarubicin used in children has varied from 5 – 15 mg/m^2. It does not seem likely that doses over 12 mg/m^2 offer therapeutic advantage. The trial therefore proposes to use 10 mg/m^2, infused over 1 hr on d1 and d2. The group has considerable experience with Mitoxantrone in the context of AML12 and the drug is also effective in ALL (24). We will use the same dose of Mitoxantrone as used in the AML trials, that is 10mg/m^2.

PEG-Asparaginase

Previously children and young adults with ALL in the UK received Erwinia asparaginase. In the current frontline protocol they are now receiving PEG-Asparaginase. There is data to suggest that both peak and trough levels of asparaginase are related to the degree of asparagine depletion (25) and arguably a long acting asparaginase should be used in induction. At the present moment, the data suggests that an asparaginase activity of >100 IU/l during the induction period is required to achieve complete asparagine depletion in the serum. In the UK

NOT FOR CLINICAL USE

and in Germany, in heavily pre-treated patients, this can be achieved by using PEG-Asparaginase (Medac – Oncaspar) at 1000 u/m^2 on a fortnightly basis (26).

Other drugs

Intrathecal methotrexate will be given on d1 and d8 in all patients (see recommendations for children with CNS disease in reference section) at the current recommended doses. Vincristine at 1.5 mg / m^2 intravenously on d 3, 10, 17, and 24.

Note the days of Vincristine have been deliberately shifted to avoid any risk of intrathecal administration, and not for any therapeutic reasons.

1.6.2. *Consolidation Block (Phase II)*

This block is radically different from that used in the R1-R2 protocols. Although the majority of children with relapsed ALL achieve second remission, the depth of this remission is shallow. To improve the duration of second remission, intensive blocks of therapy have to be utilised in re-induction.

At the start of the block, an additional dose of Vincristine and steroids is given. Some children may experience a delay in receiving the first consolidation block, as they may not have recovered their counts. Therefore, these drugs are not count dependent and can be administered irrespective of counts.

Intermediate dose methotrexate with delayed folinic acid rescue is incorporated into the first block of intensification as this dosing schedule has been shown to be superior to high dose methotrexate with folinic acid rescue beginning at hour 24 on the BFM REZ'85 trial (27). Asparaginase will be given after the Methotrexate infusion to potentiate the effect (28).

In ALL97/99, Etoposide has not been used. It would be logical to use this in R3 in the next phase of treatment. The BFM protocol uses Ifosfamide, however we have preferred to stay with Cyclophosphamide as in R2. The combination of Etoposide and Cyclophosphamide / or Ifosfamide has been well tolerated in this setting and the proposed dosing and schedule for Cyclophosphamide and Etoposide are in use in many protocols worldwide.

1.6.3. *Intensification Block (Phase III)*

In the final block, we propose to use sequential high dose Ara-C and L-Asparaginase. This regimen has been shown to be effective in refractory disease with 45% of children with relapsed ALL who failed standard therapy achieving CR for 1-5 months, and is used by both the BFM and COG groups (23, 29, 30).

Those who will receive radiotherapy will do so after they have completed all three phases of chemotherapy. Those who are to be transplanted will now do so according to national transplant guidelines based on the type of donor (see Transplant Guidelines on the following page). The rest will receive continuing therapy.

1.7. *Bone Marrow Transplant Guidelines*

Analysis of the results of the MRC UKALL R1 protocol indicate that prognosis following relapse is a function of the duration of CR1 and the site of recurrence (1). Thus while the overall 5 year event free survival of children treated according to R1 was 46%, it was only 7% for those suffering a bone marrow relapse on therapy. This is in marked contrast to the 77% EFS seen in those children relapsing without bone marrow involvement more than 2.5 years from diagnosis (1). Previous MRC studies have failed to delineate the role of allogeneic-SCT in the treatment of recurrent ALL. Nevertheless current CCLG guidelines mandate that allo-SCT (sibling or

NOT FOR CLINICAL USE

unrelated donor) is appropriate for those children who relapse in the bone marrow within two years of completion of therapy.

UK results of allogeneic SCT in CR2 of ALL suggest that children who suffer a BM relapse during therapy have a dismal prognosis even with allogeneic -SCT. Clearly there is a need to devise novel therapies for this group of children. A potential avenue for further investigation has been revealed by analysis of MRD immediately prior to conditioning for allo-SCT (11). Twenty-nine children who suffered a bone marrow relapse were studied retrospectively. All had received primary therapy according to the MRC UKALL X/XI trials. Following relapse they were treated according to the MRC relapse protocol (R1/R2). All entered remission within one month of relapse. Allo-SCT was carried out following consolidation according to the R1 protocol. MRD was examined in the bone marrow immediately prior to conditioning that consisted of Cyclophosphamide 120mg/kg & TBI 1440Gy in 8 fractions. Marrow from unrelated donors (n=25) were T-cell depleted with Campath 1M and sibling marrows were unmanipulated. The correlation between MRD status, relapse and duration of first remission is shown in the Table 3.

Table 3

**CR1 Duration**	**TRM**	No of relapses / No with given MRD result<br>**Negative**	**< 1/1000**	**>1/1000**	**EFS**
<24/ 12	0	1/ 2	2/ 2	7/ 7	9%
24-30/ 12	2	4/ 7	0/ 0	1/ 1	25%
30-48/ 12	1	0/ 8	0/ 1	1/ 1	90%
Total	3	5/ 17	2/ 3	9/ 9	

The most important observation from this study is that disease recurred in every child with evidence of high level MRD, i.e. more than one leukaemic cell in one thousand. It can also be seen that persistence of high level MRD after R1 consolidation is more common in children with short duration of CR1, a surrogate marker of resistant disease. By contrast only a minority of children who relapse after therapy have high level MRD pre allo-SCT.

1.7.1 Cytoreduction Pre-Transplant

All intermediate and high risk patients who have a level of disease $\geq 10^{-3}$ at week 13 are eligible for additional cytoreduction therapy prior to allo-SCT. This treatment will begin by week 16 of relapse therapy. At the time of writing this protocol, this will be FLAD. Given the nature of this therapy, its efficacy and toxicity will be closely monitored. If it does not prove to be beneficial and/or a more suitable agent (s) are identified, this phase of the trial may be subject to change.

FLAD [FLAG/DaunoXome (liposomal daunorubicin)]

The initial window therapy will be FLAD. This will be given around week 16. FLAG is a combination of two chemotherapeutic agents, fludarabine and cytosine, together with G-CSF.

NOT FOR CLINICAL USE

The theoretical advantage of the FLAG combination is that fludarabine enhances the intra-cellular cytotoxicity of high dose cytosine without increasing its systemic toxicity. G-CSF is used primarily to hasten neutrophil recovery post chemotherapy. FLAG is used extensively in the treatment of acute myeloid leukaemia (AML) and relapsed ALL where it has been shown to have significant anti-leukaemic activity (31). DaunoXome (DNX) (liposomal daunorubicin) is a liposomal preparation of Daunorubicin (DNR) (32, 33).

Most children with relapsed ALL will already have been pre-treated with anthracyclines. In this context DaunoXome (liposomal daunorubicin) provides theoretical benefits. The liposomal preparation allows significantly higher doses to be given than the conventional formulation, which may increase its antileukemic effect. Phase I studies have shown that the maximum tolerated dose (MTD) of DNX in children is approximately 125 mg/m^2 in heavily pre-treated patients and in excess of 155mg/m^2 in others (MTD of DNR ~50mg/m^2). This MTD is using drug-induced myelosuppression as the dose limiting toxicity (DLT). This is not standard practice in leukaemias where extra-medullary toxicities are usually used. Studies in adults suggest that the MTD for leukaemic patients is 150mg/m^2 x 3 doses, with mucositis as the DLT. Caution must be used in considering the appropriate dose of DaunoXome (liposomal daunorubicin) in this context as both FLAG and total body irradiation may have added toxicities. It is planned that the initial intensification therapy will consist of FLAG and Liposomal daunorubicin 100mg/m2 (FLAG/DNX).

1.7.2 Prevention of Infection during pre transplant cytoreduction

It is recommended that infective prophylaxis should be instituted as per unit protocol prior to FLAD. This might include gut sterilisation with ciprofloxacin, low dose oral acyclovir, fungal prophylaxis with itraconazole and PCP prophylaxis with cotrimoxazole. In addition, all patients should receive irradiated blood products from this point onwards. Transplant conditioning should proceed 3 weeks from the beginning of FLAG/DNX, with a bone marrow aspirate for MRD studies taken immediately prior to the start of conditioning.

1.7.3 Toxicity Monitoring

Echocardiograms and ECGs should be performed prior to the window therapy, again prior to conditioning and upon release from protective isolation. In the event of repeat echocardiography showing greater than 20% reduction in ejection fraction the echo should be repeated a week later. The Chief Investigator should be informed of any cardiac toxicity noted, either clinically or on echocardiogram/ECG.

1.7.4 Immunotherapy post transplantation

Relapse is the most common cause of treatment failure after SCT for ALL in CR2. Simplistically, failure of SCT can be thought of as failure of high dose therapy and donor alloreactivity to overcome residual disease at the time of SCT. The duration of CR1, site of relapse and MRD load pre SCT are interrelated risks for relapse after SCT. ALLR3 is investigating whether intensified cytoreduction immediately pre SCT improves survival in patients with high level MRD pre conditioning.

An alternative/additional approach is to augment donor alloreactivity. There are two important caveats here. First, evidence of a GvL effect in ALL is less convincing than in other haematological malignancies. Second, the inherent danger of increased alloreactivity is an

NOT FOR CLINICAL USE

increase in graft versus host disease (GvHD), transplant related mortality (TRM) and long-term morbidity. Consequently researchers have endeavoured to define whether analysis of chimerism and MRD following allo-SCT can highlight a group of children and young adults at highest risk of relapse. This group would then be candidates for immunotherapy using relatively low doses of donor lymphocyte infusions (DLI). ALLR3 aims to generate a common protocol for monitoring and intervention after allo-SCT.

1.8. *Chimerism after SCT for ALL*

Mixed chimerism (MC) is defined as a greater than 5% increase in recipient cells and is more common after T-cell depleted grafts. In single time point analyses MC is associated with an increased risk of relapse. Dynamic assessment of chimeric status is therefore more informative than single time point analysis. Weekly analysis of peripheral blood chimerism until day 200 and then monthly until 18 months is currently deemed optimal. Increasing MC may be reversed by cessation of immune suppression or DLI, though the interval between the development of increasing MC and relapse may be so short as to preclude clinically useful intervention.

The only large study published to date of monitoring MC and the use of DLI, is that of Bader et al (34). However it is important to note that this is a highly selected heterogeneous cohort of patients. In particular children transplanted in CR1 are relatively over represented in those responding to immune modulation. Moreover more than 50% of children undergoing immunotherapy developed GvHD and 16% of these died from GvHD. Interestingly, the development of overt GvHD was not associated with a decreased risk of relapse in those children undergoing immunotherapy for increased MC, suggesting that overt GvHD is not required for a clinically significant graft versus leukaemia (GvL) effect.

The most widely applicable method for the analysis of chimerism is PCR of short tandem repeat sequences (STR). The sensitivity of this approach is 1% which is ideal for monitoring (35). More sensitive techniques cause confusion with amplification of residual host stromal DNA. STR PCR is quantitative as it is a competitor PCR. Importantly standardised methods of STR PCR are now available as part of the European chimerism collaboration. Although much of the early work used marrow, the most compelling data about the correlation between mixed chimerism and risk relapse has used PCR of blood. The ready availability of samples and the small volumes required (2mls is often sufficient) mean that blood is currently the optimal sample for analysis.

MRD analysis offers additional sensitivity and specificity when compared with chimeric analysis. Theoretically regular analysis of MRD post SCT should allow earlier detection of relapse and initiation of immunotherapy at lower levels of disease. As in CML this may then lead to more successful immunotherapy with less GvHD. In 1998 Knechtli published the Bristol experience of radiolabelled probe based MRD when measured in marrow at 1,2,3,6,9 and 12 months after SCT in 71 children (11). The salient findings were that MRD was present in 88% of children destined to relapse, a median of 3 months prior to recurrence. MRD was also detected in 22% of those who remained in long-term remission. In all these cases MRD was present at a level of 0.01-0.001% and at least two subsequent negative tests were documented in each child.

At the I-BFM meeting in May 2005, Bader presented as yet unpublished data of the use of RQ PCR technology to analyse MRD in marrow at day 30, 60, 100, 180 and 360 days post SCT in CR2. He demonstrated that MRD was present prior to relapse in more than 90% of children and

NOT FOR CLINICAL USE

that levels of >1/1,000 were universally associated with relapse. Bader also looked at MRD levels at the time of immunotherapy initiated on the basis of increasing MC. Immunotherapy failed in all 7 cases where MRD was >1/1000 at the initiation of immune modulation. However 8 of 18 children, in whom immunotherapy was initiated at a time when marrow MRD was less than 1/1000, are in remission.
Regular marrow sampling presents major logistical difficulties in children after SCT. Bader and others have examined whether blood can be substituted for MRD analysis. It has now been shown that the presence of MRD at a level greater than 1/10,000 (30 of 95 patients) in the blood is associated with a 100% risk of relapse (Bader, I-BFM, 2005). Thus ALL R3 proposes to monitor peripheral blood/marrow for MC and intervene in these cases with immunomodulation.

NOT FOR CLINICAL USE

1.9. *Special Categories*

1.9.1. *CNS Disease at relapse and proceeding to chemotherapy alone*

Patients with CNS disease at diagnosis (the presence of >5/cumm unequivocal lymphoblasts in the CSF) should receive weekly intrathecal methotrexate until two consecutive clear CSF's have been obtained. Patients not being transplanted should receive cranial irradiation 24 Gy in 15 fractions of 1.6 Gy each of cranial radiotherapy starting week 14. Following radiotherapy they should not receive any further intrathecal methotrexate (see Appendix 4). The individual regimens provide specific timing and dosages. These patients are still eligible for the trial randomisation.

There is evidence to suggest that the use of thiopurines during cranial irradiation may predispose to the occurrence of brain tumours. Therefore during cranial radiotherapy, only the use of vincristine and dexamethasone is recommended.

NB: Children under 2 years of age with CNS disease at diagnosis are not eligible for cranial radiotherapy. We anticipate that this will be a rare occurrence. If you do have this problem, please discuss it with the Chief Investigator.

Formulation of IT MTX and post-LP care
Some centres may be using a highly concentrated formulation of Methotrexate which results in insufficient volume to fill the dead space and reach ventricular spaces. Methotrexate for intra-thecal use should be made up at a maximum concentration of 2.5mg/ml so as to provide an adequate volume of distribution across the CNS.

We recommend laying the patient supine for at least 1 hr after the intra-thecal procedure. Experiments in primate models indicate better ventricular distribution of intra-thecal chemotherapy if the subject lies supine for this period after the procedure.

1.9.2. *Testicular disease at relapse and proceeding to chemotherapy alone*

Boys with testicular infiltration at presentation should follow the protocol. Those not being transplanted should have 24Gy in 12 daily fractions of irradiation to both testes starting week 14. Other treatment should continue uninterrupted (see Appendix 5), during the period of Radiotherapy.

2. REFERENCES

1. Lawson SE, Harrison G, Richards S, Oakhill A, Stevens R, Eden OB, et al. The UK experience in treating relapsed childhood acute lymphoblastic leukaemia: a report on the medical research council UKALLR1 study. Br J Haematol 2000; 108(3): 531-43.

2. Borgmann, A., von Stackelberg, A., Hartmann, R., Ebell, W., Klingebiel, T., Peters, C. & Henze, G. (2003) Unrelated donor stem cell transplantation compared with chemotherapy for children with acute lymphoblastic leukemia in a second remission: a matched-pair analysis. Blood, 101**,** 3835-3839.

3. Schrappe M, Reiter A, Zimmermann M, Harbott J, Ludwig WD, Henze G, et al. Long-term results of four consecutive trials in childhood ALL performed by the ALL-BFM study group from 1981 to 1995. Berlin-Frankfurt-Munster. Leukemia 2000;14(12):2205-22.

4. Lilleyman JS. Clinical importance of speed of response to therapy in childhood lymphoblastic leukaemia. Leuk Lymphoma 1998;31(5-6):501-6.

5. Duration and intensity of maintenance chemotherapy in acute lymphoblastic leukaemia: overview of 42 trials involving 12 000 randomised children. Childhood ALL Collaborative Group. Lancet 1996;347(9018):1783-8.

6. Hann I, Vora A, Richards S, Hill F, Gibson B, Lilleyman J, et al. Benefit of intensified treatment for all children with acute lymphoblastic leukaemia: results from MRC UKALL XI and MRC ALL97 randomised trials. UK Medical Research Council's Working Party on Childhood Leukaemia. Leukemia 2000;14(3):356-63.

7. Roy A, Cargill A, Love S, Moorman AV, Stoneham S, Lim A, Darbyshire PJ, Lancaster D, Hann I, Eden T, Saha V. Outcome of first relapse in childhood acute lymphoblastic leukaemia - experiences from the UKALL R2 protocol. Br J Haematol 2005;130:60-75.

8. Veys PA, Meral A, Hassan A, Goulden N, Webb D, Davies G. Haploidentical related transplants and unrelated donor transplants with T cell addback. Bone Marrow Transplant 1998;21 Suppl 2:S42-4.

9. Harrison G, Richards S, Lawson S, Darbyshire P, Pinkerton R, Stevens R, et al. Comparison of allogeneic transplant versus chemotherapy for relapsed childhood acute lymphoblastic leukaemia in the MRC UKALL R1 trial. MRC Childhood Leukaemia Working Party. Ann Oncol 2000;11(8):999-1006.

10. Dini G, Cornish JM, Gadner H, Souillet G, Vossen JM, Paolucci P, et al. Bone marrow transplant indications for childhood leukemias: achieving a consensus. The EBMT Pediatric Diseases Working Party. Bone Marrow Transplant 1996;18 Suppl 2:4-7.

11. Knechtli, C.J., Goulden, N.J., Hancock, J.P., Grandage, V.L., Harris, E.L., Garland, R.J., Jones, C.G., Rowbottom, A.W., Hunt, L.P., Green, A.F., Clarke, E., Lankester, A.W., Cornish, J.M., Pamphilon, D.H., Steward, C.G. & Oakhill, A. (1998) Minimal residual disease status before allogeneic bone marrow transplantation is an important determinant of successful outcome for children and adolescents with acute lymphoblastic leukemia. Blood, **92,** 4072-4079.

12. Krejci, O., van der Velden, V.H., Bader, P., Kreyenberg, H., Goulden, N., Hancock, J., Schilham, M.W., Lankester, A., Revesz, T., Klingebiel, T. & van Dongen, J.J. (2003) Level of minimal residual disease prior to haematopoietic stem cell transplantation predicts

NOT FOR CLINICAL USE

prognosis in paediatric patients with acute lymphoblastic leukaemia: a report of the Pre-BMT MRD Study Group. Bone Marrow Transplantation, 32, 849-851.

13. Bader, P., Hancock, J., Kreyenberg, H., Goulden, N.J., Niethammer, D., Oakhill, A., Steward, C.G., Handgretinger, R., Beck, J.F. & Klingebiel, T. (2002) Minimal residual disease (MRD) status prior to allogeneic stem cell transplantation is a powerful predictor for post-transplant outcome in children with ALL. Leukemia, 16, 1668-1672.

14. Balis FM, Lester CM, Chrousos GP, Heideman RL, Poplack DG. Differences in cerebrospinal fluid penetration of corticosteroids: possible relationship to the prevention of meningeal leukemia. J Clin Oncol 1987;5(2):202-7.

15. Silverman LB, Gelber RD, Dalton VK, Asselin BL, Barr RD, Clavell LA, et al. Improved outcome for children with acute lymphoblastic leukemia: results of Dana-Farber Consortium Protocol 91-01. Blood 2001;97(5):1211-8.

16. Gaynon PS, Trigg ME, Heerema NA, Sensel MG, Sather HN, Hammond GD, et al. Children's Cancer Group trials in childhood acute lymphoblastic leukemia: 1983-1995. Leukemia 2000;14(12):2223-33.

17. Kaspers GJ, Veerman AJ, Popp-Snijders C, Lomecky M, Van Zantwijk CH, Swinkels LM, et al. Comparison of the antileukemic activity in vitro of dexamethasone and prednisolone in childhood acute lymphoblastic leukemia. Med Pediatr Oncol 1996;27(2):114-21.

18. Ito C, Evans WE, McNinch L, Coustan-Smith E, Mahmoud H, Pui CH, et al. Comparative cytotoxicity of dexamethasone and prednisolone in childhood acute lymphoblastic leukemia. J Clin Oncol 1996;14(8):2370-6.

19. Schwartz CL, Thompson EB, Gelber RD, Young ML, Chilton D, Cohen HJ, et al. Improved response with higher corticosteroid dose in children with acute lymphoblastic leukemia. J Clin Oncol 2001;19(4):1040-6.

20. Sanden S, Tripmacher R, Weltrich R, Rohde W, Hiepe F, Burmester GR, et al. Glucocorticoid dose dependent downregulation of glucocorticoid receptors in patients with rheumatic diseases. J Rheumatol 2000;27(5):1265-70.

21. Platel D, Pouna P, Bonoron-Adele S, Robert J. Comparative cardiotoxicity of idarubicin and doxorubicin using the isolated perfused rat heart model. Anticancer Drugs 1999;10(7):671-6.

22. Boogerd W, Tjahja IS, van de Sandt MM, Beijnen JH. Penetration of idarubicin into malignant brain tumor tissue. J Neurooncol 1999;44(1):65-9.

23. Leahey AM, Bunin NJ, Belasco JB, Meek R, Scher C, Lange BJ. Novel multiagent chemotherapy for bone marrow relapse of pediatric acute lymphoblastic leukemia. Med Pediatr Oncol 2000;34(5):313-8.

24. Koc Y, Akpek G, Kansu E, Kars A, Tekuzman G, Baltali E, et al. Bolus and continuous infusion mitoxantrone in newly diagnosed adult acute lymphoblastic leukemia: results of two consecutive phase II clinical studies. Cancer Invest 1998;16(6):355-65.

25. Woo MH, Hak LJ, Storm MC, Evans WE, Sandlund JT, Rivera GK, et al. Anti-asparaginase antibodies following E. coli asparaginase therapy in pediatric acute lymphoblastic leukemia. Leukemia 1998;12(10):1527-33.

NOT FOR CLINICAL USE

26. Muller HJ, Loning L, Horn A, Schwabe D, Gunkel M, Schrappe M, et al. Pegylated asparaginase (Oncaspar) in children with ALL: drug monitoring in reinduction according to the ALL/NHL-BFM 95 protocols. Br J Haematol 2000;110(2):379-84.

27. Dordelmann M, Reiter A, Zimmermann M, Fengler R, Henze G, Riehm H, et al. Intermediate dose methotrexate is as effective as high dose methotrexate in preventing isolated testicular relapse in childhood acute lymphoblastic leukemia. J Pediatr Hematol Oncol 1998;20(5):444-50.

28. Maul DM, Schuster SM. Methotrexate stimulation of asparagine synthetase activity in rat liver. Life Sci 1982;30(12):1051-7.

29. Harris RE, Sather HN, Feig SA. High-dose cytosine arabinoside and L-asparaginase in refractory acute lymphoblastic leukemia: the Children's Cancer Group experience. Med Pediatr Oncol 1998;30(4):233-9.

30. Millot F, Suciu S, Philippe N, Benoit Y, Mazingue F, Uyttebroeck A, et al. Value of high-dose cytarabine during interval therapy of a Berlin-Frankfurt-Munster-based protocol in increased-risk children with acute lymphoblastic leukemia and lymphoblastic lymphoma: results of the European Organization for Research and Treatment of Cancer 58881 randomized phase III trial. J Clin Oncol 2001;19(7):1935-42.

31. McCarthy AJ, Pitcher LA, Hann IM, Oakhill A. FLAG (fludarabine, high-dose cytarabine, and G-CSF) for refractory and high-risk relapsed acute leukemia in children. Med Pediatr Oncol 1999;32(6):411-5.

32. Cortes J, O'Brien S, Estey E, Giles F, Keating M, Kantarjian H. Phase I study of liposomal daunorubicin in patients with acute leukemia. Invest New Drugs 1999;17(1):81-7.

33. Bellott R, Auvrignon A, Leblanc T, Perel Y, Gandemer V, Bertrand Y, et al. Pharmacokinetics of liposomal daunorubicin (Liposomal daunorubicin) during a phase I-II study in children with relapsed acute lymphoblastic leukaemia. Cancer Chemother Pharmacol 2001;47(1):15-21.

34. Bader P, Kreyenberg H, Hoelle W, Dueckers G, Handgretinger R, Lang P, Kremens B, Dilloo D, Sykora KW, Schrappe M, Niemeyer C, Von Stackelberg A, Gruhn B, Henze G, Greil J, Niethammer D, Dietz K, Beck JF, Klingebiel T. Increasing mixed chimerism is an important prognostic factor for unfavorable outcome in children with acute lymphoblastic leukemia after allogeneic stem-cell transplantation: possible role for pre-emptive immunotherapy? J Clin Oncol 2004;22(9):1696-705.

35. Khan F, Agarwal A, Agarwal S. Significance of chimerism in hematopoietic stem cell transplantation: new variations on an old theme. Bone Marrow Transplant 2004;34:1-12.

NOT FOR CLINICAL USE

3. OBJECTIVES

3.1. *Primary*

1. Evaluate Progression Free Survival (PFS) for all UK patients, stratified by risk groups. PFS is defined throughout as the time from trial entry to the first occurrence of progression, relapse, death in CCR or second malignancy.
2. Evaluate whether a Minimal Residual Disease (MRD) level of 10^{-4} is a suitable criterion at the end of induction, on which to decide whether chemotherapy or stem cell transplantation (SCT) will be most beneficial to patients in the intermediate risk group.

3.2. *Secondary*

Use of MRD as a surrogate marker for response to therapy.

4. STUDY POPULATION

4.1. *Number of Subjects*

A total of ~350 patients will be recruited for the study. For the UK, it is expected that approximately 50 patients will be recruited per year. International recruitment commenced August 2006 and should yield around 60 patients per year [that is 30 patients from the Australia/New Zealand Group and 30 patients from The Netherlands Group].

5. ELIGIBILITY CRITERIA

5.1. *Inclusion Criteria*

A subject will be eligible for inclusion in this study only if all of the following criteria apply:

1. All patients aged 1-18 years who have been previously diagnosed to have acute lymphoblastic leukaemia and have either relapsed after treatment or have primary refractory disease
2. Only those patients in whom this is the first relapse are eligible
3. Written, informed consent according to national guidelines
4. Appropriate ethical committee approval.

5.2. *Exclusion Criteria*

A subject will not be eligible for inclusion in this study if any of the following criteria apply:

1. Those who have first relapse but have already received chemotherapy or radiotherapy for the relapse, prior to starting R3
2. Patients who have had a prior bone marrow transplant
3. Those with mature B-cell ALL

NOT FOR CLINICAL USE

6. ASSESSMENT PRIOR TO STARTING TREATMENT

Centres treating patients with leukaemia are expected to have appropriate investigation protocols for the pre-treatment assessment of new patients, for monitoring progress during treatment and once therapy has ended, and for dealing with any complications that may arise. For trial purposes, in addition to a blood count, mandatory investigations at the time of diagnosis include:

A bone marrow aspirate with material being sent for:

i. morphology

ii. immunophenotyping

iii. chromosome analysis and ploidy status

iv. baseline bone marrow sample for PCR- Based MRD analyses (see Appendix 2)

v. additional bone marrow sample for storage

The above are essential for the trial. If any of these tests have been omitted on the first diagnostic sample, a second sample must be taken before treatment starts, unless prevented by clinical circumstances. A trephine biopsy is required if an adequate sample cannot be obtained by aspiration. These biological studies are vital to this trial. If possible, please do not treat until the MRD coordinating centre has verified that a requisite number of cells have been received.

Other assessments/samples requested:

a. Lumbar puncture for CSF cytology and additional CSF sample for storage

b.. Echocardiogram for LV function

7. DEFINITIONS OF MARROW STATUS AND RESPONSE

BM1:	<5% blasts, regardless of the number of lymphocytes present.
Hypocellular BM1	<5% blasts in a hypocellular marrow.
BM2:	5-25% blasts, regardless of the number of mature lymphocytes present.
BM3:	>25% blasts

NOT FOR CLINICAL USE

8. CHEMOTHERAPY SCHEDULES

NOT FOR CLINICAL USE

INDUCTION

Phase I

ALL R3

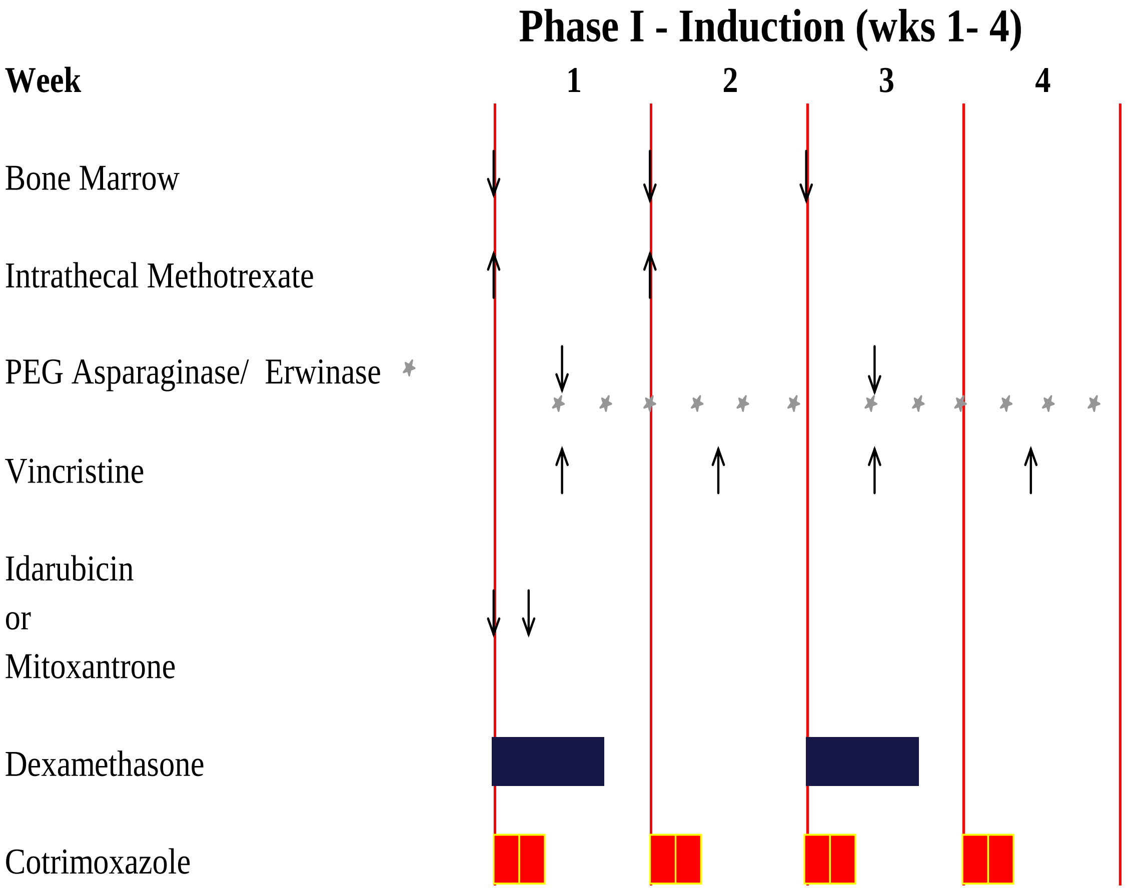

Intrathecal Methotrexate on d1, wk1 and d1, wk2. <2yrs 8mg; 2 yrs 10 mg; ≥3 yrs 12 mg

Idarubicin or Mitoxantrone on d 1 and 2, wk1. 10mg/m^2 iv infusion over 1 hour

Dexamethasone on d 1-5, wk1 and d 1-5, wk3. 20 mg/m^2 orally in 2 divided doses per day (max 40mg/day)

Vincristine on d3, Wk 1, d3 of wk2, d3 of wk 3, d3 of wk4. 1.5mg/m^2 iv bolus **MAX 2mg as a single dose**

PEG Asparaginase on d 3, wk 1and d3, wk3. 1000 u/m^2 im

OR

*Erwinase 20,000 units/m^2 IM on **day 3 of week 1** and **then alternate days for 12 doses in total**

Cotrimoxazole twice daily on two consecutive days from d 1 of weeks 1-4

Note this flow diagram is only provided as a guide.
It is mandatory to read the corresponding pages of the protocol for a more detailed description

8.1. *Remission Induction : Phase I : Weeks 1-4*

This phase runs for 28 days from day 1 (week 1) to day 28 (week 4) (i.e. 4 weeks).

a) **Fluids** All patients should be adequately hydrated (at least 2-2.5 l/m²/24hrs given parenterally for the first 48 hours).

b) **Allopurinol** 100 mg/m² oral three times daily, should start 24 hours before chemotherapy and continue for 5 days.

c) **Dexamethasone** Patients will receive dexamethasone 20mg/m²/day orally for 5 days, on days 1 - 5, week 1 and then again on days 1 - 5, week 3. The steroid should be divided into two doses per day with a maximum daily dose of 40mg.

d) **Vincristine** 1.5 mg/m² **(maximum single dose 2 mg)** IV bolus weekly on d3 of week 1, d3 of week 2, d 3 of week 3, and d3 of week 4.

e) **PEG-Asparaginase** 1000 u/m² IM on d 3 of week 1 and d3 of week 3.

OR

Erwinase Erwinase 20,000 units/m² IM on d 3 of week 1 and then alternate days for 12 doses in total. (That is replacing each dose of PEG with 6 doses of Erwinase)

f) **Intrathecal Methotrexate**

On d1 of week 1 and d1 of week 2.

Dose by age:

<2yrs: 8mg

2 years: 10mg

≥ 3yrs: 12 mg

Patients who have CNS disease at presentation should receive weekly doses until two clear CSF samples are obtained

g) **Idarubicin** 10 mg/m², intravenously over 1 hour on d1 and 2 of week 1

OR

Mitoxantrone 10 mg/m², intravenously over 1 hour on d1 and 2 of week 1

h) **Co-trimoxazole** given twice daily on two consecutive days each week **from weeks 1-4,** maintaining the longest possible

NOT FOR CLINICAL USE

interval from intrathecal methotrexate in order to avoid drug interactions.

**Surface area**	**Co-trimoxazole**	**Trimethoprim**	**Sulphamethoxazole**
0.5-0.75m^2	240 mg bd	40 mg bd	200 mg bd
0.76-1.0m^2	360 mg bd	60 mg bd	300 mg bd
Over 1.0m^2	480 mg bd	80 mg bd	400 mg bd

Notes:

(1) For Permitted modifications during Induction see Appendix 11.

(2) The high dose of dexamethasone during this 4 week period may cause hyperglycemia. It is easier to control this if hydration fluids are free of dextrose.

(3) There is a high morbidity when inducing children with relapsed disease. It is advisable to keep these children in hospital during the induction period and they will require proactive nutritional support.

(4) Please arrange to send an additional sample of diagnostic marrow, peripheral blood, cerebrospinal fluid and tissue (if obtained) for storage. Day 1 Week 2 and Day 1 Week 3 samples are to be sent to the MRD laboratory only.

	Sample site			
Timepoints	Bone marrow	CSF	Tissue	Peripheral Blood
Diagnosis	MRD + FLOW + Storage + Asparaginase	Storage	Storage	Storage + Asparaginase
Day 8	MRD + FLOW			
Day 10				Asparaginase
Day 15	MRD + FLOW			
Day 24				Asparaginase
Day 35	MRD + FLOW + Storage	Storage	Storage	Storage

Send [in order of Priority] :
MRD to local MRD laboratory
Asparaginase to CIGMR (Manchester)
FLOW to Birmingham
Storage to CIGMR (Manchester)
Full address details available Appendix 8

CLINICAL USE

CONSOLIDATION

Phase II

NOT FOR CLINICAL USE

ALL R3 Phase II - Consolidation (wks 5 - 8)

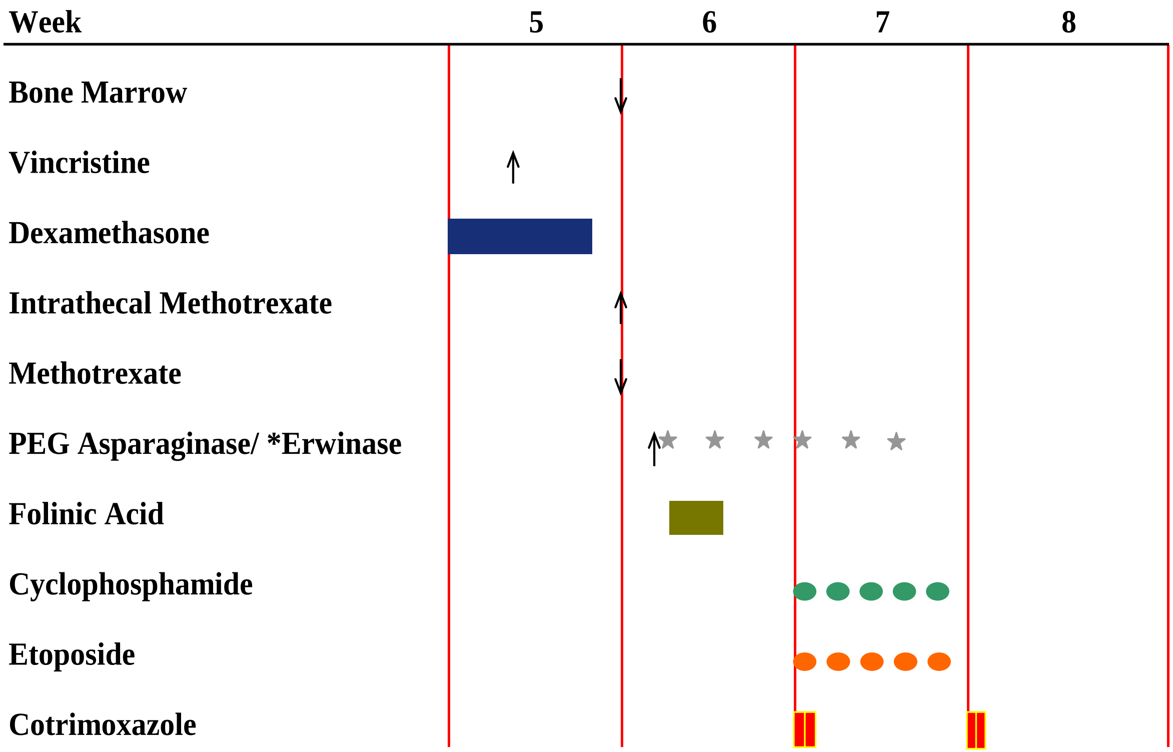

Dexamethasone, d1-5, Wk 5. 6 mg/m^2 orally in 2 divided doses

Vincristine on d3, Wk 5. 1.5mg/m^2 iv bolus **MAX 2mg as a single dose**

Proceed to week 6 only when count is recovering and ANC $\geq 0.5 \times 10^9$/l and platelets $\geq 50 \times 10^9$/l,

Perform Bone marrow examination at the beginnning of week 6 (d35)

Intrathecal Methotrexate on d1, wk6. <2yrs 8mg; 2 yrs 10 mg; ≥3 yrs 12 mg

Methotrexate, d1, Wk6. 1000 mg/m^2 iv infusion over 36 hours

PEG Asparaginase, d 2 Wk 6, 4 hours **after the end** of the methotrexate infusion. 1000 u/m^2 im

OR if allergic E. Coli Asparaginase

Erwinase 20,000 units/m^2 IM on day 2 of week 6, 4 hrs after end of MTX infusion

and then alternate days for 6 doses in total

Folinic Acid 48 hrs **after the beginning** of methotrexate infusion. 15 mg/m^2 iv bolus at 48 and 54 hours

Proceed to week 7 only when count is recovering and ANC $\geq 0.5 \times 10^9$/l and platelets $\geq 50 \times 10^9$/l,

Cyclophosphamide, d1-5, Wk 7. 440 mg/m^2 iv infusion over 30 minutes

Etoposide, d1-5 Wk 7. 100 mg/m^2 iv infusion over 4 hours

Cotrimoxazole twice daily on two consecutive days Wk 7, 8

No Cotrimoxazole, the week prior to and the week of iv methotrexate

Note this flow diagram is only provided as a guide.
It is mandatory to read the corresponding pages of the protocol for a more detailed description

8.2. *Consolidation : Phase II : Weeks 5 – 8*

This phase of intensification starts as soon as the child is able to tolerate it. The day Methotrexate is given will be counted as the beginning of week 6.

Week 5

a) **Vincristine** 1.5 mg/m^2 **(maximum single dose 2 mg)** IV on day 3 of week 5

b) **Dexamethasone** 6 mg/m^2 orally for 5 days, d1-5 of week 5, in two divided doses.

Please prescribe Dexamethasone and Vincristine if the count has not yet recovered but the child is well.

Do not use Co-trimoxazole during this week.

Proceed to week 6, only when marrow is recovering and ANC >0.5 x 10^9/l and platelets >50 x 10^9/l

Week 6

Bone marrow aspirate for MRD. If a patient has M3 marrow at day 35 they may be removed from the protocol and considered to be refractory.

a) **Intrathecal Methotrexate** On day 1, week 6

Dose by age:

<2yrs: 8mg

2 yrs: 10mg

≥3yrs: 12 mg.

This is ideally given prior to starting the IV Methotrexate infusion. If it needs to be given during the infusion, on no account should the infusion be stopped.

b) **Methotrexate** On day 1, week 6, 1000 mg/m^2 IV. 10% of this will be given intravenously as a bolus and the remaining 90% as a continuous infusion on d 1 for 36 hours with concomitant hydration (see Appendix 3).

c) **PEG-Asparaginase** 1000 u/m^2 IM on d 2, week 6 [4 hours after the end of the Methotrexate infusion]

OR (if allergic to E Coli Asparaginase)

NOT FOR CLINICAL USE

Erwinase Erwinase 20,000 u/m² IM on d 2 of week 6 [4 hrs after end of MTX infusion] and then Mon/Wed/ Friday for 6 doses in total [this will overlap into Week 7]

d) **Folinic acid** 15 mg/m² per dose intravenously. Starts 48 hours after the beginning of the methotrexate infusion, and given at 48 and 54 hours (see Appendix 3).

Do not use Cotrimoxazole during this week.

Consolidation Weeks 7– 8
Commence this phase as long as the child is well and ANC >0.5 x 10^9/l and platelets >50 x 10^9/l and counts are recovering

Chemotherapy should be continued in a well child with fever of unknown origin but no neutropenia. Any **serious** infection, such as varicella, Pneumocystis pneumonia, or neutropenia with fever, and presumed or proven infection, warrants chemotherapy interruption at any time.

a) **Cotrimoxazole** given twice daily on two consecutive days in week **7 and week 8**

Surface area	Co-trimoxazole	Trimethoprim	Sulphamethoxazole
0.5-0.75m²	240 mg bd	40 mg bd	200 mg bd
0.76-1.0m²	360 mg bd	60 mg bd	300 mg bd
over 1.0m²	480 mg bd	80 mg bd	400 mg bd

b) **Etoposide** 100 mg / m² IV infused over 4 hours on d1, 2, 3, 4 and 5 of week 7

c) **Cyclophosphamide** 440 mg / m² IV infused over 30 minutes on d1, 2, 3, 4 and 5 of week 7. Maintain fluids at 2-x maintenance for at least 4 hours after the dose. Use frusemide 0.25 - 0.5 mg/kg IV for urine output <3ml/kg/hr after cyclophosphamide. Mesna is not required unless there is microscopic haematuria or past history of gross haematuria.

Day 35 marrow sample to be sent to the MRD laboratory. Please arrange to send an additional sample of marrow, peripheral blood and cerebrospinal fluid for storage.

NOT FOR CLINICAL USE

	Sample site			
Timepoint	Bone marrow	CSF	Tissue	Peripheral Blood
Day 42 (Wk7, Day 5)				Asparaginase

Send [in order of priority] :
MRD to local MRD laboratory
Asparaginase to CIGMR (Manchester)
FLOW to Birmingham
Storage to CIGMR (Manchester)
Full address details available Appendix 8.

NOT FOR CLINICAL USE

INTENSIFICATION

Phase III

NOT FOR CLINICAL USE

ALL R3   Phase III - Intensification (wks 9 - 13)

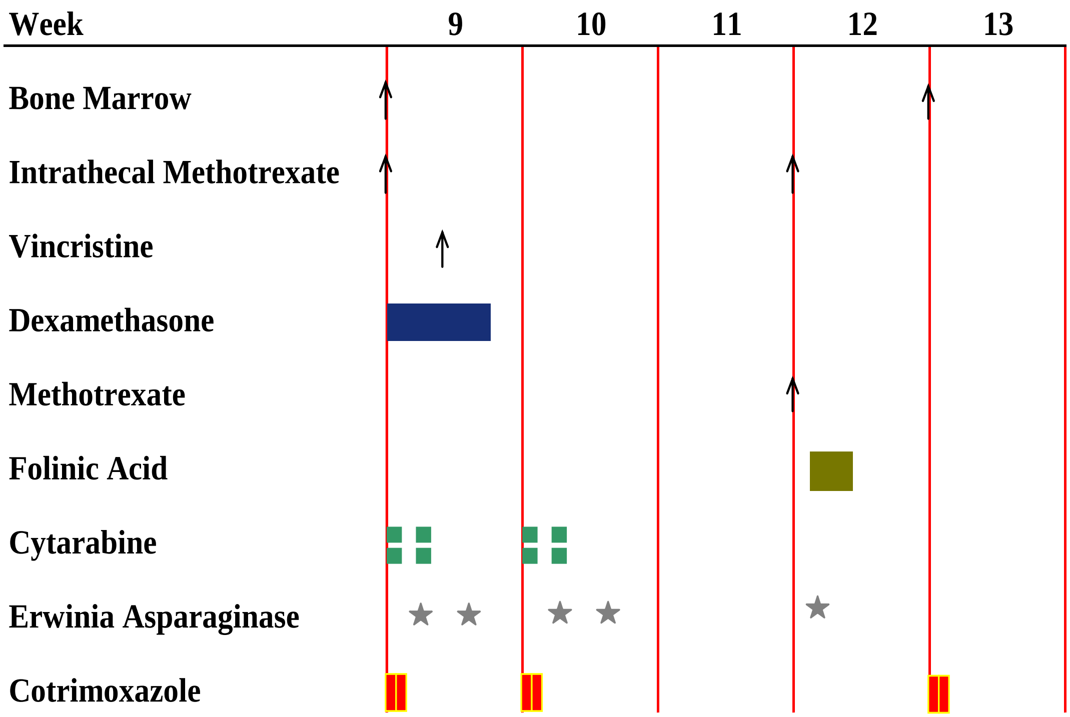

Start Phase III when marrow is recovering and ANC ≥0.5 and platelets ≥50
(It is acceptable to give Vincristine and steroids if there is a delay)

Dexamethasone, d1-5, Wk 9. 6 mg/m^2 orally in 2 divided doses
Vincristine on d3, Wk 9. 1.5mg/m^2 iv bolus MAX 2mg as a single dose
Intrathecal Methotrexate on d1, wk 9. <2yrs 8mg; 2yrs 10 mg; ≥3 yrs 12 mg
Cytarabine 3000 mg/m^2 as iv infusion over 3 hours, every 12 hours, d1 and 2 Wk9 and d1, 2 Wk10
Erwinase 20,000 units/m^2 IM on d 2 and d 4 of Wk 9 and on d 2 and d 4 of Wk 10
Cotrimoxazole twice daily on two consecutive days Wks 9-10 , 13
Prednisolone eye drops every 2 hours from d1, wk9 and stopped 5 days after the last cytarabine infusion

Start **Week 12** when marrow is recovering and ANC ≥0.5 and platelets ≥50
No Cotrimoxazole, the week prior to and the week of iv methotrexate

Intrathecal Methotrexate on d1 wk12. <2yrs 8mg; 2yrs 10 mg; ≥3 yrs 12 mg
Methotrexate, d1 Wk12. 1000 mg/m^2 iv infusion over 36 hours
Folinic Acid 48 hrs after the beginning of methotrexate infusion. 15 mg/m^2 iv bolus at 48 and 54 hours
Erwinase 20,000 units/m^2 IM on d 2 of Wk 12, 4hrs after the end of the methotrexate infusion

Note this flow diagram is only provided as a guide.
It is mandatory to read the corresponding pages of the protocol for a more detailed description

8.3. *Intensification : Phase III : Weeks 9-13*

This phase starts as soon as the child is able to tolerate it. The ANC should be > 0.5 x 10^9/l and the platelets > 50 x 10^9/l, with evidence of count recovery. Vincristine and Dexamethasone may be given if the counts have not recovered.

The day cytarabine is first given will be counted as the beginning of week 9.

Week 9 and 10

Bone marrow aspirate for MRD at week 9

a) **Vincristine** — 1.5 mg/m^2 **(maximum single dose 2 mg)** IV on day 3 of week 9

b) **Dexamethasone** — 6 mg/m^2 orally for 5 days, d1-5 of week 9, in two divided doses.

c) **Intrathecal Methotrexate** — On day 1 of week 9.

Dose by age:

<2yrs: 8mg

2 yrs: 10mg

≥ 3yrs: 12 mg.

d) **Cytarabine** — 3000 mg/m^2 to be infused every 12 hrs via 3 hour IV infusions. Given on d1 and d2 week 9, d1 and d2 week 10 (total of 8 doses)

e) **Erwinase** — Erwinase 20,000 units/m^2 IM on day 2 and day 4 week 9 and on day 2 and day 4 week 10 [given 4 hrs after last cytarabine infusion]

f) **Cotrimoxazole** — given twice daily on two consecutive days per week, week 9, week 10 and week 13.

Surface area	Co-trimoxazole	Trimethoprim	Sulphamethoxazole
0.5-0.75m^2	240 mg bd	40 mg bd	200 mg bd
0.76-1.0m^2	360 mg bd	60 mg bd	300 mg bd
over 1.0m^2	480 mg bd	80 mg bd	400 mg bd

Patients should be prescribed **prednisolone eye-drops** 2 hourly from day 1, Week 9 until 5 days after the last dose of cytarabine.

Week 11

Stop Cotrimoxazole

NOT FOR CLINICAL USE

Week 12

This phase of intensification starts as soon as the child is able to tolerate it. The ANC should be > 0.5 x 10^9/l and the platelets > 50 x 10^9/l, with evidence of count recovery and the child should be clinically well.

a) **Intrathecal Methotrexate** On day 1, week 12.
Dose by age:
<2yrs: 8mg
2 yrs: 10mg
≥ 3yrs: 12 mg

This is ideally given prior to starting the IV methotrexate infusion. If it needs to be given during the infusion, on no account should the infusion be stopped.

b) **Methotrexate** On day 1, week 12, 1000 mg/m² IV. 10% of this will be given as an IV bolus and the remaining 90% as a continuous infusion from d 1 for 36 hours, with concomitant hydration (see Appendix 3).

c) **Folinic acid** 15 mg/m² per dose intravenously. Starts 48 hours after the beginning of the infusion, and given at 48 and 54 hours (see Appendix 3).

d) **Erwinase** Erwinase 20,000 units/m2 IM on day 2 week 12, [4 hours post Methotrexate]

Week 13 marrow sample to be sent to the MRD laboratory. Please arrange to send an additional sample of marrow, peripheral blood and cerebrospinal fluid for storage.

	Sample site			
Timepoints	Bone marrow	CSF	Tissue	Peripheral Blood
Week 13	MRD + FLOW + Storage	Storage	Storage	Storage
Day 1 Wk12				Asparaginase

**Send [in order of priority]:**
MRD to local MRD laboratory
Asparaginase to CIGMR (Manchester)
FLOW to Birmingham
Storage to CIGMR (Manchester)
Full address details available Appendix 8.

NOT FOR CLINICAL USE

NOT FOR CLINICAL USE

FLAD

Phase IV

ALL R3 Phase IV - FLAD

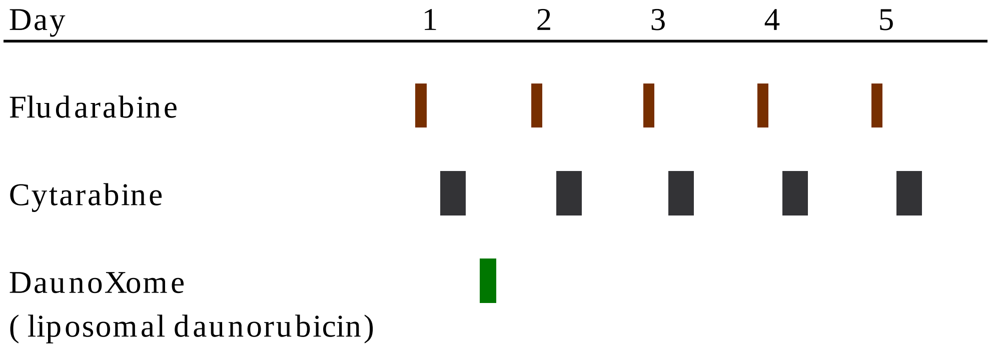

Fludarabine d 1-5. 25mg/ m^2 daily, as a 30 minute iv infusion
Cytarabine d1-5. 2000mg/ m^2 daily, as a 4-hour iv infusion
DaunoXome(liposomal daunorubicin) d1. 100 mg/ m^2 as a 2-hour iv infusion

Start Cytarabine 4 hours after the start of the Fludarabine infusion and DaunoXome starting 4 hours after the start of the Cytarabine infusion

G-CSF 5 micrograms/ kg daily from day 7 until count recovery either IV or subcut

Prednisolone eye drops every 2 hours from days 1-10. This course gives 5 days post Cytarabine, intensification is only for 48 hrs post Cytarabine

Note, only those who are MRD positive > 10^{-3} prior to BMT are eligible

Note this flow diagram is only provided as a guide.
It is mandatory to read the corresponding pages of the protocol for a more detailed description

NOT F

8.4. *Pre SCT Cytoreduction – Phase IV FLAD*

NB. Only those patients who are MRD $\geq 10^{-3}$ at week 13 are eligible for this phase of treatment.

This phase of intensification starts as soon as the child is able to tolerate it. The ANC should be $\geq 0.5 \times 10^9/l$ and the platelets $\geq 50 \times 10^9/l$, with evidence of count recovery and the child should be clinically well.

a) **Fludarabine** 25mg/m² daily, as a 30 minute IV infusion, 4 hours prior to Cytarabine, on days 1-5.

b) **Cytarabine** 2000mg/m² daily, as a 4 hour infusion, over 4 hours. Starting 4 hours after the start of the Fludarabine infusion, on days 1-5.

c) **DaunoXome** 100mg/m² as a 2 hour infusion on day 1, starting 4 hours after the start of the Cytarabine infusion.

G-CSF 5mcg/kg daily from day+7 until count recovery. Subcutaneous or IV depending on inpatient or outpatient status.

Patients should be prescribed prednisolone eye-drops 2 hourly on days 1-10.

NB. The sequence of chemotherapy is important. Fludarabine should precede Cytarabine by 4 hours, which in turn precedes DaunoXome by a further 4 hours.

NOT FOR CLINICAL USE

NOT FOR CLINICAL USE

INTERIM MAINTENANCE

Phase V

ALL R3

Phase V - Interim Maintenance - Cycle 1

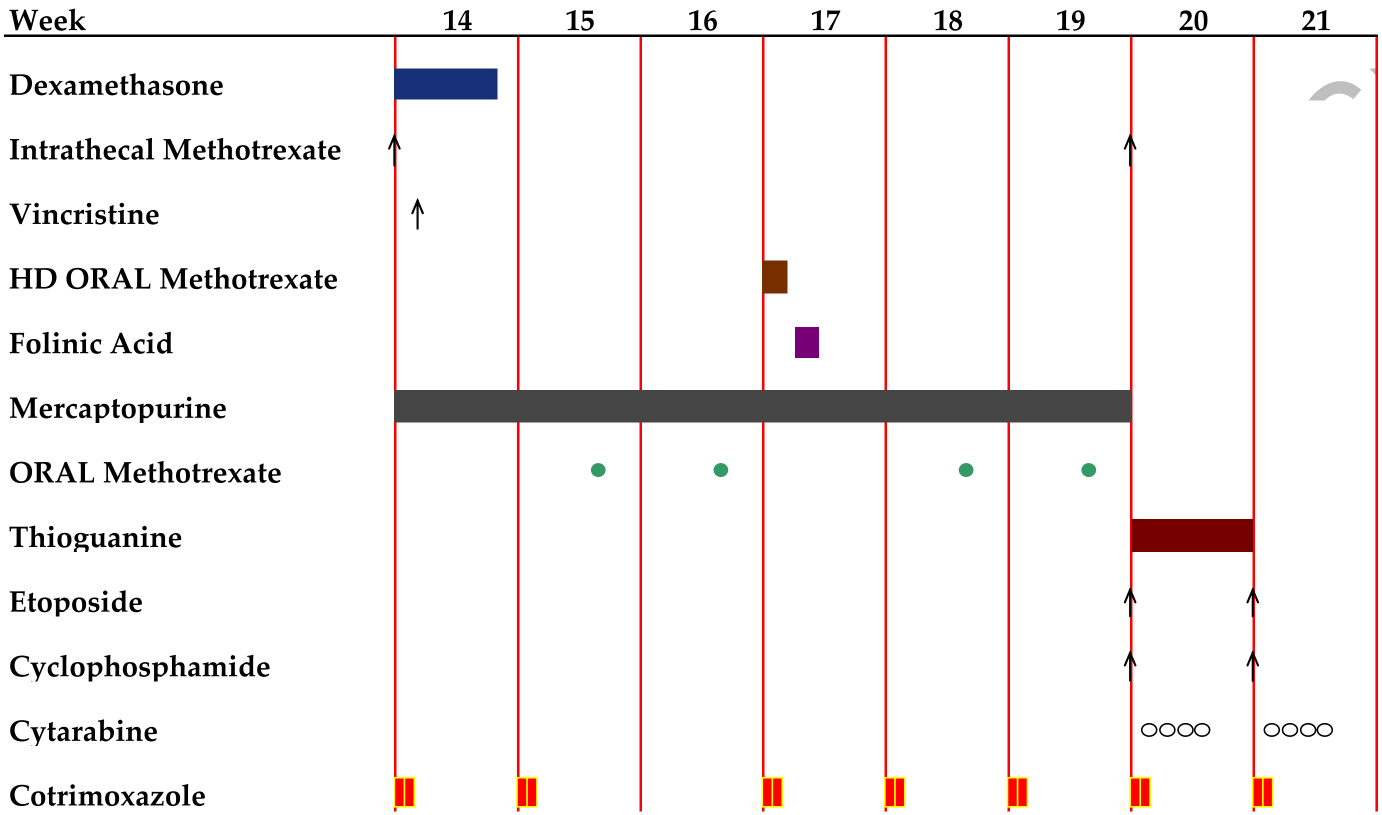

Note this flow diagram is only provided as a guide.

It is mandatory to read the corresponding pages of the protocol for a more detailed description

Dexamethasone, d1-5, wk14. 6 mg/m^2 orally in 2 divided doses orally
Intrathecal Methotrexate on d1, wks 14, 20. <2yrs 8mg; 2 yrs 10 mg; ≥3 yrs 12 mg
Vincristine on d3, wk14. 1.5mg/m^2 iv bolus **MAX 2.0 mg as a single dose**
HD ORAL Methotrexate d1, wk 17. 25 mg/m^2 every 6 hours for 4 doses orally
Folinic Acid, d3, wk17. 10 mg/m^2 every 6 hours for 2 doses orally
Mercaptopurine daily, wk 14-19. 75 mg/m^2/day orally

ORAL Methotrexate once weekly, wk 15, 16 and 18, 19. 20 mg/m^2 orally
Thioguanine d1-7, wk 20, . 40mg/m^2/day orally
Etoposide, d1 wk20 and d1 wk21. 150 mg/m^2 iv infusion over 4 hours
Cyclophosphamide, d1, wk20 and d1, wk21. 300 mg/m^2 iv infusion over 30 mts
Cytarabine d2-5 wk20, d2-5, wk 21. 50 mg/m^2 iv/sc per dose for 8 doses
Cotrimoxazole twice daily on two consecutive days Wks 14-15, 17-21(give the Cotrimoxazole on wk17 after the Folinic Acid)

Note, children who have received cranial radiotherapy in R3 do not receive intrathecal methotrexate during this phase (see alternative flowsheet)

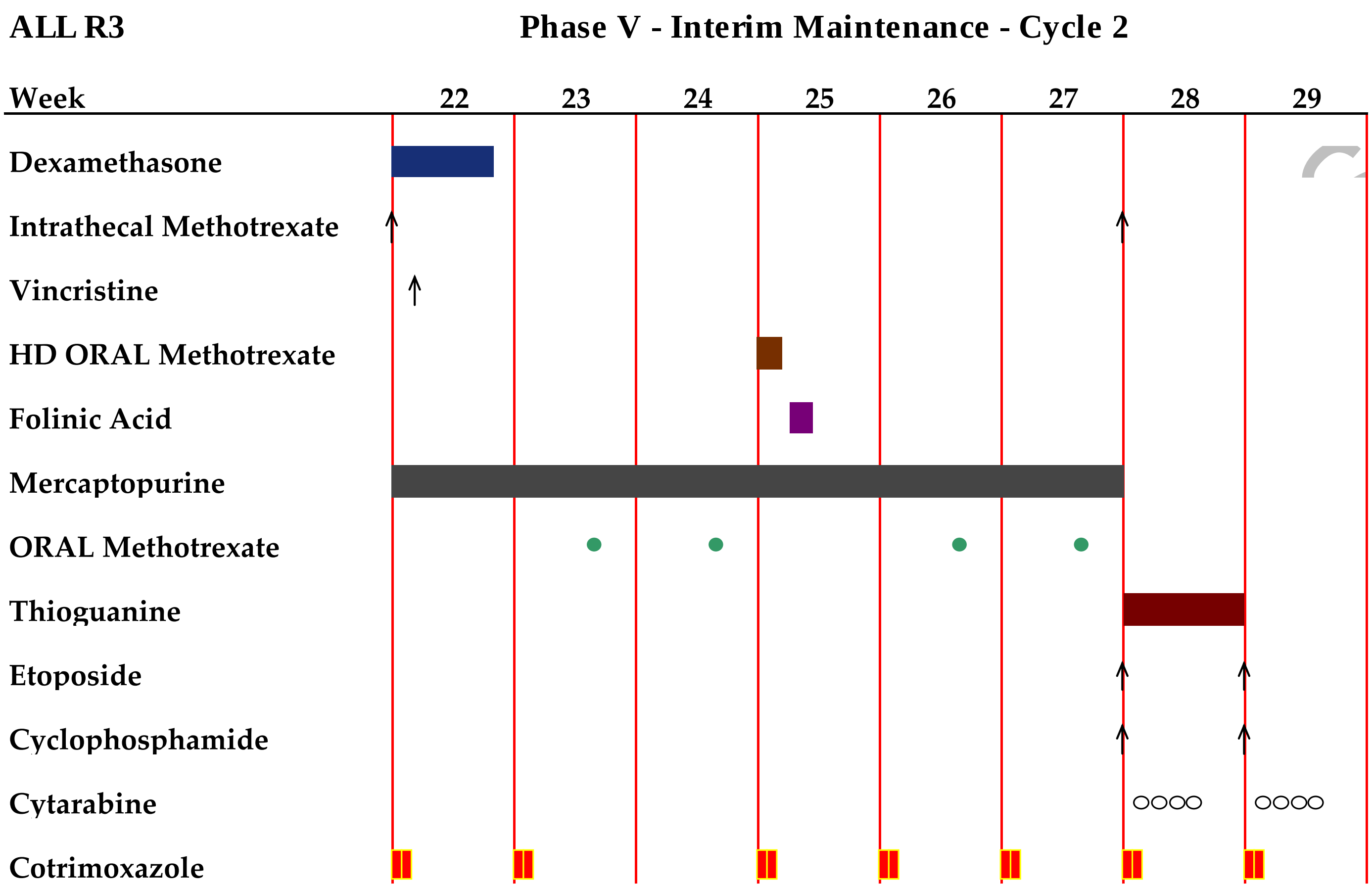

Note this flow diagram is only provided as a guide.

It is mandatory to read the corresponding pages of the protocol for a more detailed description

Dexamethasone, d1-5, wk 22 6 mg/m^2 orally in 2 divided doses orally
Intrathecal Methotrexate on d1, wks 22, 20. <2yrs 8mg; 2 yrs 10 mg; ≥3 yrs 12 mg
Vincristine on d3, wk22. 1.5mg/m^2 iv bolus **MAX 2.0 mg as a single dose**
HD ORAL Methotrexate d1, wk 25. 25 mg/m^2 every 6 hours for 4 doses orally
Folinic Acid, d3, wk25. 10 mg/m^2 every 6 hours for 2 doses orally
Mercaptopurine daily, wk 22-27. 75 mg/m^2/day orally

ORAL Methotrexate once weekly, wk 23, 24 and 26, 27. 20 mg/m^2 orally
Thioguanine d1-7, wk 28. 40mg/m^2/day orally
Etoposide, d1 wk28 and d1 wk29. 150 mg/m^2 iv infusion over 4 hours
Cyclophosphamide, d1, wk28 and d1, wk29. 300 mg/m^2 iv infusion over 30 mts
Cytarabine d2-5 wk28, d2-5, wk 29. 50 mg/m^2 iv/sc per dose for 8 doses
Cotrimoxazole twice daily on two consecutive days Wks 22-23, 25-26 (give the Cotrimoxazole on wk25 after the Folinic Acid)

Note, children who have received cranial radiotherapy in R3 do not receive intrathecal methotrexate during this phase (see alternative flowsheet)

ALL R3 Phase V - Interim Maintenance - Cycle 1 for those receiving Cranial XRT

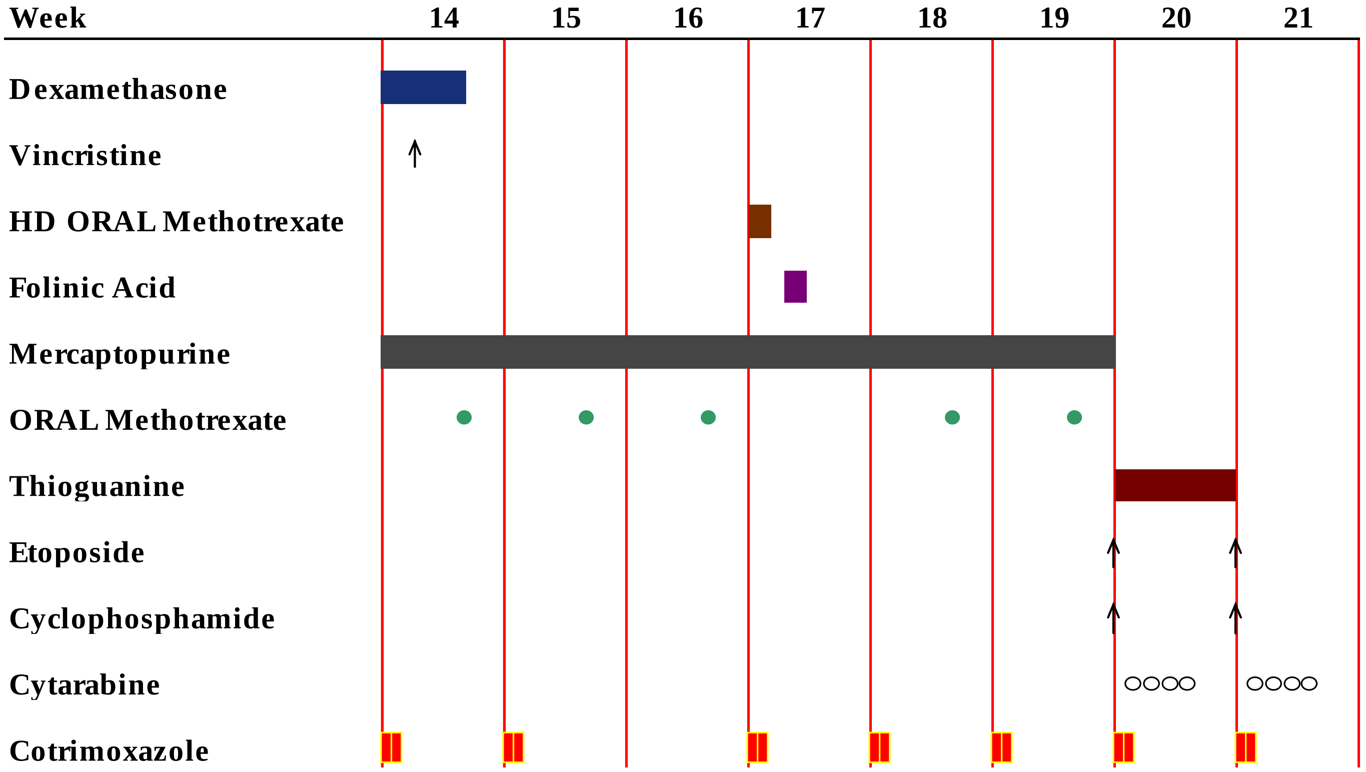

Note this flow diagram is only provided as a guide.

It is mandatory to read the corresponding pages of the protocol for a more detailed description

Dexamethasone, d1-5, wk14. 6 mg/m^2 orally in 2 divided doses orally
Vincristine on d3, wk14. 1.5mg/m^2 iv bolus **MAX 2.0 mg as a single dose**
HD ORAL Methotrexate d1, wk 17. 25 mg/m^2 every 6 hours for 4 doses orally
Folinic Acid, d3, wk17. 10 mg/m^2 every 6 hours for 2 doses orally
Mercaptopurine daily, wk 14-19. 75 mg/m^2/day orally
ORAL Methotrexate once weekly, wk 15, 16 and 18, 19. 20 mg/m^2 orally

Thioguanine d1-7, wk 20, . 40mg/m^2/day orally
Etoposide, d1 wk20 and d1 wk21. 150 mg/m^2 iv infusion over 4 hours
Cyclophosphamide, d1, wk20 and d1, wk21. 300 mg/m^2 iv infusion over 30 mts
Cytarabine d2-5 wk20, d2-5, wk 21. 50 mg/m^2 iv/sc per dose for 8 doses
Cotrimoxazole twice daily on two consecutive days Wks 14-15, 17-21(give the Cotrimoxazole on wk17 after the Folinic Acid)

Note: Do not use this phase concurrent with cranial XRT.
Children who have received cranial radiotherapy in R3 do not receive intrathecal methotrexate during this phase

ALL R3 Phase V - Interim Maintenance - Cycle 2 for those receiving Cranial XRT

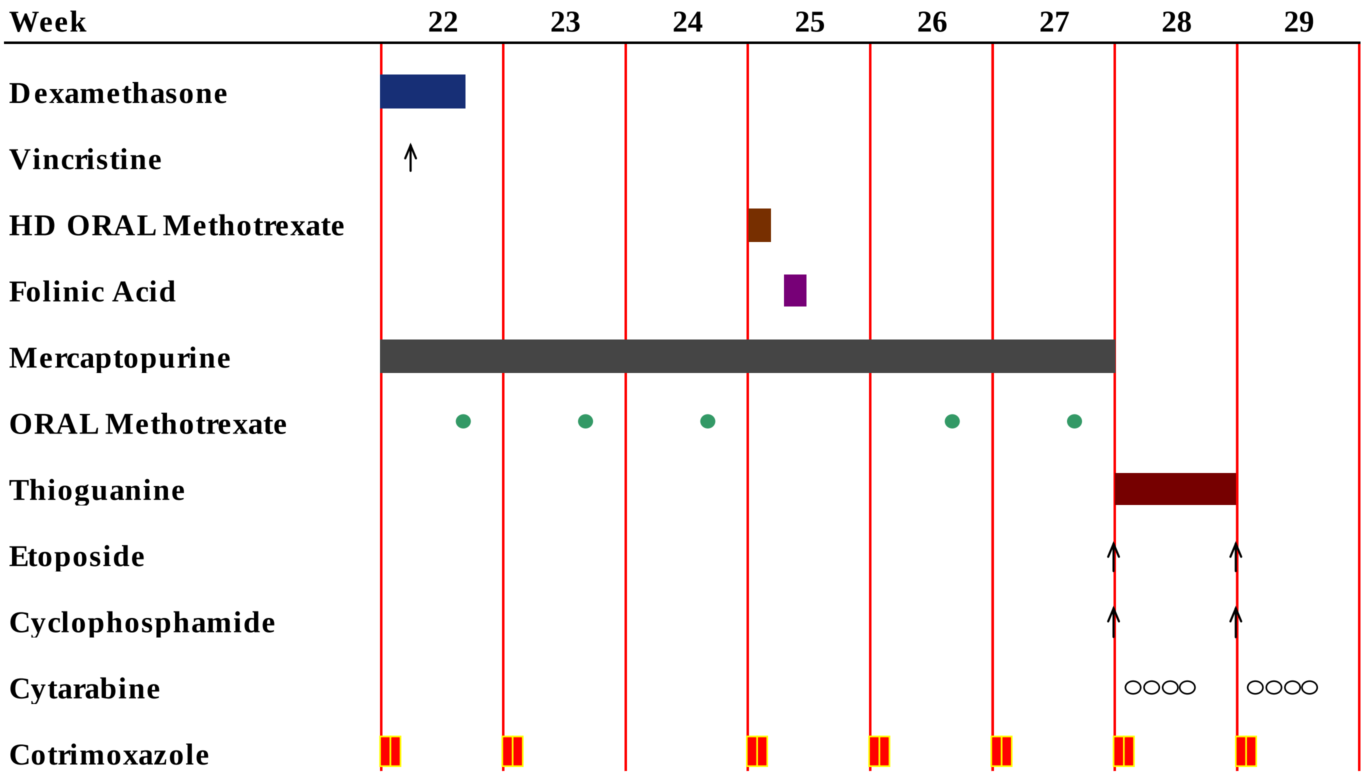

Note this flow diagram is only provided as a guide.

It is mandatory to read the corresponding pages of the protocol for a more detailed description

Dexamethasone, d1-5, wk 22. 6 mg/m^2 orally in 2 divided doses orally
Vincristine on d3, wk22. 1.5mg/m^2 iv bolus **MAX 2.0 mg as a single dose**
HD ORAL Methotrexate d1, wk 25. 25 mg/m^2 every 6 hours for 4 doses orally
Folinic Acid, d3, wk25. 10 mg/m^2 every 6 hours for 2 doses orally
Mercaptopurine daily, wk 22-27. 75 mg/m^2/day orally
ORAL Methotrexate once weekly, wk 23, 24 and 26, 27. 20 mg/m^2 orally

Thioguanine d1-7, wk 28. 40mg/m^2/day orally
Etoposide, d1 wk28 and d1 wk29. 150 mg/m^2 iv infusion over 4 hours
Cyclophosphamide, d1, wk28 and d1, wk29. 300 mg/m^2 iv infusion over 30 mts
Cytarabine d2-5 wk28, d2-5, wk 29. 50 mg/m^2 iv/sc per dose for 8 doses
Cotrimoxazole twice daily on two consecutive days Wks 22-23, 25-26 (give the Cotrimoxazole on wk25 after the Folinic Acid)

Note: Do not use this phase concurrent with cranial XRT.
Children who have received cranial radiotherapy in R3 do not receive intrathecal methotrexate during this phase

8.5. *Interim Maintenance : Phase V : Weeks 14-29*

Phase V is primarily for those who will not be transplanted. If there is a delay in those receiving a bone marrow transplant but not receiving FLAD, it is recommended that they proceed to Phase VI (maintenance) to avoid toxicity.

Children not being transplanted will receive a total of 104 weeks of continuous therapy from the start of phase V (week 14).

Those receiving cranial irradiation should receive this prior to starting this phase of treatment. Those receiving testicular irradiation will receive interim maintenance concurrently.

Phase V consists of 2 blocks of treatment which lasts for 56 days each.

a) Vincristine 1.5 mg/m^2 **(maximum single dose 2 mg)** IV on day 3 of week 14 and day 3 of week 22

b) Dexamethasone 6 mg/m^2 for 5 days, d1-5 of week 14 and day 1-5 week 22, in two divided doses

c) Intrathecal Methotrexate On d1, of week 14, week 20, week 22 and week 28.
Dose by age: <2yrs: 8mg
2 yrs: 10mg
> 3yrs: 12 mg

d) ORAL Methotrexate 20 mg/m^2 orally once weekly on weeks 15,16,18,19, 23,24,26,27. Should be taken as a single dose. Dose adjustments are described in Appendix 5.

e) HD ORAL Methotrexate (High dose) 100 mg/m^2 orally given as 25 mg/m^2 every 6 hours for four doses on day 1 of week 17 and day 1 week 25. To obtain maximum absorption of high dose oral Methotrexate the dose must be split, as per protocol. To avoid waking patients intervals can be stretched to 8 hours overnight.

f) Folinic acid 10 mg/m^2 orally for 2 doses 6 hours apart, first dose on d3 of week 17 and d3 week 25. [48 hours after first dose of methotrexate at 25mg/m^2]

g) Mercaptopurine 75 mg/m^2 orally, daily from weeks 14 to 19, and weeks 22-27. Doses should be taken at least one hour after the evening meal without milk products. Dose adjustments are described in Appendix 5.

h) Thioguanine 40 mg/m^2 orally each day from d1 – 7, week 20 and d1 – 7 week 28. Doses should be taken at least one hour after the evening meal without milk products.

i) Etoposide 150 mg/m^2 IV infused over 4 hours on d1, weeks 20 and 21 and day 1 weeks 28 and 29.

NOT FOR CLINICAL USE

j) Cyclophosphamide 300 mg/m^2 IV infused over 30 minutes on d1 of weeks 20 and 21 and d1 of weeks 28 and 29. Maintain fluids at 2-x maintenance for at least 4 hours after the dose. Use frusemide 0.25 - 0.5 mg/kg IV for urine output <3ml/kg/hr after cyclophosphamide. Mesna is not required unless there is microscopic haematuria or past history of gross haematuria.

g) Cytarabine 50mg/m^2/day by IV push or subcutaneously – 8 doses in two pulses of 4 days each; d2-5 of weeks 20,21 and d2-5 of weeks 28, 29

h) Cotrimoxazole given twice daily on two consecutive days per week, weeks 14, 15, 17 – 21, 22, 23 and weeks 25-29. Omit Cotrimoxazole on week 16 and 24 and give the Cotrimoxazole on weeks 17 and 25 after Folinic Acid.

Surface area	Co-trimoxazole	Trimethoprim	Sulphamethoxazole
0.5-0.75m^2	240 mg bd	40 mg bd	200 mg bd
0.76-1.0m^2	360 mg bd	60 mg bd	300 mg bd
over 1.0m^2	480 mg bd	80 mg bd	400 mg bd

Please note, the cycle of therapy requires that the clock must stop at the end of weeks 19 and 27 if the ANC is < 0.5 x 10^9/l or the platelet count is < 50 x 10^9/l. The remaining therapy is given once the count is fully recovered. If at the beginning of weeks 17 and 25, ANC is less than 0.5 x 10^9/l or platelets less than 50 x 10^9/l, it is permissible to delay the high dose oral methotrexate by a week.

However if counts are not requisite after a week, omit this for this cycle. Any **serious** infection, such as varicella, Pneumocystis pneumonia, or neutropenia with fever, and presumed or proven infection, warrants chemotherapy interruption at any time.

Please note standard dose oral methotrexate is to be avoided on the weeks that high dose oral or intrathecal methotrexate is used.

Children who have received cranial irradiation do not receive any further intrathecal methotrexate. For these patients, Intrathecal Methotrexate On d1 of week 14, week 20 and week 22, will be replaced by oral methotrexate, 20 mg/m^2 taken once weekly.

NOT FOR CLINICAL USE

MAINTENANCE

Phase VI

NOT FOR CLINICAL USE

ALL R3 — Phase VI - Maintenance

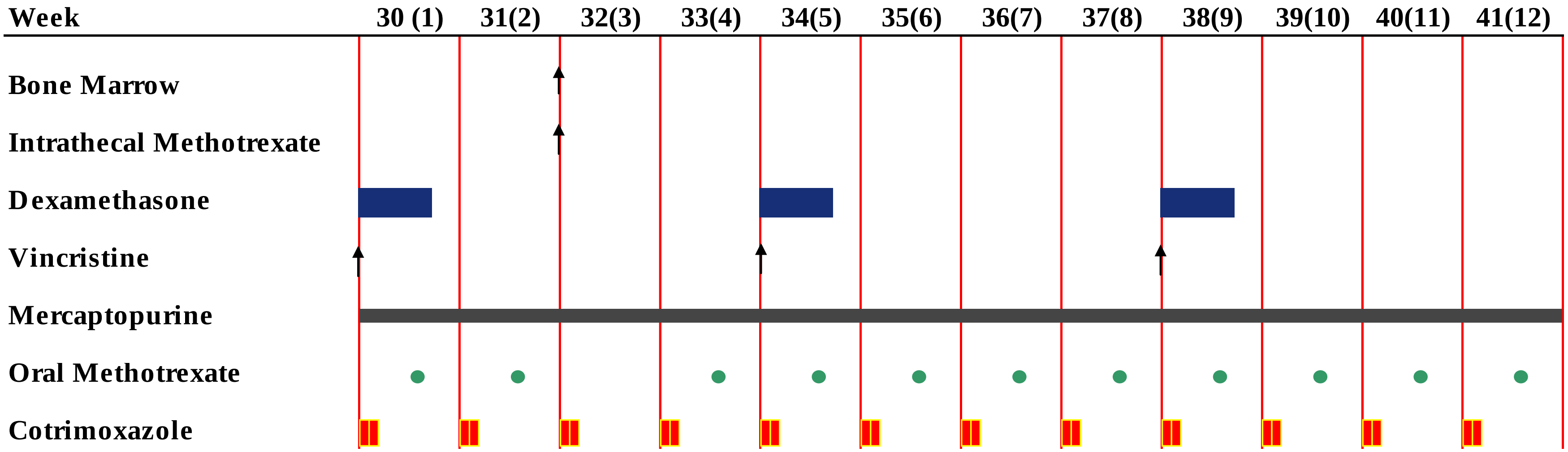

Intrathecal Methotrexate on d1, week 3. <2yrs 8mg; 2yrs 10 mg; ≥ 3 yrs 12 mg
(NB Intrathecal Methotrexate may not be scheduled with Vincristine. Do not give oral methotrexate on the same week as intrathecal methotrexate)

Note, children who have received cranial radiotherapy in R3 do not receive intrathecal methotrexate during this phase (see alternative flow sheet)

Dexamethasone, d1-5 Wk 1, 5, 9. 6 mg/ m^2 orally in 2 divided doses orally
Vincristine on d1, Wk 1, 5, 9. 1.5mg/ m^2 iv bolus **MAX 2mg as a single dose**
Mercaptopurine every day. 75 mg/ m^2/ day orally
Oral Methotrexate once weekly. 20 mg/ m^2 (except on week of intrathecal methotrexate)
Cotrimoxazole twice daily on two consecutive days every week

Note duration of treatment is exactly for 104 weeks from the start of phase V
Each maintenance cycle of 12 weeks is repeated until 104 weeks are complete
This will mean that there are 7 cycles of 12 weeks and 4 weeks of cycle 8
The last dose of intrathecal methotrexate is in cycle 7 and the last dose of Vincristine on Cycle 8 week 1

Note this flow diagram is only provided as a guide. It is mandatory to read the corresponding pages of the protocol for a more details description.

ALL R3

Phase VI - Maintenance for those post Cranial XRT

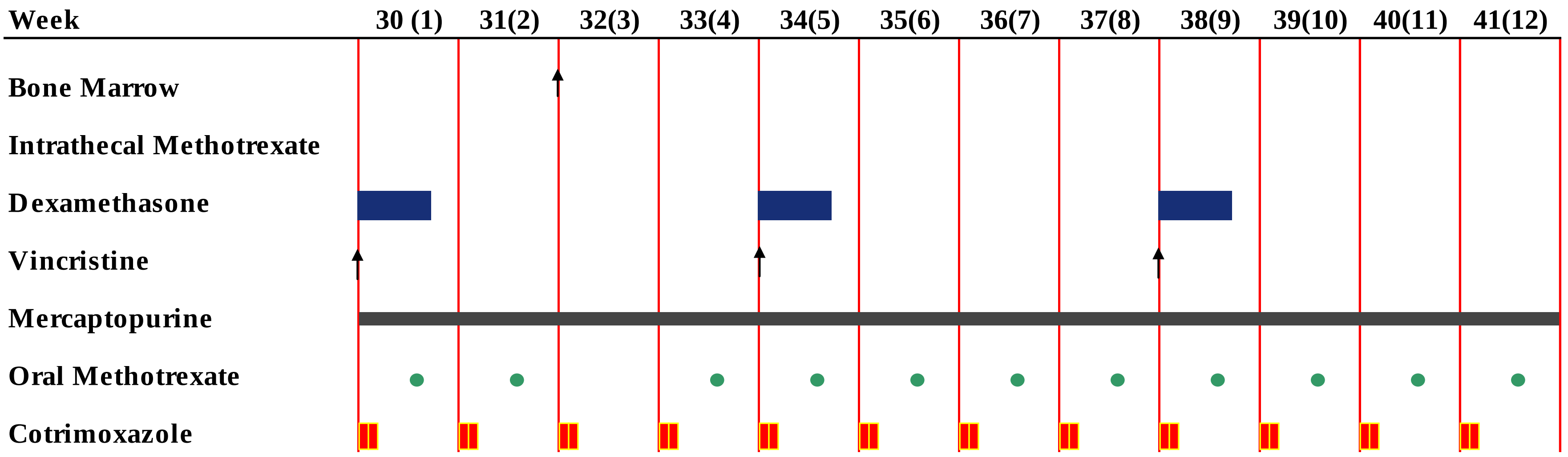

Note, children who have received cranial radiotherapy in R3 do not receive intrathecal methotrexate during this phase (see alternative flow sheet)

Dexamethasone, d1-5 Wk 1, 5, 9. 6 mg/ m2 orally in 2 divided doses orally
Vincristine on d1, Wk 1, 5, 9. 1.5mg/ m2 iv bolus **MAX 2mg as a single dose**
Mercaptopurine every day. 75 mg/ m2/ day orally
Oral Methotrexate once weekly. 20 mg/ m2
Cotrimoxazole twice daily on two consecutive days every week

Note duration of treatment is exactly for 104 weeks from the start of phase V
Each maintenance cycle of 12 weeks is repeated until 104 weeks are complete
This will mean that there are 7 cycles of 12 weeks and 4 weeks of cycle 8
The last dose of intrathecal methotrexate is in cycle 7 and the last dose of Vincristine on Cycle 8 week 1

Note this flow diagram is only provided as a guide. It is mandatory to read the corresponding pages of the protocol for a more details description.

8.6. *Phase VI : Maintenance*

Patients entering this part of the study, will be those in whom bone marrow transplant is not being considered. After the completion of Phase V, Maintenance should begin when the ANC is ≥ 0.75 x 10^9/l and the platelet count is ≥ 75 x 10^9/l. Only the mercaptopurine and oral methotrexate will be interrupted for myelosuppression and not made up. Days off therapy for intercurrent infections are counted as days off maintenance and not made up.

Anaemia occurring in the course of maintenance therapy should be treated with transfusion and the dose of drug maintained. If *persistent anaemia* occurs (i.e., haemoglobin below 8 g/dl) investigate for parvovirus infection. Please contact trial coordinators for advice.

NB. Children, who have received Cranial Irradiation for CNS disease, do not require any further intrathecal medication.

Bone marrow aspirates
Bone marrow aspirates will be done for each cycle along with the intrathecal methotrexate for follow up analysis of MRD. Please note that marrows should only be done when counts are ANC >0.5 x 10^9/l and Platelets >50 x 10^9/l with evidence of count recovery.

Continuing therapy
Each cycle lasts 12 weeks and there are 7 complete cycles. The 8th cycle is of 4 weeks duration. Please stop once 104 weeks are reached.

a) Vincristine 1.5 mg/m² **(maximum single dose 2 mg)** IV on d1 of week 1, d1 of week 5 and d1 of week 9.

b) Dexamethasone 6 mg/m² for 5 days, d1-5 of week 1, d1-5 of week 5 and d1-5 of week 9, in two divided doses.

c) Intrathecal Methotrexate On d1 of week 3.
Dose by age:
<2yrs: 8mg;
2 yrs: 10mg
> 3yrs: 12 mg.

The last intrathecal methotrexate is on d1, week 3 of cycle 7.
(NOT for children who have received cranial irradiation)

d) Mercaptopurine 75 mg/m² orally every day. Doses should be taken at least one hour after the evening meal without milk products. Dose adjustments are described in Appendix 5.

e) Oral methotrexate 20 mg/m² orally once weekly, as a single dose. Note none is given in the third week of each cycle as an

NOT FOR CLINICAL USE

intrathecal dose is given during that week except in cycle 8 (see below). Dose adjustments are described in Appendix 5.

f) Cotrimoxazole given twice daily on two consecutive days per week

Surface area	Co-trimoxazole	Trimethoprim	Sulphamethoxazole
0.5-0.75m^2	240 mg bd	40 mg bd	200 mg bd
0.76-1.0m^2	360 mg bd	60 mg bd	300 mg bd
over 1.0m^2	480 mg bd	80 mg bd	400 mg bd

The last intrathecal methotrexate is given on cycle 7. The 8th cycle is of 4 weeks duration only and there is no intrathecal methotrexate during this cycle, instead oral methotrexate will be given on week 3 of this cycle. It is permissible to complete the end of treatment assessment for MRD with the intrathecal methotrexate in cycle 7.

Children who have received cranial irradiation do not receive any further intrathecal methotrexate. For these patients, Intrathecal Methotrexate On d1 of week 3, of each cycle will be replaced by oral methotrexate, 20 mg/m2 taken once weekly.

NOT FOR CLINICAL USE

9. ALLOGENEIC STEM CELL TRANSPLANT GUIDELINES

9.1. *Eligibility*

1. All those in the High-Risk Group
2. Those in the Intermediate-Risk Group who have a MRD level of $\geq 10^{-4}$ at day 35 and have a matched donor
3. Those in the Intermediate-Risk Group, in whom the day 35 MRD results are indeterminate or where this MRD cannot be performed are eligible for an allo-SCT if, relapse occurred while still on treatment with the frontline protocol and if there is a matched donor available.

9.2. *Protocol Outline*

Week 1 -14	Relapse protocol. Count recovery should occur by week 14.
Week 13-14	Back up Harvest and pre-transplant MRD assessment
Week 15	Pre-transplant intensification window therapy will be offered to those who are MRD Positive ($\geq 10^{-3}$) at week 13. Those who are MRD Negative ($\leq 10^{-3}$) at this point proceed to standard transplant conditioning.
	**Subsequent week numbering refers to those NOT receiving FLAD**
Week 16.5	Reassessment marrow (this will not guide patient management but will be used to assess the efficacy of intensified pre transplant therapy in reducing MRD levels).

All patients should have echocardiography performed prior to the next stage. In the event echocardiography shows greater than 20% reduction in ejection fraction the echo should be repeated a week later. The Chief Investigator should be informed of any cardiac toxicity noted, either clinically or on echocardiogram/ECG.

Week 17	Proceed to transplant conditioning - protocol depends upon donor type (see below).
Week 17/18	Day 0
Week 21/22	(SCT + 28 days) Chimerism study. Adoptive immunotherapy is instituted if mixed chimerism is detected. Donor type and transplant conditioning determine method of adoptive immunotherapy.

9.3. *Definition of Matching*

The type of donor selected for BMT will depend upon donor availability and local expertise. For the purpose of this trial a fully matched donor is defined as a 10 antigen match (serological match at A and B, molecular match at C, DR and DQ).

9.4. *Cell Dose*

Both total stem cell dose and T-cell dose are critical to the success of transplantation protocols. For this trial, recommendations for the cell dose are as outlined below:

NOT FOR CLINICAL USE

Unmanipulated bone marrow grafts (siblings and matched unrelated bone marrow) should contain 3 - 4 x 10^6/kg CD34+ cells.
Haplotype transplants should contain 10 x 10^6/kg CD34+ cells after Miltenyi graft processing.
Peripheral Blood Stem Cell transplants should contain 5 x 10^6/kg CD34+ cells after Miltenyi graft processing.
The CD34 doses are calculated so as to produce adequate T-cell depletion to avoid acute GvHD whilst providing a high enough stem cell dose to ensure engraftment despite T-cell depletion.

NOT FOR CLINICAL USE

9.5. *Conditioning Regimens and GvHD Prophylaxis*

9.5.1. *Matched Sibling Marrow Transplants (Protocol A)*

These grafts will be T-replete with no *ex-vivo* graft manipulation. Recipients will be conditioned with Cyclophosphamide and TBI. Ideally the graft should contain 3 - 4 x 10^6/kg CD34+ cells. Cyclosporin and Methotrexate are given as post transplant GvHD prophylaxis.

Cyclophosphamide	60 mg/kg day –6 and –5 with hydration and MESNA
TBI	14.4 Gy in 8 fractions, 180 cGy bd days –3, -2, -1, and 0
Unmanipulated bone marrow	day 0

GvHD prophylaxis

Cyclosporin	3 mg /kg iv in 2 divided doses from day -1
Methotrexate	15 mg/m^2 iv day 1 (+ folinic acid rescue)
Methotrexate	10 mg/m^2 iv day 3, 6, 11 (+ folinic acid rescue)

9.5.2. *Matched Unrelated Marrow Transplants (Protocol B)*

These grafts will be T-replete with no ex-vivo graft manipulation. Recipients will be conditioned with Cyclophosphamide and TBI. Campath 1H will be given from day –7 to –3 for those receiving unrelated transplants. Ideally the graft should contain 3 - 4 x 10^6/kg CD34+ cells. Cyclosporin is given as post transplant GvHD prophylaxis.

Campath 1H	0.3 mg/kg over 2 hours in 2 divided doses, day – 7 to –5
Cyclophosphamide	60 mg/kg day –6 and –5 with hydration and MESNA
TBI	14.4 Gy in 8 fractions, 180 cGy bd days –3, -2, -1, and 0
Unmanipulated bone marrow	day 0

GvHD prophylaxis

Cyclosporin	3 mg/kg iv in 2 divided doses from day -1

Methotrexate is not required for a fully matched (10/10) donor.

9.5.3. *Mismatched Unrelated Marrow Transplants (Protocol C)*

These grafts will be T-replete with no ex-vivo graft manipulation. Recipients will be conditioned with Cyclophosphamide and TBI. Campath 1H will be given from day -7 to -3 for those receiving unrelated transplants. Ideally the graft should contain 3-4 x 10^6/kg CD34+ cells. Cyclosporin and Methotrexate are given as post transplant GvHD prophylaxis

Campath 1H	0.3 mg/kg over 2 hours in 2 divided doses, day –7 to –5
Cyclophosphamide	60 mg/kg day –6 and –5 with hydration and MESNA
TBI	14.4 Gy in 8 fractions, 180 cGy bd days –3, -2, -1, and 0
Miltenyi CD34+ selected marrow	day 0

GvHD prophylaxis

Cyclosporin	3mg/kg iv in 2 divided doses from day -1
Methotrexate	15mg/m^2 iv day 1 (+folinic acid rescue)
Methotrexate	10mg/m^2 iv day 3, 6, (11) (+folinic acid rescue)

9.5.4. *Haplo-identical Transplants (Protocol D)*

It is recommended that G-CSF mobilised peripheral blood stem cells (PBSC) are used in this context. These grafts will undergo T-depletion with Miltenyi CD34+ cell selection. Conditioning will consist of standard Cyclophosphamide and TBI, with the addition of Fludarabine to reduce the risk of rejection. Additional serotherapy with ATG should be given. Ideally the graft should contain at least 10 x 10^6/kg CD34+ cells. Only a short course of GvHD prophylaxis will be used because of the significant T-depletion involved in Miltenyi graft processing.

Anti Thymocyte Globulin Serotherapy

Fludarabine	25 mg/m^2 daily for 5 days. (30 minute intravenous infusion in 100mls 0.9% saline), day -13 to -9
Rabbit ATG	5 mg/kg for 5 days, day -2, -1, 0 , +1 and +2
Cyclophosphamide	60 mg/kg day –6 and –5 with hydration and MESNA
TBI	14.4 Gy in 8 fractions, 180 cGy bd days –3, -2, -1,and 0
Miltenyi CD34+ selected marrow	day 0

9.5.5. *Sibling PBSC and Matched Unrelated PBSC Transplants*

It is recommended that sibling PBSC's are conditioned according to protocol A as per sibling bone marrow transplants, and that matched unrelated PBSC's conditioned according to protocol D with T-cell depletion.

NOT FOR CLINICAL USE

10. ADOPTIVE IMMUNOTHERAPY

10.1. *Eligibility*

All those who have received an allo-SCT.

10.2. *Definition of Chimeric Status*

Complete chimerism:	Only donor DNA detectable
Mixed chimerism:	Both donor and recipient DNA detectable
Increasing Mixed chimerism:	Recipient DNA increases by 5% or more compared to previous sample

10.3. *Grades of GvHD*

Grade	Rash	Bilirubin	Diarrhoea
1	Maculopapular rash <25% body surface	35-50 µmol/ L	500-1000 ml/ day or nausea (± vomiting)
2	Maculopapular rash 25-50% body surface	51-100 µmol/ L	1000-1500 ml/ day
3	Generalized Erythroderma	101-255 µmol/ L	>1500 ml/ day <u>or</u> cramps <u>or</u> blood <u>or</u> ileus
4	Generalized erythroderma with bullous formation and desquamation	>255 µmol/ L	Simultaneous presence of any 2 of the 4 criteria for stage 3.

10.4. *Definition of Response*

Response to immune modulation is to be defined as either :
A return to complete chimerism or stable low level mixed chimerism (≤2% recipient)

OR

Fall in MRD to ≤0.01% in blood or 0.1% in marrow.

10.5. *Cessation of Immune Suppression*

Cyclosporin or FK506 should be stopped without weaning.
Steroids should be weaned as rapidly as possible without compromising the adrenal response.

NOT FOR CLINICAL USE

10.6. *Immunotherapy Study Design*

Measure: PB Chimerism and PB MRD two weekly, BM MRD monthly

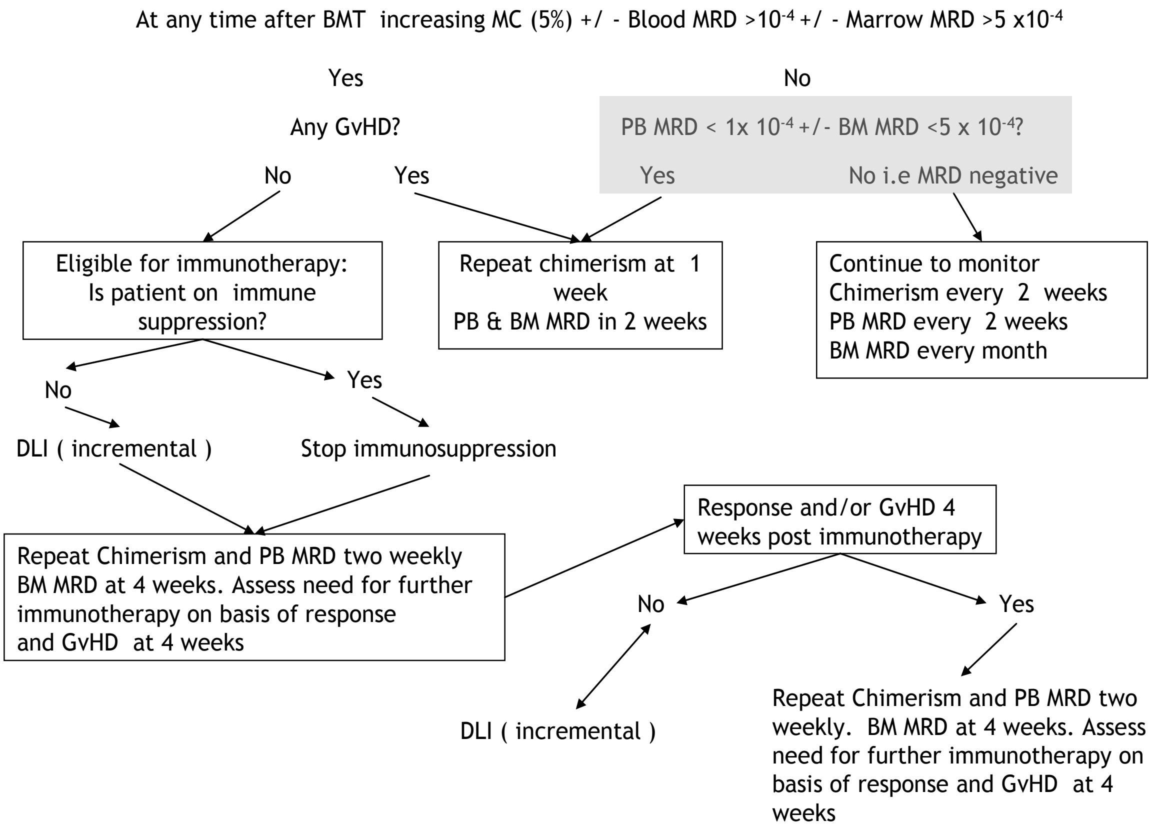

10.7. *DLI Schedule*

Incremental DLI doses are to be used until a response and/or GvHD has occurred. The initial dose is at the threshold for GvHD.

NOT FOR

	DLI Dose CD3/Kg				
**Donor type**	Level 1	Level 2	Level 3	Level 4	Level 5
Sibling	1×10^6	2.5×10^6	5×10^6	1×10^7	1×10^8
10/ 10 UD	2.5×10^5	5×10^5	1×10^6	5×10^6	1×10^7
9/ 10 UD	1×10^5	2.5×10^5	5×10^5	1×10^6	5×10^6
Haplo	2.5×10^4	5×10^4	1×10^5	2.5×10^5	5×10^5

10.8. *Data Collection*

Additional data collection will be carried out post allo-SCT to analyse the outcome of the post-transplant adoptive immunotherapy.

Please note:

1. All chimerism studies to be done locally. MRD samples to be sent to the local MRD laboratory.

2. Dose scheduling of DLI has been intentionally omitted due to the complexity of this therapy. Prior to embarking on DLI therapy, please contact the transplant coordinators, Dr Phil Darbyshire and/or Dr Nick Goulden to discuss. Please advise the Trial Manager of any patient who has received DLI .

NOT FOR CLINICAL USE

11. TRIAL MANAGEMENT AND REPORTING

ALLR3 is coordinated by the Trial Manager (Carly Leighton) and the Trial Coordinators. Additional trial management support is provided by Marie Reeves (Manchester). The trial runs on a web-based remote data entry database with decision support. Users are able to log in and register new patients, randomise and receive a unique patient trial number. The database automatically assigns the risk group, which requires verification from the investigator. The trial manager, MRD and cytogenetic laboratories are alerted by the database of new patient registrations via an automatically generated e-mail.

The database is monitored and managed by Cancer Research UK and is fully GCP and FDA compliant.

Usernames and passwords can be obtained by contacting the Trial Manager on +44 161 446 3093. Scheduling of the trial is in phases and data for each phase should be completed online within 2 weeks of phase completion. Data is analysed every 6 months and submitted to an independent data monitoring committee (DMC).

Paper Case Record Forms (CRFs) are no longer accepted at the coordinating unit as all data should be entered online. Paper CRFs may still be used to help collect the data that is to be entered online, and to assist with data checking.

All documents pertaining to the trial are required to be stored by the centre for 15-years after the final report has been published.

11.1. *National Participating Centres (United Kingdom and Ireland)*

In UK and Ireland, all 21 recognised United Kingdom Children's Cancer Study Group centres are eligible to participate. Each centre will have a named principal investigator responsible for the trial at that centre. Sponsorship for the trial is currently provided by the Barts and the London NHS Trust but will devolve to the Leicester University NHS Trust in 2006 so as to be uniform with other CCLG protocols.

11.2. *International Participating Centres and Contact Details*

Centres from the Netherlands, Australia and New Zealand will also be participating in this trial. For purposes of this trial, the centres in Australia and New Zealand are considered to be one group. Each group is responsible for their own infrastructure with regards to sponsorship, recruitment, data collection, trial regulation and minimal residual disease analysis.

In The Netherlands, Professor Peter Hoogerbrugge will represent the Dutch Children's Oncology Group as the Principal Investigator. All enquiries regarding the trial in The Netherlands should be directed to either Prof. Hoogerbrugge or to The Trials Office :

Dutch Childhood Oncology Group
Sr. Data Manager Astrid van Sonsbeek
PO Box 43515
2504 AM The Hague
Tel. +31 (0)70 367 45 45
Fax +31 (0)70 367 08 68
email: avsonsbeek@skion.nl or the central email address: registratie@skion.nl

NOT FOR CLINICAL USE

For Australia and New Zealand, Professor Tom Révész will be taking the responsibility of Principal Investigator. All enquiries regarding the trial in Australia and/or New Zealand should be directed to Professor Tom Revesz and/or:

Barbara Chamberlain
Clinical Research Associate
Department of Clinical Haematology/Oncology Children, Youth and Women's Health Service
Women's and Children's Hospital
72 King William Road
North Adelaide SA 5006
Phone: +61 8 8161 7327
Fax: +61 8 8161 6865
Email: barbara.chamberlain@cywhs.sa.gov.au

The responsibility of Principal Investigator will include circulating the protocol, any amendments or information and responding to SAE's. [SAEs should be reported as soon as possible to the Coordinating Centre [UK] and the CI, by faxing the appropriate form.] Variations in the protocol to fit in with local practices are acceptable provided they have been agreed to by the Chief Investigator.

11.3. *Randomisation*

ALLR3 uses automated stratified randomization to ensure that equal numbers are randomized within each risk group. Randomisation is also separated by country, UK and Ireland patients are randomized together; Australia and New Zealand randomized together; and there is a separate randomization for patients in The Netherlands.
The database will be common to all participants. International centres will be given privileges similar to UK centres with regards to registration and visualization of data.

11.4. *Data Monitoring Committee (DMC)*

The Data Monitoring Committee consists of, Professor Mike Stevens, Dr Rob Edwards and Mrs Moira Stewart. The Trial Manager reports to the DMC twice a year, an interim report in October and an annual report in April. The BFM and CCLG exchange an annual report of the trial in April and the BFM report is also sent to the DMC. The DMC is to return reports to the Medical Research Council Leukaemia Trial Steering Committee (LTSC) in May of each year. The DMC and LTSC will oversee the entire trial, including the data from the international collaboration.

11.5. *Reports*

The Trial Manager also submits returns to the National Cancer Research Network (NCRN) and submits written reports to the Children's Leukaemia Working Party (twice a year); NCRN (once a year); the BFM (once a year) and MREC (once a year). Copies of all reports are sent to the sponsor.

11.6. *Toxicity Monitoring*

Toxicity will be graded according to the Common Toxicity Criteria (CTC v3.0). Some Grade 3 or 4 toxicity is expected eg myelosuppression and mucositis during Consolidation and Intensification. However, any unexpected Grade 3 or 4 CTC toxicity or SAE should be immediately reported to the Trial Manager or Trial Coordinators. All SAEs received will be discussed and classified by the Chief and Co-Investigators as either a SAE, AE or SUSAR in accordance with the following definitions:

NOT FOR CLINICAL USE

11.6.1. *Adverse Events (AE)*

Any untoward medical occurrence in a patient or clinical trial subject administered a medicinal product and which does not necessarily have a causal relationship with this treatment. An adverse event can therefore be any unfavourable and unintended sign (including an abnormal laboratory finding), symptom, or disease temporarily associated with the use of an investigational medicinal product, whether or not it is considered to be related to the investigational medicinal product.

This includes (but is not limited to) all non-life threatening, non-haematological (excluding coagulation) toxicities of > Grade 3, using the Common Terminology Criteria (CTC). These will be checked by a trial coordinator, recorded but not reported, unless specifically asked for.

11.6.2. *Adverse Reaction (AR)*

All untoward and unintended responses to an investigational medicinal product related to any dose administered. All adverse events judged as having a reasonable causal relationship to a medicinal product qualify as adverse reactions. The expression reasonable causal relationship means to convey in general that there is evidence or argument to suggest a causal relationship, i.e. the relationship cannot be ruled out.

This includes (but is not limited to) all non-life threatening, non-haematological (excluding coagulation) toxicities of > grade 3 that are known toxicities of the drugs being used. Examples are Asparaginase and thrombosis, pancreatitis; dexamethasone and hypertension, hyperglycemia; Cyclophosphamide and haemorrhagic cystitis etc. These will be checked by a trial coordinator, recorded but not reported, unless specifically asked for.

11.6.3. *Unexpected Adverse Reaction (UAR)*

An unexpected adverse reaction is an AR, the nature or severity of which is not consistent with the applicable product information (e.g. investigator's brochure for an unapproved investigational product or summary of product characteristics for an approved product). When the outcome of the adverse reaction is not consistent with the applicable product information, this adverse reaction should be considered as unexpected.

These will be checked by a trial coordinator, recorded and reported on an annual basis to the Sponsor, DMC, MREC and MHRA.

11.6.4. *Serious Adverse Event (SAE)*

Each serious adverse event must be reported by the treatment centre to the trial manager within 24 hours of knowledge of event, even if it is felt not to be treatment related (see definitions below). An SAE form needs to be completed either on line on the remote data entry system or on paper. Paper forms should be faxed to the trial manger on (+44) 0161 446 3092. Follow up information about a previously reported SAE must also be reported within 24 hours.

SAE Definition

Any untoward medical occurrence or effect that at any dose results in any of the following outcomes:

Death – but not due to progression of disease
Is life-threatening
Requires admission to PICU and life support measures

NOT FOR CLINICAL USE

A new, persistent or significant disability or incapacity
Requires inpatient hospitalisation or prolongation of existing hospitalisation

Life-threatening in the definition of a serious adverse event or serious adverse reaction refers to an event in which the subject was at risk of death at the time of event; it does not refer to an event which hypothetically might have caused death if it were more severe. A temporal relationship of the onset of the event, relative to administration of the product, is not sufficient if another cause can by itself explain the occurrence of the event.

All SAE's will be initially categorised as suspected. They will need to be investigated by the trial coordinators and categorised as follows:

Unlikely – will be documented and no further action taken

All the rest will be treated as SAEs:

Possibly related - event could have been due to another, equally likely cause.
Probably related - event is more likely explained by the treatment than by another cause.
Definitely related - event can only be a result of the treatment

All SAE's will be reported to the sponsor once the trial coordinators have filed a report. They will be reported twice a year to DMC and MREC.

NOT FOR CLINICAL USE

ALLR3 – SERIOUS ADVERSE EVENT OR SUSAR

PATIENT INITIALS: |___|___| TRIAL NUMBER: |___|___|___|___|

D.O.B: |___|___| |___|___|___| |___|___|___|___| CENTRE: ____________

D D M M M Y Y Y Y

A **Serious Adverse Event (SAE)** is any event that results in any of the following outcomes:

- Death – Excluding progression of disease (which should be reported by e-mail)
- Is life-threatening,
- Requires admission to PICU and life support measures
- Results in a new persistent or significant disability or incapacity
- Requires inpatient hospitalisation or prolongation of existing hospitalisation[†].

[†]Hospitalisation due to febrile neutropenia alone should not be reported.

A **Suspected Unexpected Serious Adverse Reaction (SUSAR)** is a serious adverse reaction, the nature or severity of which is not consistent with the applicable product information (eg Investigator's Brochure for an unapproved investigational medicinal product).

SAEs must be reported within 24h of knowledge of event to the **TRIAL MANAGER:**
Fax **+44 (0) 161 446 3092** or entered **online R3 Remote Data Entry system**

PHASE OF TRIAL: ________________ **WEEK OF TRIAL:** _____ **EVENT START DATE:** ___/____/____

EVENT NAME:
☐ **DEATH**
☐ **LIFE THREATENING**
☐ **PICU**
☐ **SIGNIFICANT DISABILITY/INCAPACITY**
☐ **PROLONGED HOSPITALISATION**

DESCRIPTION OF EVENT Please supply as much information as possible (include corrective measures):

____
____
____
____

CAUSALITY was the event related to treatment**:**
DEFINITELY ☐ PROBABLY ☐ POSSIBLY ☐ UNLIKELY☐ NOT RELATED☐
If definitely/probably/possibly, name the drug/s involved: ________________________

OUTCOME:
RESOLVED ☐ EVENT END DATE: ___/___/_____
RESOLVED with sequelae ☐ give details:
ONGOING ☐ Give details:
DEATH ☐ (Please complete Death & Withdrawals form and provide report)

Should you wish to supply additional information, please attach on separate sheet/s.

REPORTED BY: ____________ **DATE:** ____/____/____ **Ph:** _________________

CONSULTANT: _____________________ **DATE:** ____/____/____ **Ph:** _________________

FOR TRIAL MANAGER/CO-ORDINATOR USE ONLY:

DATE REPORT RECEIVED: ___/____/_____ RECEIVED WITHIN 24 H? Y ☐ N ☐
FOLLOW-UP: ____

☐ SAE ☐ SUSAR (fatal/life-threat) ☐ AE/ADR

Faxed/Emailed to Trial Co-ordinators ☐ Date: ___/___/_____
Agree with categorisation of SAE Y ☐ N ☐

NOT FOR CLINICAL USE

12. STATISTICAL CONSIDERATIONS

The previous relapse trial for childhood ALL in the United Kingdom closed in 1995. Given the success and evolution of frontline protocols, a fresh approach to relapsed ALL was required. ALLR3 was built on the experience obtained from the previous trial and from the observations made by other large trial groups in the USA and Europe, together with more recently available scientific data. Thus the trial is in a large part a feasibility study with descriptive statistics. Are we able to give the treatment? Will the parents (patients) be happy to enter the trial with the options for allo-SCT? Does it provide better results then we have achieved so far (historical controls)? The trial is designed to collect real-time data and has the strength to rapidly acquire large amounts of data that can be analysed at defined time points.

12.1. *Primary Analysis*

Analysis is largely descriptive and it is intended that this trial will provide baseline data and generate hypotheses for future trials.

1. Evaluate Progression Free Survival (PFS) for all UK patients, stratified by risk groups. PFS is defined throughout as the time from trial entry to the first occurrence of progression, relapse, death in CCR or second malignancy.
2. Evaluate whether a MRD level of 10^{-4} is a suitable criterion at the end of Induction, on which to decide whether chemotherapy or stem cell transplant (SCT) will be most beneficial to patients in the intermediate risk group.

In looking at the prognostic abilities of MRD, only patients with an MRD result at day 35 will be included. BMT will be included as a time-dependent 'nuisance' covariate.

12.2. *Secondary Analysis*

1. MRD as a surrogate marker for treatment response and for PFS.

   Evaluate randomised comparison between Mitoxantrone and Idarubicin for UK patients using PFS, D35 MRD and toxicity as response variables.

   Evaluate randomised comparison between Mitoxantrone and Idarubicin for all patients (UK, Dutch, Australian/New Zealand) using PFS, D35 MRD and toxicity as response variables.

2. Evaluate Progression Free Survival (PFS) for all patients (UK, Dutch, Australian/New Zealand), stratified by risk groups.
3. Evaluate PFS and Overall Survival (OS) between R2 and ALLR3. For all patients adjusted for risk groups and then for the intermediate risk group.
4. Evaluate OS and PFS between ALLR3 and I-BFM.
5 Evaluation of FLAD: whether it reduces tumour load, and how it affects outcome following transplant.

12.3. *Sample Size and Analyses*

The power of this study is recognised to be insufficient for some of the questions. It is expected that the sample size will be approximately 350 patients (175 per treatment arm) from the UK if the trial is continued until 2010. The addition of The Netherlands and the

NOT FOR CLINICAL USE

Australian/New Zealand groups is expected to add 30 new patients to the trial per year. Thus a total of 470 patients may be expected.

With 219 patients in each group, there would be 80% power to find a difference between MRD rate of 90% in treatment A (Mitoxantrone) patients and MRD rate of 80% in treatment B (Idarubicin) patients, significant at the 5% level. The target sample size of 219 patients per treatment arm for Idarubicin and Mitoxantrone is calculated to give 80% power to find a difference in MRD rate (ie proportion of patients with negative MRD at 5 weeks) between treatment arms, where one group has a rate of 90% and the other, 80%. At present some 90% of patients in the trial are being randomised; the others will be excluded from any analysis comparing the two randomised drugs. The trial is thus powered to answer this question.

Comparisons between R2 and R3 and also between R3 and the BFM group will have a power around 80%, but these comparisons are not randomised. All other objectives are underpowered. Given the nature of the disease, the changing aspects of management and the small number of patients (even with the BFM collaboration) it will be difficult to recruit sufficient patients over a reasonable time-scale to bring real power to the study. To compare the results from the I-BFM vs R3 regimens and assuming a 10% difference in survival from 70% to 80%, we would need 296 patients per group. For response rate, in the intermediate group from 50% to 60% we would need 408 in each arm, and for the high risk group from 10-20%, we would need 219 in each arm. Each of these calculations uses 80% power and α5% level of significance.

It should be noted that for outcomes and objectives relating to MRD levels, the sample size to be analysed will be considerably reduced since the measurement is not available for all patients' in particular those patients with isolated extramedullary relapse or primary refractory disease. The number of patients recruited to the trial will be sufficient to generate hypotheses, but not to test them robustly. However, the trial is still able to answer questions and rationalise treatment for children with relapsed/refractory ALL.

PFS will be analysed using Kaplan-Meier (KM) plots, from which 3- and 5-year survival estimates with confidence intervals will be obtained. Cox regression will be used to examine multivariate relationships with risk group, D35 MRD and allo-SCT (as time-dependent covariates), randomisation to Idarubicin/Mitoxantrone, immunophenotype, time from diagnosis to first relapse, site of relapse, cytogenetic factors, age and sex as potential explanatory variables. Methods for analysis will include tabulation of MRD by treatment response, and a calculation of sensitivity and specificity (with confidence intervals). Multivariate modelling will also be used to account for factors such as risk group and other covariates found to affect PFS as part of primary analysis. Logistic regression will be used to explore the relationship between the drugs and D35 MRD. Incidence of toxicity for the two drug groups will be compared using a chi-square test.

12.4. *Stopping Rule*

Based on I-BFM and unpublished data from the R2 and R3 trial, the best estimate of expected mortality is 25% treatment related mortality (TRM) in the high risk patients and 10% TRM in the intermediate/standard risk patients.

NOT FOR CLINICAL USE

Following the recommendation of the Data Monitoring Committee, the stopping rule for randomisation is if the difference in number of deaths exceeds 3 standard deviations between the two randomised arms. This will be shown by a p-value of 0.001 or less on a two-sided Fisher's exact test.

NOT FOR CLINICAL USE

APPENDICES

Appendix 1 : Antifungal Prophylaxis

The approach to these children should be similar to that of induction in a child with Acute Myeloid Leukemia. They are high risk relapses potentially at risk of chemotherapy induced toxicity and infection and should be kept in hospital until count recovery is seen post-induction. **All children should receive anti-fungal prophylaxis during the phases I - III.**

During induction:

Liposomal Amphotericin 1mg/kg should be given three times weekly until count recovery.

Post count recovery after induction:

Continue with Liposomal Amphotericin 1mg/kg

OR

Elixir Itraconazole 2.5 mg/kg twice daily (Do not use capsules).
Note, Itraconazole absorption is lower in neutropenic patients and its levels are affected by many drugs. Particular caution must be applied if there is concomitant use of macrolide antibiotics and vinca alkaloids and in patients who have **compromised cardiac function**. Monitoring of levels is recommended. Either bioassay or HPLC is acceptable, however the reference range for these techniques are different and the values obtained by the two methods are not comparable.

OR

Voriconazole - Paediatric Dosage Information

Ages 2- <12yrs) Treatment and Prophylaxis
Oral/intravenous
Loading dose: 6mg/kg every 12 hours for 2 doses, then
Maintenance dose: 4mg/kg every 12 hours

Adult Dosage Information (ages >12yrs) Treatment and Prophylaxis

Intravenous
Loading dose: 6mg/kg every 12 hours for 2 doses, then
Maintenance dose: 4mg/kg every 12 hours

Oral
Patients 40kg and over:
Loading dose: 400mg every 12 hours for 2 doses, then
Maintenance dose: 200mg every 12 hours (may be increased to 300mg *po* bd)

Patients <40kg:
Loading dose: 200mg every 12 hours for 2 doses, then
Maintenance dose: 100mg every 12 hours (may be increased to 150mg *po* bd)
Notes
- For oral administration, give tablets at least one hour before, or one hour after a meal

NOT FOR CLINICAL USE

• Tablets may be crushed and dispersed and administered via a NG tube
• Duration of treatment depends on patients clinical response
• No dose adjustment for renal impairment for patients receiving oral voriconazole. However, for patients
with moderate to severe renal impairment, consideration should be given to avoiding the iv formulation as the intravenous vehicle may accumulate

When febrile neutropenic

In all cases, when febrile and neutropenic, Liposomal Amphotericin increased to 3mg/kg daily to be used empirically along with antibiotics.

NOT FOR CLINICAL USE

Appendix 2 : Biological Samples and Tests

Samples will be collected from all patients in ALL R3, both for MRD analyses and for storage. The samples stored will be available through the existing leukaemia cell bank facility via the MRD laboratories as well as through a newly created separate R3 tissue bank.

Need for a separate tissue bank

The current cell bank primarily stores material left over from the MRD analysis. There is good quality DNA available but often there is little else left to store. We are entering a new era of cancer diagnosis and detection which will be heavily dependent on RNA and protein analysis. While these techniques require very little material, they are entirely dependent on the quality of material stored. For example, proteomic techniques could be used to look for changes in the cerebrospinal fluid to identify those at a risk of CNS disease, provided the csf has been stored properly. We have, along with our colleagues in the BFM, been piloting tissue storage and collection for some time to assess the optimal conditions and cost. We will use the UK DNA Banking Network (Medical Research Council Biobank) facility to collect, catalogue and store samples for future use. The samples will be considered to be a gift to the Childhood Leukaemia Working Party (CCLG). The CCLG will be custodians of these samples. On instruction from the CCLG, the Chief Investigator will make these samples available for ethically approved and peer-reviewed research projects.

Samples

All samples will be collected at the time points where a marrow sample or lumbar puncture is required by the protocol. No additional invasive procedures are required, only the taking of an additional sample. Please note that a marrow sample should be sent to the MRD laboratory and **an additional sample** to the tissue bank.

Technique for marrow sampling

Please obtain marrow as follows. The aspirate needle should be passed fresh into 2 - 3 sites. (This can be done by re-angling the needle; new skin punctures are not required). From each site the first 1ml of marrow should be aspirated using a 2-ml syringe. The samples from each site can be combined into a single container with 1.5 ml ACDA.

NOT FOR CLINICAL USE

Mandatory sample collection (this includes all patients, even those with isolated extramedullary disease)

At Diagnosis:

Marrow/Peripheral Blood Samples:

Two 5-10 ml **marrow** samples are required at the time of diagnosis. In the order of priority, the first sample is to be sent to the MRD laboratory. The second needs to go to the tissue bank.

Send an additional 5-10 mls of **peripheral blood** to the tissue bank for plasma storage.

Both marrow and blood should be collected in 8.5ml vacutainers containing 1.5ml Acid Citrate Dextrose solution A (ACDA) anticoagulant (Becton Dickinson: Dextrose A – trisodium citrate, 22.0g/L; citric acid, 8.0g/L and dextrose 24.5g/L)

Where it has not been possible to obtain a marrow sample, or the quality of the marrow is in doubt, or if there are more than 20 x 10^6 blasts per ml in the peripheral blood then 5 – 10ml of blood should be sent to both laboratories. Samples should be taken into a vacutainer containing ACDA or an EDTA bottle and dispatched to the appropriate lab by courier.

Cerebrospinal fluid: 3-5 mls of cerebrospinal fluid to be collected in a universal sterile container with no additives. This is to be sent with the marrow sample to the tissue bank, by courier. **Do not send this to the MRD laboratory.**

Testes or other tissues: When a biopsy sample is available from extramedullary tissue, please arrange to send 2-3 fragments in culture medium to the tissue bank.

At Day 35

This is the first MRD decision time point. Please ensure that 2-5 mls of marrow is sent to the MRD laboratory. In addition send 2-5 mls of marrow, from a separate aspirate, 5 mls of peripheral blood and 2-5 mls of cerebrospinal fluid to the tissue bank.

At Week 13-14

This is the second MRD decision time point. Please ensure that 2-5 mls of marrow is sent to the MRD laboratory. In addition send 2-5 mls of marrow from a separate aspirate, 5 mls of peripheral blood and 2-5 mls of cerebrospinal fluid to the tissue bank.

Other sample collection time points

These are scheduled when bone marrow assessments and/or lumbar punctures are due. These are day 8 and 15 during induction, and at the beginning of phase II and III as well as prior to allogeneic-SCT. At these time points please send 2-5 mls of marrow to the MRD laboratory and 2-5 mls of marrow from a separate aspirate, 5 mls of peripheral blood and 2-5 mls of cerebrospinal fluid to the tissue bank.

NOT FOR CLINICAL USE

Summary

Tissue	Volume	Container
Bone Marrow (BM)	3 – 5mls	EDTA
Peripheral Blood (PB)	3 – 5mls	EDTA
Cerebrospinal Fluid (CSF)	3 – 5mls	No additive
Testes / Other Tissue (T)	2 – 3 pieces	In tissue culture medium

Mandatory Time Points

1.	Diagnosis	BM, PB, CSF, T
2.	Prior to Phase II (day 35 MRD):	BM, PB, CSF
3.	Prior to Phase III	BM, PB, CSF
4.	Week 13 MRD	BM, PB, CSF
5.	Prior to BMT	BM, PB, CSF

Biological Analyses

Minimal Residual Disease

On receipt of bone marrow aspirates collected at diagnosis or end of induction, the MRD laboratory will assign the patient and the sample a unique number according to the standard operating procedure. Cell counts will be recorded and DNA extracted within 24 hours of receipt of the sample. A minimum of 10 micrograms of DNA is required at diagnosis and 5 micrograms at end of induction. In the event that an inadequate sample is obtained then a further sample will be requested.

The study proposes to use Real-Time methods for the detection of MRD, according to BIOMED guidelines. The following definitions will be used:

Definitions

For day 35 bone marrows:

Negative	MRD is < 10-4 at a minimum of 2 loci
Positive	MRD is ≥ 10-4 at any loci
Indeterminate	MRD is < 10-4 at a single loci<br>OR if no loci has been identified

NOT FOR CLINICAL USE

MRD Laboratories and their CCLG Centres

**Bristol**	Bristol<br>Southampton<br>Cardiff<br>Leeds<br>Oxford	**Glasgow**	Glasgow<br>Liverpool<br>Aberdeen<br>Edinburgh<br>Belfast<br>Dublin<br>Newcastle
**Sheffield**	Sheffield<br>Leicester<br>Nottingham<br>Cambridge<br>Birmingham<br>**Manchester**	**Barts**	University College<br>Great Ormond St<br>Royal Marsden

Reporting of MRD Results

The Trial Manager will communicate all MRD results to the treating physician via email. Please do not contact the laboratory directly as they will be unable to provide you with this information.

Cytogenetics

Once a patient is registered on the database, the Leukaemia Research Fund Cytogenetics database will be automatically informed of a relapse. They will collect cytogenetic information from the regional laboratory. This will be correlated at a later date with the karyotype at diagnosis and other biological variables.

Asparaginase Activity

These studies will be performed in Manchester. The Asparaginase dosage schedule is designed to achieve a minimum concentration of 100 iU of Asparaginase and achieve total depletion of Asparagine during the first 12 weeks of chemotherapy. Since all children in this study will have received asparaginase previously, some may have acquired antibodies. Serum samples collected at weekly intervals will be assayed for Asparaginase activity using the Medac MAAT test. Asparaginase antibody assays will be performed using antibody capture enzyme-linked immunosorbent assay and asparagine levels will be measured concurrently. The study uses a combination of PEG and E Coli Asparaginase and the pharmacokinetics of using this in the planned schedule is unknown. The data obtained from this study will therefore permit more rational drug delivery in the future and guide a change in therapy in the presence of developing antibodies.

The MRD, cytogenetics and asparaginase activity analyses are trial questions. All other proposed studies need to be approved by the Biological Committee, Childhood Leukaemia Working Party and have received ethical approval. Studies approved at the time of the original proposal (2003) include:

NOT FOR CLINICAL USE

Other studies

Molecular Pharmacology Studies

These studies will be done in Newcastle. DNA extracted from blasts at trial entry will be analysed for the presence of single nucleotide polymorphisms (SNP) in a range of drug response related genes including Glucocorticoid receptor, topoisomerase II, MDR1, RFC and MTHFR. Key exons will be amplified by PCR and SNPs detected using denaturing HPLC. Results will be compared with constitutional DNA from patient non-malignant cells and a panel of normals already in use in our laboratory. In the case of the Glucocorticoid receptor, the presence of SNPs will also be determined in DNA from sorted blasts at day 8. The functional significance of any mutations detected will be predicted from knowledge of the published gene sequence information and where appropriate assessment of function of heterologously expressed mutants.

Microarray Analysis

All initial samples will be analysed using the Affymetrix HG-U133 oligonucleotide A and B chips, which currently has approximately 24,000 genes arrayed. Briefly at least 2 x 10^6 cells are required for each analyses. Sufficient material needs to be stored down to allow subsequent verification. We therefore need approximately 2 mls of fresh marrow for the initial analysis, but subsequent analyses will require less. As in the MRD analyses, the cells maybe collected in ACDA. Cells will be Ficoll separated and stored in liquid nitrogen prior to use. RNA will be extracted with the Trizol method. Target gene validation will be carried out using Real-Time PCR. The DNA and/or cell samples will be obtained through the MRD network and the flow cytometry study.

Chemosensitivity Assays

Chemosensitivity assays will be performed at Barts and The London using the ATP assay which uses luciferase to test for viability after drug exposure. The panel will include drugs used in this protocol as well as other novel drugs. Approximately 5 x 10^6 cells are required to test 10 drugs and the cells need to be assayed within 48 hours of collection.

Genetic Polymorphisms

Polymorphisms in drug metabolizing enzymes may adversely affect outcome. With regards to ALL R3, the most likely candidates are MTHFR; Cytochrome P450; NAD(P)H; glutathione and TPMT. Other than these, we are also asking if the genetic factors that predispose to relapse are the same or different to those which predispose to the initial leukaemia. These include DNA damage recognition and repair genes: MREII, Nibrin, Rad50 complex, RAG1/RAG2. For these genes, studies examining de novo patients are already underway at the co-ordinating centres. We also have performed preliminary work on minisatellite mutation rates (MMR) and have detected a 50% increase (in the MMR) in de novo patients. Some HLA class II alleles appear fundamental to the initiation of common ALL and T cell ALL. Other molecules of interest are Topoisomerase II and MDR1. These studies will be performed in Manchester and Newcastle.

NOT FOR CLINICAL USE

Appendix 3 : Guidelines for intermediate dose intravenous administration of Methotrexate

Please inform the trial coordinators about any child who fails to complete or has excessive toxicity with the methotrexate course.
Cotrimoxazole should be stopped 7 days before the start of the intravenous methotrexate infusion and can be recommenced 7 days after.

Rehydration
Time: Start hydration at least 6 hours prior to the commencement of the intravenous methotrexate.
Fluid: 4% glucose with 0.18% normal saline. To each 500 mls add 25 mmol of sodium bicarbonate and 10 mmol of potassium chloride.
Infusion rate: 125 mls/m^2/hr (3 L/m^2/day)

NB: Adjust the sodium bicarbonate concentration to maintain the urinary pH between 7 and 8. Do not start the infusion until a urinary pH of at least 7 has been achieved.

Dose of Methotrexate:
Dilute the methotrexate in an appropriate volume of saline (0.9%). Infuse 100 mg/m^2 of methotrexate over 15 minutes and then 900 mg/m^2 of methotrexate to be infused over 36 hours. Note, even if the infusion is not complete at this time point, it must be stopped.

Hydration during Methotrexate infusion:
Fluid: 4% glucose with 0.18% normal saline. To each 500 mls add 25 mmol of sodium bicarbonate and 10 mmol of potassium chloride.
Infusion rate: Hydration needs to continue during the 36 hours of methotrexate infusion to maintain a combined infusion rate of 125 mls/hr. This may be achieved either by using a Y extension set or using both lumens of the central venous line.

Post Methotrexate Hydration:
Continue hydration until folinic acid rescue is completed
Fluid: 4% glucose with 0.18% normal saline. To each 500 mls add 25 mmol of sodium bicarbonate and 10 mmol of potassium chloride.
Infusion rate: 125 mls/m^2/hr (3 L/m^2/day)

Methotrexate levels: Check plasma methotrexate level at 48 hours after start of the methotrexate infusion. If the level is ≤ 0.5 μmol/l ($< 1 \times 10^{-6}$ M or 0.227 μg/ml), then do not give more than two doses of Folinic Acid (48 and 54 hours). If MTX levels at 48 hours are > 0.5 μmol/l, then continue hydration and folinic acid rescue every 6 hours until MTX levels are < 0.25 μmol/l.

Folinic Acid Rescue: 15 mg/m^2 intravenously at 48 and 54 hours and subsequently only if the plasma methotrexate level is high (see above). Subsequent doses may be given orally if necessary. Intravenous hydration is stopped when the last dose of Folinic acid is given.

Intrathecal Methotrexate: This can be given before the start of the methotrexate infusion. Where necessary, to fit in with local practice, it can be given during the methotrexate infusion.

NOT FOR CLINICAL USE

If this is the case, the infusion must not be discontinued. Intrathecal methotrexate should not be given once the intravenous methotrexate infusion has been stopped at 36 hrs.

Note: Maintain output at 400 mls/m^2 for any 4 hour period.

NOT FOR CLINICAL USE

Glucarpidase (formerly Carboxypeptidase) and Methotrexate Nephrotxicity

Nephrotoxicity is an infrequent but potentially life-threatening complication of high-dose methotrexate because it can lead to delayed methotrexate (MTX) excretion and a marked enhancement of MTX-induced myelosuppression, mucositis, hepatitis, and dermatitis. It occurs probably by a combination of precipitation and direct effects on the tubule. This can result in delayed renal excretion of methotrexate and therefore sustained elevated plasma methotrexate concentrations. Folinic acid 'rescue' in this setting (high plasma methotrexate concentrations) is often inadequate as both methotrexate and folinic acid compete for the same cellular uptake pathway. Also folinic acid is insoluble in acid urine and high doses have been associated with cardiac disturbances secondary to electrolyte disturbances. If there is a rising creatinine (>100% in 24 hours) or the 48 hour methotrexate level is >10 μ/l consider using glucarpidase. The glucarpidase enzyme cleaves the terminal glutamate from folate and folate analogues such as methotrexate. In the case of methotrexate nephrotoxicity, glucarpidase action results in the production of an inactive metabolite (DAMPA).

Stop folinic acid 2 hours before administering glucarpidase as it is a competitive substrate and may compete with MTX for glucarpidase binding sites.

Dose of glucarpidase: 50 units/kg administered by intravenous bolus over 5 minutes. Reconstitute each vial with 1ml sodium chloride 0.9% (do not further dilute). Each vial contains 1000IU/ml (after reconstitution) and round dose up to vial size. No further dose is required.

Maintaining alkalinisation of urine with sodium bicarbonate is essential to maintain urinary pH>7.

It is essential that patients are NOT co-prescribed the following medicines which reduce MTX excretion: NSAIDS, aspirin, ciprofloxacin, co-trimoxazole, penicillin, probenecid, omeprazole.

Folinic acid should not be administered in the 2 hours prior to or the 2 hours following the administration of glucarpidase.

2 hours after administration of glucarpidase, folinic acid should be administered at a dose of 250mg/m^2 every 6 hours by IV bolus (maximum rate: 160mg/min) for up to 48 hours and then decreased based on plasma MTX concentrations to 15mg/m^2 intravenously or orally every 6 hours until the plasma MTX concentration is less than 0.2 μmol/l.

NOT FOR CLINICAL USE

Appendix 4 : Radiotherapy

A. Cranial radiotherapy guidelines

These guidelines only apply to patients with CNS disease at presentation. Children under 2 years of age do not receive cranial irradiation.

a) Megavoltage Apparatus should be used, preferably a linear accelerator.
b) All fields should be treated on each treatment day.
c) Midplane dose 24 Gy in 15 fractions of 1.6 Gy each, in 15-21 days. (Treatment may start on any day except Friday).
d) Lateral opposed fields are used to involve all cranial meninges including those surrounding the optic nerve in the retro-orbit, and extending down the spinal cord to level of C2. Field margins should extend at least 2 cm beyond the meninges in all directions to avoid under dosage at the edges of the beam.

The dose of 24 Gy has been chosen rather than 18 Gy, as this therapy is for patients with overt CNS disease and hence is an essential part of the treatment, rather than being "prophylactic" in nature. The preferred technique is one which ensures adequate coverage of the whole of the cranial meninges while ensuring that the lens dose is kept as low as possible. The patient should be treated immobilized in a supine shell. A technique that centres on the orbit and uses customized lead blocks to minimise beam divergence is therefore preferred.

A treatment area is selected clinically which is symmetrical and lies 15 mm behind the cornea on each side. Using a simulator these 2 points are opposed and a simulator film taken for the production of customised lead blocks. These should be designed so as to treat the cervical cord down to the level of C2 and to ensure adequate treatment to the origin of the facial nerve.

The use of this technique necessitates either the use of asymmetric jaws to block the lower part of the neck or else the use of a very large amount of lead. It may therefore not be possible at all centres and in such cases a similar blocking arrangement using field centred in the midcranium are acceptable. A third alternative is to use a rectangular field with one edge running parallel to Ried's baseline.

e) Treatment to additional fields, eg nasal electrons to the cribriform plate may be used at the discretion of the clinician. If such modifications are used they should be specified on the enquiry sheet and the reason they were considered necessary in giving.

f) Dose to the lens. Although there is uncertainty as to whether thermoluminescent dosimetry (TLD) can adequately estimate the dose to the lens, it is nonetheless recommended that such dosimetry be performed and the results recorded, as it is intended to use the data collected to study cataractogenesis in long term survivors. TLDs should be placed on the patient underneath the shell, both on the eyelid in front of the position of the lens, and at the outer canthus of the eye. If possible the dose to the lens should be less than 10% of the mid-plane dose, although it is recognised that this may not always be achievable with adequate treatment of the cribriform plate. Where estimated doses are high, they should be discussed with the radiotherapy coordinator.

g) Quality control. An initial simulator film should be taken for planning purposes. Shielding block positions should where possible be checked at a second simulator session. Beam films should be taken on the treatment set to verify block positions.

NOT FOR CLINICAL USE

Simulator and beam films will be requested for review following the completion of treatment.

h) Interruptions to radiotherapy should be kept to a minimum. Treatment need not be interrupted for cytopenia unless the patient is unwell. In such cases, treatment should be re-commenced as soon as possible. Interruptions longer than 48 hours should be discussed with one of the trial coordinators.

Note: There is evidence to suggest that the use of thiopurines during cranial irradiation may predispose to the occurrence of brain tumours. Therefore, during the cranial radiotherapy, only the use of vincristine and dexamethasone is recommended and the use of thiopurines and methotrexate is to be avoided.

Those who receive cranial radiotherapy will not receive any further intrathecal methotrexate. This will be replaced by once weekly oral methotrexate , 20 mg/m^2 in the week that intrathecal methotrexate would have been scheduled in phases V and VI.

NOT FOR CLINICAL USE

B. Testicular radiotherapy guidelines

a) Megavoltage or Orthovoltage apparatus may be used.

b) As in previous MRC studies, the volume should include the testes and the spermatic cord to the level of the deep inguinal ring with lead shielding to surrounding tissues including the penis. An applied field is used. The use of bolus should be considered depending on the energy of the radiation and the size/age of the child.

c) The dose will be 24 Gy in 12 daily fractions of 2 Gy. This should be given during weeks 14-16 (post Intentensification). In patients receiving cranial RT, testicular irradiation should be given concomitantly with cranial irradiation.

Interruptions to radiotherapy should be kept to a minimum. Treatment need not be interrupted for cytopenia unless the patient is unwell. In such cases treatment should be re-commenced as soon as possible. Interruptions longer than 48 hours should be discussed with one of the trial coordinators.

The patient will be on continuation mercaptopurine during testicular irradiation. Priority should be given to the continuation of the radiation rather than the mercaptopurine if cytopenias arise.

NOT FOR CLINICAL USE

C. Total Body Irradiation (TBI)

There is no universally recognised technique, and different centres will have their own local variations dictated by the equipment available. These guidelines do not apply to children under 2 years of age. If general anaesthesia is required the technique may need to be modified further.

a) Megavoltage apparatus should be used. The potential field size at extended distance should be large enough to cover the majority of patients comfortably.

b) A single field is treated in most sessions. The chosen fields should result in an even distribution of dose throughout the body by the end of treatment. Bolus bags, compensators and shielding may be used to achieve this, according to local technique. Doses should not vary by more than +/- 5%.

c) The dose is 14.4 Gy in 8 fractions administered twice daily on consecutive days with a minimum interval of 6 hours between fractions. The dose is prescribed in the mid-plane of the lungs (density corrected) at the level of the nipples.

d) Treatment should be planned with CT scans. Alternatively, test doses can be carried out for critical areas.

   During treatment, actual doses should be measured at entrance and exit points representative of lung, brain and kidneys (other sites optional). This may be done using diodes or thermo-luminescence dosimeters. There should also be monitoring of the machine output at the extended treatment distance.

e) The value of CNS and testicular boosts remains controversial. The rationale for boosting is to achieve a radiation dose more nearly equivalent in radio-biological terms to that which would have been given had the patient not been receiving TBI. There have been no randomised controlled trials to test the benefits or otherwise of this approach. Some centres prefer to avoid boosts because of concerns about late toxicity. It is therefore up to each centre to decide on its policy in the absence of clear evidence, and to remain consistent in its approach.

If boosts are used, the recommended doses are: cranial boost – 6 Gy in 4 daily fractions; testicular boost - 6Gy in 3-4 daily fractions (can be given concurrently). The boosts are given in the week before TBI.

NOT FOR CLINICAL USE

Appendix 5 : Mercaptopurine and methotrexate dose alterations

Only MP and MTX will be interrupted for myelosuppression. The omitted doses will not be made up. The oral doses of MP and MTX should be adjusted to maintain ANC between 0.75 and 1.5 x 10^9/l and platelets between 75 and 150 x 10^9/l.
Start at 100% MP (75 mg/m^2/day) and MTX (20 mg/m^2/week) and do not escalate. Follow dose reduction guidelines as described below.

Reductions of mercaptopurine and methotrexate during continuing maintenance
If the neutrophil count falls to between 0.5 and 0.75 x 10^9/l HALVE the dose of mercaptopurine and methotrexate. If the neutrophil count falls to < 0.5 x 10^9/l **STOP** mercaptopurine and methotrexate. **ONLY RESTART** when the count is over 0.75 x 10^9/l. **Restart** at 100% of protocol dose (not dose at which counts fell) when neutrophils > 0.75 x 10^9/l.

i) The same dose modifications apply to falling platelet counts. If the count is less than 75 but more than 50 x 10^9/l HALVE dose as above; if less than 50 x 10^9/l, STOP mercaptopurine and methotrexate. REINTRODUCE as above when the count is greater than 75 x 10^9/l.

ii) If counts fluctuate wildly when restarting @ 100% dose after cytopenias, starting at 50% and titrating upwards is permissible to avoid frequent interruptions to mercaptopurine exposure. (This manoeuvre is not often necessary).

Escalation of mercaptopurine and methotrexate during continuing maintenance therapy.

The aim is to adjust doses to maintain the ANC between 0.75 and 1.5 x 10^9/l and the platelet count between 75 and 150 x 10^9/l. If during interim maintenance the ANC > 1.5 x 10^9/l (and platelets >75 x 10^9/l) the dose of mercaptopurine should be escalated by 25% (from 75 mg/m^2/day).

If the subsequent monthly ANC is:

1) ANC > 1.5 x 10^9/l (and platelets > 150 x 10^9/l), keep mercaptopurine at the 125% dose and increase methotrexate by 25% to 25 mg/m^2/dose.

2) Continue to increase the mercaptopurine and methotrexate dose in 25% steps alternately every eight weeks as outlined above if ANC > 1.5 x 10^9/l and platelets > 150 x 10^9/l persists. There are no maximum doses for mercaptopurine and methotrexate.

NOTE: Tolerance of 150% or more of the target protocol mercaptopurine dose for prolonged periods may be indicative of partial or non-compliance, and is potentially dangerous if the patient suddenly starts to comply fully. Metabolite assays in such circumstances can be helpful to exclude non-compliance. Rare individuals (1 in 300) taking thiopurine who are congenitally lacking intracellular TPMT will show profound myelosuppression at a standard thiopurine dose. These patients will be identified prospectively at the time of diagnosis, and advice on dosing will be given by the trial co-ordinators.

NOT FOR CLINICAL USE

Thumb rules for maintenance

Dose Reduction

If ANC < 0.5 or platelets < 50

Stop both thiopurines and oral methotrexate

Weekly FBC

When ANC >0.75 and platelets > 75, restart at 100% of protocol (NOT TOLERATED) dose

If ANC >0.5 and < 0.75 or platelets > 50 and <75

Reduce dose of both thiopurines and methotrexate to 50% of protocol (NOT TOLERATED) dose

Weekly FBC
ANC > 0.75 and platelets >75

Increase dose to 100%

Dose Escalation

If ANC > 1.5 and platelets >150 throughout previous eight weeks

Increase the mercaptopurine dose by 25%

If ANC >1.5 and platelets >150 throughout the 4 weeks following mercaptopurine dose escalation, increase MTX dose by 25%

Repeat above MP/MTX 25% dose escalation in alternating eight week cycles if ANC >1.5 and platelets >150

(see protocol guidance for those with persistent neutropenia or those with wildly see-sawing counts)

SE

Appendix 6 : Pneumocystis Carinii Pneumonitis (PCP) Prophylaxis
Co-trimoxazole (trimethoprim and sulphamethoxazole)

This drug is given orally as PCP prophylaxis on 2 consecutive days throughout treatment from the start of induction. This dosage is lower than that recommended in previous studies, but has been found to be equally effective in CCG studies. The dose is tabulated below. Please ensure separation of the days on which oral Methotrexate and Cotrimoxazole doses are given during maintenance courses. If a child remains cytopenic after being off chemotherapy for three weeks or more, then stop the co-trimoxazole. Reintroduce co-trimoxazole once both thiopurine and methotrexate are back at standard dose. If cytopenias recur once the co-trimoxazole is reintroduced, then it should be stopped for at least two months and an alternative form of prophylaxis used instead (see below). The alternative drug should then be continued for the duration of the antileukaemic therapy. The maintenance of adequate doses of thiopurine and methotrexate should take precedence over continuing co-trimoxazole. However, if co-trimoxazole is stopped, it must be remembered that the child is at increased risk of PCP. Nebulised pentamidine or oral Dapsone are alternative drugs.

Surface area	Co-trimoxazole	Trimethoprim	Sulphamethoxazole
0.5-0.75 m^2	240 mg bd	40 mg bd	200 mg bd
0.76-1.0 m^2	360 mg bd	60 mg bd	300 mg bd
over 1.0 m^2	480 mg bd	80 mg bd	400 mg bd

Alternative PCP Prophylaxis

If a child must stop cotrimoxazole because of repeated cytopenias or other inability to tolerate it, PCP prophylaxis should continue with one of the alternative drugs. Data from HIV populations suggests that Dapsone is a more effective choice than nebulised Pentamidine or Atovaquone. Dapsone 2mg/kg (maximum dose 100mg) once daily or 4mg/kg weekly (maximum dose 200mg), orally is recommended as the alternative agent in patients who cannot tolerate cotrimoxazole. Side effects of dapsone include fever, rash, and haemolytic anaemia. **G6PD qualitative assay should be performed before starting dapsone therapy**. For patients who cannot tolerate dapsone, nebulised pentamidine or atovaquone is recommended. Nebulised Pentamidine 300 mg per month is given by nebuliser, using 6 ml sterile water delivered at 6 L/min until the reservoir is dry, usually over 45 minutes. Atovaquone 30mg/kg is given orally once a day with food. Side effects include gastrointestinal intolerance, rash, headache, and fever. Pentamidine 4mg/kg IV every 2-4 weeks could be used if none of the other alternatives are suitable.

NOT FOR CLINICAL USE

Comparison of PCP Prophylaxis Regimens

	**TMP-SMZ**	**Dapsone**	**Nebulised Pentamidine**	**Atovaquone**
Efficacy	high	moderate	moderate	moderate
Toxicity	moderate	low-moderate	high	low
Cost	low	low	high	very high
Protection against infection	yes	?	no	no
Risk of extrapulmonary pneumocystosis	no	no	yes	no

NOT FOR CLINICAL USE

Appendix 7 : Guidance on the use of Erwinia

A licensed preparation of Erwinia Asparaginase (Erwinase®) is now available, thus providing an effective alternative for patients with hypersensitivity to E.Coli Asparaginase. Erwinase® will be marketed and distributed by OPi pharmaceuticals. Erwinase® should be used in place of Pegylated E. Coli Asparaginase in the following circumstances:

Systemic hypersensitivity reactions to native (Medac asparaginase) or Pegylated E.Coli Asparaginase (Oncaspar). This includes patients with generalised rash with or without anaphylactic symptoms, but not those with only local pain or redness at the site of injection.

Patients with previously documented systemic reactions to E.Coli Asparaginase should receive Erwinase® in any remaining Asparaginase containing courses.

1. Each dose of Pegylated Asparaginase (Oncaspar) should be replaced with 6 doses of 20,000 Units/m^2 Erwinase®.

2. Erwinase® should be administered by intra-muscular injection. For older patients requiring large volumes, the individual dose may be split between two injection sites.

3. Please note that in Phase III, the Asparaginase recommended is Erwinase.

Please notify the trial manager of patients switching to Erwinase®.

PEG Asparaginase	Erwinase
**Induction**<br>Peg Asparaginase 1000 units/m^2 IM on day 3 of week 1 on day 3 of week 3	**Induction**<br>Erwinase 20,000 units/m^2 IM on day 3 of week 1and then Mon/Wed/ Friday for 6 doses in total .<br>Erwinase 20,000 units/m^2 IM on day 3 of week 3 and then Mon/Wed/ Friday for 6 doses in total
**Consolidation**<br>Peg Asparaginase 1000 units/m^2 IM on day 2 of week 6, 4 hrs after end of MTX infusion	**Consolidation**<br>Erwinase 20,000 units/m^2 IM on day 2 of week 6 4 hrs after end of MTX infusion and then Mon/Wed/ Friday for 6 doses in total
**Intensification week 9, 10 and 12**<br>Erwinase 10,000 units/m^2 IM on day 2 and day 4 week 9 and on day 2 and day 4 week 10<br>Erwinase 10,000 units/m^2 IM on day 1 (week 12) 4 hours post MTX	**Intensification week 9 , 10 and 12**<br>Erwinase 20,000 units/m^2 IM on day 2 and day 4 week 9 and on day 2 and day 4 week 10<br>Erwinase 20,000 units/m^2 IM on day 1 (week 12), 4 hours post MTX

NOT FOR CLINICAL USE

Appendix 8 : Asparaginase Study

Aims

(1) Development of a rapid high throughput assay to accurately detect AEP expression at diagnosis in blast cells, bone marrow and peripheral blood plasma.

(2) Correlation of AEP levels with asparaginase levels and the formation of anti-asparaginase antibodies.

(3) Identify antigenic epitopes responsible for formation of antibodies to asparaginase.

Samples

Please send 5 mls of marrow and/or peripheral blood in EDTA. All samples are collected at routine sampling times required by the protocol. No additional invasive tests are required.

At diagnosis [of relapse]:

1. 5 mls of bone marrow aspirate collected from a separate aspirate site to the MRD sample.
2. 5 mls of peripheral blood

All subsequent samples (peripheral blood only) are for asparaginase activity and antibody detection. For this we need to know the date the last dose of asparaginase was given and the date the sample was taken. Please ensure that the peripheral blood sample is taken at least 7 days and not more than 14 days after the last dose of asparaginase. Please fill the attached form and send with each sample, notifying if there is hypersensitivity to L-asparaginase.

During Induction [Phase 1]

(1) 5 mls of peripheral blood on Day 3, Week 2 (along with Vincristine).

(2) 5 mls of peripheral blood on Day 3, Week 4 (along with the 4th Vincristine)

During Consolidation [Phase 2]

(3) 5 mls of peripheral blood on Day 5, Week 7 (along with last dose of Cyclophosphamide and Etoposide)

During Intensification [Phase 3]

(4) 5 mls of peripheral blood on Day 1, Week 12 (along with IT Methotrexate)

During Maintenance [Phase 6]

10mls of blood along with the first intrathecal methotrexate. Please note, this sample will also be used for epitope analyses and therefore requires an adequate number of white cells. Please ensure that WCC >1.5 x 10^9/L. If not, this sample may be sent at a later date.

NOT FOR CLINICAL USE

Contact Details

All samples need to be sent by courier. The courier is CitySprint and they can be contacted on 0207 880 1347. The reference for the study is "AEP". Please send the samples to Centre for Integrated Genomic Research (CIGMR), Stopford Building, Oxford Road, Manchester M13 9PT. Tel: 0161 275 5698. If there are any queries, please contact Carly Leighton on 0161 446 3093.

Please keep the form (on the next page) in the patient's notes. Once a sample has been collected, fill in the details, photocopy the form and send it along with the sample.

NOT FOR CLINICAL USE

Asparaginase and AEP Study Form ALLR3

Please keep the master copy with the patient's notes. With each sample, please send a photocopy of the updated sample sheet and notify if there has been an allergic reaction to L-Asparaginase.

Initials: Trial No:
Centre: Date of Diagnosis:

Sample Check list – Please tick sample sent

Relapse Diagnosis

5 mls bone marrow aspirate [] Date collected:
5 mls peripheral blood []

All subsequent samples are for asparaginase activity and antibody detection. For this we need to know the date the last dose of asparaginase was given and the date the sample was taken. Please ensure that the peripheral blood sample is taken at least 7 days and not more than 14 days after the last dose of asparaginase. Please collect 5 mls of peripheral blood on:

Induction [Phase 1]
Day 3, Week 2 [] Date collected:
Date of last Asparaginase:
Day 3, Week 4 [] Date collected:
Date of last Asparaginase:

Consolidation [Phase 2]
Day 5, Week 7 [] Date collected:
Date of last Asparaginase:

Intensification [Phase 3]
Day 1, Week 12 [] Date collected:
Date of last Asparaginase

Maintenance [Phase 6]
With intrathecal MTX [] Date collected:

(Note for this sample alone, peripheral white cell count needs to be >1.5 x 10^9/l)

NOT FOR CLINICAL USE

8a. **Background**

No new drugs have entered into routine clinical practice for childhood ALL for almost 4 decades. The impressive improvement in survival during this period has been achieved by optimising therapy with the drugs we have at our disposal. During the induction and intensification period we have perhaps achieved maximum efficiency in the use of the drugs vincristine, steroids and anthracyclines. Further optimisation with L-Asparaginase remains possible as is illustrated by the introduction of PEG-Asparaginase in ALL2003.

We have identified a protease (AEP) produced by lymphoblasts that degrades both native and pegylated E Coli Asparaginase. Patients whose blast cells produce excess amouths of this protease may either fail to achieve sufficient asparagine depletion and/or develop anti-asparaginase antibodies. The study aims to correlate the levels of protease present at diagnosis with subsequent asparaginase activity and formation of antibody. If successful, we will be able to identify patients who will develop antibodies/hypersensitivity to asparaginase at diagnosis and prior to their receiving asparaginase. Such patients may benefit from Erwinase, or alternative asparaginases that are being developed.

8b.Methodology

We have developed a real-time quantitative PCR (RQ-RTPCR) assay for AEP. Asparaginase hypersensitivity has been observed only in children with high AEP levels. These are single centre observations and need to be validated on a larger cohort of patients.

The majority of patients with high AEP levels (as analysed by blast RNA levels) however do not appear to develop hypersensitivity to asparaginase. There are two possible explanations, which may be linked to each other. The enzymatic degradation of asparaginase is more likely to occur in an extracellular environment, as there is no evidence to suggest that asparaginase is able to enter the cell. We have evidence that AEP protein and RNA is present in the plasma. While the latter is the result of lysis of blasts, the former could also be a result of lysosomal discharge of contents into the plasma. Thus AEP protein levels, either in blast cells or peripheral blood/bone marrow plasma, may be more predictive of asparaginase inactivation. A second explanation is that as the amount of asparaginase is lower in the pegylated version (compared to the native), silent antibodies rather than hypersensitivity may pose a larger problem in UK based ALL protocols. Thus we will need to look not only at asparaginase activity but also antibody formation. We will also use the opportunity to see if we can identify the antigenic epitopes in E Coli Asparaginase that contribute to its immunogenicity. The information obtained from these investigations may allow us to design a less immunogenic recombinant enzyme with a longer half-life.

AEP expression will be assayed using RQ-RTPCR and ELISA in diagnostic blast cells, bone marrow and peripheral blood plasma. Asparaginase activity and antibody assay will also be performed on the baseline peripheral blood sample.

At subsequent time points during induction, delayed intensification and Capizzi blocks, assays for asparaginase activity and antibody formation will be performed. From the sample obtained during maintenance, epitope recognition studies and antibody assays will be carried out.

NOT FOR CLINICAL USE

8c. Sample Size: We aim to recruit 400 patients a year, or over the study period we will have recruited at least 1000 patients. We aim to use the first half of the data to obtain the best cut off point, and the second half to estimate the sensitivity and specificity at that cut off point for the assay(s). The predictive sample size is dependent on the sensitivity of the assay(s) and we have calculated the width of the confidence interval for four different plausible values of prevalence of antibody for our population, assuming that 500 patients are used to estimate the sensitivity and specificity once the optimal cut point has been selected (see Table below).

95% Confidence Intervals for sensitivity, for varying prevalence and sensitivity point estimates

		**Sensitivity point estimate**			
		**75%**	**80%**	**85%**	**90%**
**Prevalence**	**10%**	(62%, 87%)	(66%, 90%)	(73%, 94%)	(78%, 97%)
	**15%**	(63%, 84%)	(69%, 88%)	(75%, 92%)	(82%, 96%)
	**20%**	(65%, 83%)	(71%, 87%)	(76%, 91%)	(82%, 95%)
	**25%**	(67%, 82%)	(72%, 87%)	(77%, 91%)	(84%, 95%)

8d. Laboratory Assays

RQ-RTPCR: We have standardised the use of the TaqMan 384 well microfluidic card for the RQ-RTPCR estimation of AEP expression. High and low expressing cell lines have been used to generate a standard curve and we will use 2 house keeping genes to generate $\delta\delta C_T$ values.

ELISA**:** We have obtained both polyclonal (commercial) and monoclonal antibodies specific for AEP as well as purified recombinant AEP protein. There appears to be a good correlation between levels of AEP RNA and protein (as detected by western analyses). We have developed a sandwich ELISA, using a combination of mouse and rabbit antibodies. At the moment we are comparing the sensitivity of a HRP conjugated with the use of a biotinylated secondary antibody.

Asparaginase Activity: The diagnostic sample will serve as baseline, and subsequent samples are to be taken 7-14 days after the last L-Asparaginase dose. The quantification of enzymatic activity of all forms of L-Asparaginase is based on the measurement of substrate turnover at a maximum rate. One unit of activity is defined as the amount of enzyme which releases 1mmol of ammonia and aspartate from 1mmol asparagine per minute at 37°C. The liberated ammonia can be measured spectrophotometrically either after nesslerization or by an enzyme-coupled reaction. Thus, a number of assays have been developed. In brief, asparaginase hydrolyses AHA to aspartate and hydroxylamine. Asparaginase activity in serum samples is quantified by incubating the samples with an excess amount of L-aspartic acid β-hydroxamate (AHA) at 37°C. Asparaginase hydrolyses AHA to aspartic acid and hydroxylamine, which is detected at 710 nm after condensation with 8-hydroxychinoline and oxidation to indooxine. This method allows the quantification of 2.5 U/L Asparaginase in 20 μl serum with coefficients of variations for intra- and interday reproducibility between 1.98 - 8.77 % and 1.73 - 11.0 %, respectively, and an overall recovery of 101 +/- 9.92 %. Peripheral blood samples will be collected 5-7 days after PEG-Asparaginase and assayed for activity

Asparaginase Antibody Assay: Briefly, microtitre plates are coated with purified (and recombinant) E coli asparaginase. The positive anti-asparaginase antibody controls,

NOT FOR CLINICAL USE

calibrators with defined anti-asparaginase reactivities, normal human serum as negative control, and patient serum samples at certain dilutions are added and incubated for 1 h. After washing, a polyclonal goat anti-human IgG and IgM horseradish peroxidase conjugate is added and incubated for 1 h. After washing, 3,3,5,5-tetramethylbenzidine is added and incubated for 30 min. Anti-asparaginase antibody levels are measured at 450 nm for the enzymatic product (subtracting the absorbance at about 630 nm for nonspecific absorbance) using a microplate reader. The OD values of the calibrators are plotted against their corresponding concentration, given as arbitrary units per ml, to construct a calibration curve over the whole measuring range of the assay. Positive reactivity in serum is calculated using this calibration curve.

Antigen presentation: Peripheral blood mononuclear cells (PBMC's) will be obtained from the sample taken prior to starting maintenance therapy as well as from controls not exposed to Asparaginase. These will be examined to identify T-cell clones specific for Asparaginase processing, to identify whether the AEP cleaved fragments are recognised and if AEP processing accelerates antigen presentation. The antigens used will be AEP digested L-Asparaginase fragments and synthesised peptides. Briefly, proliferation will be measured by 3H-thymidine incorporation after 2-5 days of culture. T cells from responding PBMC will be expanded with IL-2 and individual peptide specific responses will be assayed using a set of peptides covering the L-asparaginase sequence (15mers, overlapping by 10 residues). Autologous PBMC or EBV-transformed B cells will be used as antigen presenting cells. Additionally, T cells recognising specific asparaginase peptides will be cloned from these cultures by standard methods.

NOT FOR CLINICAL USE

APPENDIX 9: GUIDELINES FOR THE INVESTIGATION AND MANAGEMENT OF THROMBO-EMBOLIC EVENTS

Background

Thrombosis is a recognised complication of the treatment and management of ALL. The true prevalence in this patient population is unknown and varies with the method of assessment. The PARKAA study (Cancer 2003, Vol.97, 2) reported a prevalence of symptomatic events of 5% and asymptomatic events of 36.5% in children undergoing induction chemotherapy for ALL with a central venous catheter insitu. Asymptomatic events were diagnosed by screening with bilateral venography or MRI. Whilst the authors recommend carefully designed clinical trials of primary prophylaxis for the prevention of TE's in this patient population, there is at present insufficient data from children treated on UK protocols to support this. However, the need to collect such information to judge the appropriateness of prophylaxis is recognised. In general, primary prophylaxis for children with CVL cannot be recommended at this time, because there is no evidence for the efficacy or safety of this approach.

Asparaginase therapy and the presence of a central venous catheter are accepted as the main predisposing factors. The literature on the role of inherited thrombophilia in predisposing to thrombotic events in these children is conflicting, with the BFM reporting a significant association, and the Canadians and other major groups, no association. It is, therefore, considered premature to recommend universal screening or primary prophylaxis, although it may be prudent to try to identify less common high-risk abnormalities eg AT deficiency, PC deficiency.

Screening

1. Universal thrombophilia screening is not recommended.
2. A careful family history of thrombosis should be taken. Children with a first or second degree relative with Protein C or A.T. deficiency should be screened for the relevant deficiency, as prophylaxis may be indicated. The literature is inconclusive for risk conferred by other inherited thrombophilias.
3. Teenage girls on the combined oral contraceptive pill should stop the pill and change to a low dose progesterone only preparation, or norethisterone.

Catheter (CVL) Related Thrombosis

Loss of CVL patency

Inability to withdraw blood with or without inability or impaired ability to infuse. If there is no evidence to suggest displacement of the catheter tip and there are no signs to suggest the presence of an occlusive thrombus, proceed with urokinase lock:

Urokinase 2,500 iu each lumen for 2-4 hours.

Failure to restore patency or recurrent loss of patency

Perform a chest roentgenogram to check the position of the catheter tip. A linogram will be required to assess CVL patency. If the linogram demonstrates the presence of a fibrin sheath with no evidence of significant clot formation around the tip, proceed to urokinase infusion.

NOT FOR CLINICAL USE

Urokinase 150 iu/kg/hr via each lumen for 12-24 hours.
Monitor coagulation prior to and every 8 hours during infusion

Low dose t-PA(0.1mg/kg/hr for 4 – 6 hrs) may be considered as an alternative thrombolytic agent

Repeat linogram following infusion to confirm resolution. If the linogram demonstrates (or is suspicious of) the presence of significant clot formation around the catheter tip, or of vessel thrombosis, proceed with further imaging studies.

Imaging: consider doppler or MR venography.

Linograms have been shown to be relatively insensitive for the detection of large vessel CVL related thrombosis. In the presence of persistent line dysfunction despite a normal linogram, further imaging is indicated.

Doppler is a sensitive technique for imaging jugular veins, but has poor sensitivity for central intrathoracic veins. MRV is less well evaluated but is likely to provide good sensitivity. Some children are likely to require a GA for this technique.

NOT FOR CLINICAL USE

Problem:

**1. Doppler or Venography confirms the presence of large vessel thrombosis**	**2. Clinical symptoms/signs of CVL related thrombosis**<br>Arrange imaging to confirm the presence and extent of thrombosis Imaging : Doppler, venography or MR venography

Treatment:

If the CVL is no longer required or is non-functioning it should be removed. If CVL access is required and the CVL is still functioning then the CVL can remain in situ. Unless otherwise contraindicated, anticoagulant therapy should be commenced. Low molecular weight heparin (LMWH) is probably the anticoagulant of choice for initial therapy in most cases.

LMWH dosing: Enoxaparin (Clexane) 1mg/kg/bd by s/c injection. Monitor using anti-Xa levels taken at 4 hours post dose, therapeutic range 0.5-1.0 iu/ml. (LMWH pharmacokinetics for children have only been established for enoxaparin and reviparin [not available in the UK] but the use of other LMWH may be acceptable with monitoring. For pulmonary thrombus, dalteparin has been recommended.

Prior to a lumbar puncture, or any other invasive procedure, the preceding two doses of LMWH should be omitted.

If there is an occlusive thrombus in a major vessel e.g. IVC, consider local thrombolytic therapy prior to anticoagulation and/or catheter removal. Low-dose t-PA (0.1mg/kg/hr) may be administered locally via the CVL but higher doses (0.5mg/kg/hr) are required for systemic therapy. t-PA should be administered for 4 – 6 hours, followed by re-imaging.

Following the initial 3 months of therapy for children with a first CVL-related DVT, prophylactic doses of oral anticoagulants (INR 1.5 to 1.8) or LMWH –(anti-factor Xa levels of 0.1-0.3) is an option until the CVL is removed. Children with recurrent CVL related DVT should have prophylactic anticoagulation until the removal of the CVL.

Some children will be scheduled to receive Asparaginase as per protocol having had an earlier catheter-related thrombotic event. Consideration should be given to removal of the CVL but those children receiving Asparaginase with a CVL in-situ should receive prophylactic anticoagulation for the duration of their Asparaginase therapy.

Thrombophilia screening should be performed following completion of anticoagulant therapy and should include Protein C, Protein S, AT, FV Leiden, lupus screen, anticardiolipin antibodies and prothrombin gene 20210A.

NOT FOR CLINICAL USE

CNS Thrombosis

Use of anticoagulants for treatment of the acute phase is contentious. Asparaginase should be suspended from that particular course but can be given in subsequent courses under prophylactic anticoagulant cover as described above.

NOT FOR CLINICAL USE

Appendix 10 : Drug Details

Drug Details

Risks of Chemotherapy for females of child bearing potential
All chemotherapy agents can cause harm to the fetus and may be excreted in breast milk. Therefore, females of child bearing potential should be counselled about this risk and asked to adopt appropriate measures to prevent pregnancy whilst on treatment with this protocol.

NB: Information here is for general use only. All drug information obtained from Manufacturer's information sheets. Some variations between local policy and actual products used may vary. Please check most recent information relevant to the product you are using.

L-Asparaginase

Erwinia L-Asparaginase (Erwinase) – Only for use in patients with allergy to E-coli Asparaginase

Note: dose of 20,000 units/m² given IM, NOT SUBCUTANEOUSLY (s/c administration gives too much variation in distribution and elimination)

Formulation	OPi : Crisantaspase (Aparaginase from *Erwinia chrysanthemi; Erwinia L-asparaginase*)10,000 units per vial as freeze dried powder for reconstitution
Storage	At 2-8°C in refrigerator
Reconstitution	Add 1-2 ml of sodium chloride solution for injection BP. Care must be taken to avoid contact with the rubber cap during mixing. Reject if not clear.
Stability	Administer within 15 minutes of reconstitution or within 8 hours in a syringe, if aseptically prepared. (Stable for up to 8 hours if in glass or polypropylene syringe. The polypropylene syringes must be latex free or the asparaginase will form a gel-like polymer.)
Administration	By intramuscular injection.
Toxicity	Hypersensitivity including Anaphylaxis. Patients with systemic reactions should not be re-exposed to Erwinase.<br>Coagulopathy<br>Acute Pancreatitis<br>Hyperglycaemia<br>Liver dysfunction
Precautions	The drug is given even if there is thrombocytopenia. Platelets may be necessary to cover the injection, but if it is given with extra local pressure they may not be needed.

PEG Asparaginase (MEDAC - Oncospar)

Note: Dose of 1, 000 units/m² (if SA > 0.6m²) given IM, NOT SUBCUTANEOUSLY.

NOT FOR CLINICAL USE

Formulation	3,750 units per 5ml vial. DO NOT SHAKE
Storage	At 2-8°C in refrigerator.
Stability	Single use only – discard any remaining solution. If aseptically prepared then 7 days stability can be given to a polypropylene syringe, store in a fridge.
Administration	By intramuscular injection. No more than 2 mls in any one site. Do not use if solution is cloudy
Toxicity	Anaphylaxis<br>Coagulopathy<br>Pancreatitis<br>Liver dysfunction
Precautions	The drug is given even if there is thrombocytopenia. Platelets may be necessary to cover the injection, but if it is given with extra local pressure they may not be needed.

MEDAC E.coli

Formulation	5000 or 10,000 units per vial as dried powder.
Storage	At room temperature
Reconstitution	Dissolve the powder (5000 u in 2 ml and 10,000 u in 4 ml) water for injection [injected into the inner wall of the vial not directly onto the powder]. The contents are dissolved by rotating the container without shaking it (avoid creating froth). The ready-to-use solution may be opalescent.
Stability	Once dissolved, can be kept at room temperature for 6hrs.
Administration	IM injection
Toxicity	Anaphylaxis<br>Coagulopathy<br>Pancreatitis<br>Liver dysfunction

Dexamethasone

Formulation	0.5 mg, 2 mg tablets. Oral solution 2mg/5ml liquid (licensed formulation)
Storage	At room temperature.
Administration	Orally daily in two divided doses as per protocol.
Toxicity	Can precipitate avascular necrosis of bone.<br>Obesity, hirsutism, fluid and salt retention, irritability.<br>Also, hypertension, diabetes and psychosis

Dexamethasone may be made up in a liquid form for those patients who cannot swallow tablets. The preparation once made up has a limited shelf life. Recommended concentrations 10mg/5ml or 2mg/5ml.

Idarubicin

Formulation	5 mg and 10 mg vials as orange-red powder for reconstitution
Storage	At 2-8°C in refrigerator

NOT FOR CLINICAL USE

Reconstitution	Reconstitute powder with water for injection then add 0.9% saline for infusion.
Stability	Depends on local policy, e.g. 7 days once reconstituted
Administration	IV infusion (as per protocol)
Toxicity	Dose reduction may be required in hepatic or renal insufficiency.<br>Cardiotoxicity – NB cumulative doses including other anthracyclines<br>Also, nausea, vomiting, alopecia and myelosuppression.
Counselling point	Discolouration of urine (red)

Mitoxantrone

Formulation	10, 12.5 and 15 ml vials at 2mg/ml concentrations available.
Storage	Store at room temperature
Reconstitution	Ready diluted – should be diluted further with at least 50ml of 0.9% saline or 5% dextrose
Stability	Depends on local policy and actual product used – varies between different manufacturers
Administration	As IV infusion (as per protocol)
Toxicity	Cardiotoxicity – NB cumulative doses<br>Also, myelosuppression, nausea, vomiting, alopecia and mucositis
Counselling point	Discolouration of urine and rarely, nails and skin (blue)

Vincristine Sulphate

Formulation	1 mg vials with 10 ml of mixing solution in combination pack. Other preparations are available.<br>1mg/ml solutions available in various vial sizes
Storage	At 2-8ºC in refrigerator.
Stability	Depends on formulation and local policy.
Administration	By bolus intravenous injection. Ensure that the needle is well into the vein and avoid extravasation. 1.5 mg/m^2, maximum dose 2 mg. Doses for children>10 years must be diluted to a minimum concentration of 0.1mg/ml unless the centre has signed a waiver with local PCT
Toxicity	Local necrosis if extravasation occurs. Jaw pain, paresis, constipation, systemic neurotoxicity and alopecia.
NB	Hepatic impairment may warrant lower doses of vincristine

Vincristine, given by the wrong route of administration, causes ascending myelitis, excruciating pain, paralysis and death, when given into the CSF. The administration and administrator need to comply with Department of Health (DOH) intrathecal guidelines. The drug should not be drawn up or available in the same room to anyone performing a lumbar puncture.

NOT FOR CLINICAL USE

Methotrexate

a) Injection (YELLOW LIQUID)

Formulation	Ready mixed vials in the following strengths:<br>2.5mg in 1ml, 25mg/ml, 100mg/ml in various vial sizes

NB Check label carefully before administration. These vials contain sodium chloride and sodium hydroxide adjusted to a pH of 8.5; there is no preservative present.

Stability	Note expiry date on bottle. After the vial has been used, discard remaining contents as there is no preservative. Stability once made up varies according to local policy – eg, 7 days if stored in refrigerator 2-8ºC
Administration	As per protocol. 100mg/ml strength is hypertonic – dilute before administration. Administer intrathecal doses at a concentration of not more than 2.5mg/ml
Toxicity	Neurotoxicity, mucositis, liver dysfunction, bone marrow depression, alopecia
Precautions	For high dose methotrexate ensure no pre-existing hepatotoxicity or severe renal failure.

b) Methotrexate for oral use

Formulation	2.5 mg and 10 mg scored tablets. 10mg/5ml suspension
Storage	At room temperature in a dark place.
Stability	Please note the expiry date.
Administration	According to protocol, one hour after food. Omit dose in the week of IT methotrexate.
Toxicity	As for the injectable form.

NB Methotrexate may be made up in liquid form to be taken orally for those patients unable to swallow the tablets. Please check on availability in your region. The preparation once made up has a limited shelf life. Recommended concentration is 10mg in 5ml.

Mercaptopurine

Formulation	10 and 50 mg scored tablets. 100mg/5ml suspension
Storage	At room temperature
Stability	Please note expiry date
Administration	75 mg/m² (titratable) during maintenance. Doses to be taken once a day one hour after food in the evening.
Toxicity	Bone marrow depression, liver dysfunction

NB Do not give allopurinol when the patient is on mercaptopurine as allopurinol blocks the major catabolic pathway of mercaptopurine. Mercaptopurine may be made up in a liquid form for those patients who cannot swallow tablets. The preparation once made up has a limited shelf life.

Counselling point	May get tarry stools

Thioguanine

NOT FOR CLINICAL USE

Formulation	40 mg scored tablets and 10mg capsules on special order only
Storage	At room temperature.
Administration	40 mg/m²/day orally on d43-49 of phase V. Doses to be taken once a day one hour after food in the evening.
Toxicity	Bone marrow suppression, stomatitis, severe diarrhoea, hepatic toxicity, loss of vibration sense and unsteady gait.

NB Thioguanine may be made up in a liquid form for those patients who cannot swallow tablets. The preparation once made up has a limited shelf life.

Co-Trimoxazole

Formulation	Paediatric suspension: 40 mg trimethoprim + 200 mg sulphamethoxazole BP in each 5 ml.
Tablets:	80 mg trimethoprim BP and 400 mg sulphamethoxazole.
Storage	At room temperature.
Stability	Please note expiry date.
Administration	Please see Appendix 6
Toxicity	Marrow depression in some cases. Hypersensitivity to sulphonamide.

Cytarabine

Formulation	Ready diluted vials, containing 100mg/ ml. Also available as a 20mg/ml (100mg/5ml) which is used for low dose boluses.
Storage	At room temperature.
Reconstitution	With supplied dilutent as recommended. Can further dilute with 0.9% saline for infusions
Stability	Please note expiry date. 48 hrs at room temperature
Administration	By direct IV injection (slow bolus). [IV or s/c in interim maintenance]. Up to 3000mg/m² IV [3hr infusion] every 12 hours.
Toxicity	Myelosuppression, alopecia, nausea, vomiting, oral ulceration, fever and arthralgia.

N.B The concentrated 100mg/ml solution carries a warning that it must not be used for direct injection, but must be diluted first.

Cyclophosphamide

Formulation	200mg, 500 mg and 1 G vials for reconstitution.
Storage	At room temperature.
Reconstitution	Add WFI then further dilute with 0.9% saline
Stability	Depends on local policy e.g. 7 days in fridge once made up
Administration	IV bolus or infusion over 30 minutes followed by 4 hours of IV hydration.
Toxicity	Myelosuppression, alopecia, cystitis, vomiting.
Precautions	Ensure adequate hydration of patient

Etoposide

Formulation	20mg/ml solution

NOT FOR CLINICAL USE

Storage	At room temperature – even when diluted
Reconstitution	Ready mixed, but needs further dilution with 0.9% saline. Ideal final concentration should be 0.2 – 0.4mg/ml
Administration	IV infusion over 2-4 hours.
Toxicity	Hypotension if infused too quickly, alopecia, myelosuppression
Precautions	Always check before and during infusion that the drug has not precipitated

Daunoxome

(Liposomal daunorubicin)

Formulation	Solution of variable concentration (due to nature of liposomes)
Storage	At 2-8 ºC in refrigerator
Reconstitution	Dilute solution further with 5% dextrose – recommended final concentration is between 0.2 – 1mg/ml
Stability	6 hours shelf life once prepared – may be extended up to 24 hours.
Administration	IV infusion over 2 hours
Toxicity	Myelosuppression, neutropenia, nausea, fatigue, headache, cough, dyspnea. Cardiomyopathy and CHF associated with daunorubicin in general

Fludarabine

Formulation	50mg vials as powder
Storage	At room temperature
Reconstitution	Add 2ml water for injection to reconstitute then can dilute with 0.9% saline for IV infusion
Stability	7 days stability at 1mg/ml
Administration	IV infusion
Toxicity	Myelosuppression (leukopenia, thrombocytopenia) oedema, fatigue, chills fever, rash

NB. Up to 8 hour shelf life once made. Patients receiving/ who have received fludarabine must always have irradiated blood products due to risk of transfusion associated GvHD
Anti-fungal prophylaxis is mandatory

Folinic acid (Leucovorin)

Formulation	Lyophilised powder for reconstitution for injection (15 or 30mg folinic acid equivalent) or 10mg/ml solution or 3mg/ml solution. Oral dose 15 mg tablets (dose to be rounded to nearest 7.5 mg)
Storage	At room temperature or in a refrigerator, 2-8ºC if 3ml/ml solution. Tablets can be stored at room temperature
Reconstitution	Add 3ml water for injection to powdered form. Can further dilute with 0.9% saline or 5% glucose if needed for IV infusion. Check manufacturer's instructions for full details
Stability	Use as soon as possible after reconstitution, up to 24 hours once made up if stored in a refrigerator. The 3mg/ml needs to be used immediately and protected from light.
Administration	IV bolus or IV infusion or oral tablets
Toxicity	Rarely, anaphylaxis or pyrexia

NOT FOR CLINICAL USE

Prednisolone

To be used only for patients who experience serious dexamethasone toxicity.
Please specify clearly on the prescription charts that prednisolone and not prednisone is to be used and that tablets must **not** be enteric coated.

Formulation	1 mg and 5 mg (scored ordinary and scored soluble) tablets available.
Dose	40 mg/m^2 po daily in place of dexamethasone at 6mg/m^2
Storage	At room temperature.
Administration	40 mg/m^2 orally daily in two divided doses, with or after food.
Toxicity	Obesity, hirsutism, fluid and salt retention, hypertension, irritability, glycosuria, avascular necrosis of bone and hyperglycaemia.

Ambisome

Formulation	50mg vials for reconstitution
Reconstitution	Use 12ml water for injection. Dilute further (using glucose infusion fluid) via 5 micron filter.
Stability	Infuse immediately after dilution. Protect from light. Incompatible with sodium chloride solutions
Storage	At room temperature
Administration	As per protocol guidelines (see Antifungal Prophylaxis Appendix 1). Depends also on local policy. Normally I.V. infusion over minimum of 30-60 mins.
Toxicity	Fever, chills, headache, nausea & vomitting, stomach pain, loss of appetite, weight loss, or muscle or joint pain, diarrhoea, decreased urination, unusual tiredness or weakness.

Itraconazole

Formulation	10mg/ml liquid only. Do not prescribe/use capsules
Stability	Please note expiry date
Storage	At room temperature
Administration	2.5mg/kg per dose twice daily
Toxicity	Nausea, abdominal pain, dyspepsia, constipation, headache, raised liver enzymes, hepatitis and cholestatic jaundice (esp if treatment exceeds 1 month), allergic reactions. Monitoring of levels required.

Voriconazole

Formulation	50 & 200mg film coated tablets. IV – 200mg powder vials for reconstitution
Stability	Please note expiry date
Reconstitution	Compatible with Glucose 5% and Sodium Chloride 0.9%<br>Must be further diluted to a final concentration of 2-5mg/ml
Administration	Infuse over 1-2 hrs

NOT FOR CLINICAL USE

Toxicity	Vision changes including blurring/photophobia, fever, headache, rash, haematological changes eg thrombocytopenia/anaemia, elevated LFTs

NOT FOR CLINICAL USE

Appendix 11: Determining weight for dosage calculations

All children should be weighed in underclothes and scales should be calibrated regularly. To ensure that children are treated effectively, without overdosing due to treatment related fat deposition, the Body mass index (BMI) should be checked at diagnosis and prior to each phase of treatment.

Calculate using the formula BMI= weight(kg)/Height ^{2} (m x m)

The BMI can then be compared to the standard Child Growth foundation BMI charts for the appropriate sex.

For children with a BMI that falls within the 2nd-98th percentiles, dose by **actual weight** using the CCLG weight/surface area (SA) charts to determine the surface area (SA) for dose calculation. These weights should be taken

-At diagnosis
-Immediately prior to each phase
-At the time of the intrathecal injection on each maintenance cycle

For children who have a BMI > 98th percentile read off the BMI at 98th percentile for their age. Calculate the **dosing weight** using the formula

Dosing weight (kg) = BMI x Ht^2 (m x m)

Use the CCLG Wt/SA charts to determine the SA for dose calculation.

For children < 2nd percentile, repeat as above reading the BMI at the 2nd percentile for calculation.

For children who have considerable weight loss due to illness during treatment it may be necessary to re- assess their BMI and dosing weight prior to recommencing treatment.

Rationale for frequency of weighing for determination of changes in drug dosage

A normal child aged 2-10 years gains only 2-6kg/year[1]. This equates to an average of 1kg every 3 months (maximum 1.5kg).

If children are weighed fully clothed rather than in underclothes, the extra weight will be greater than the quarterly change in weight. Scales, even regularly calibrated, also carry a measurement error. These errors, coupled with weight variation due to physical, endocrine, nutritional and psychosocial effects make repeated measurements inaccurate.[2]

Children having repeat doses of steroids will have greater than average weight gain, but this is primarily deposited as fat- particularly truncal fat.[3] Unless drug treatment is with very lipophilic drugs the weight gain should not affect the therapeutic doses required to treat ALL. Only anthracyclines and vinca alkaloids have longer T1/2 and larger volume of distribution, indicating major transfer to body compartments other than the blood. Both

NOT FOR CLINICAL USE

anthracyclines are ionic compounds and they and their active metabolites are highly protein bound, which would suggest less deposit into fat than into lean mass. Vincristine is highly water soluble and readily distributed into tissues. As it is predominantly renally excreted it is again unlikely that it is highly deposited in fat.[4]

Do not adjust doses on the basis of weight change any more frequently than each phase.

The CCLG Surface area charts have been validated for children with an understanding that they are less accurate at the extremes of obesity and underweight. There is no defined cut off. There is no defined BMI for obesity in children, but children with BMI > 98th percentile for age have been shown to have an increased risk of related morbidity in adult life[5]. The Child growth foundation BMI charts for boys and girls mark the 9th and 98th percentiles so it would logical to use these cut off points

Doses have traditionally been based on total weight rather than lean weight, so to change to lean body weight dosing could result in under-treatment. Dexamethasone is likely to cause greater weight gain than prednisolone.[1] It would therefore seem prudent to have a defined maximum BMI percentile, for dosing; weight percentiles take no account of tall or small stature children.

References

1. Clinical Paediatric endocrinology. Brook and Hindmarsh.

2. Short term growth; rhythms, chaos or noise? Wales J et al, Archives Dis Child 1994 71: 84-89.

3. Bodyweight change as an adverse effect of drug treatment – Mechanisms and management, Pijl H et al Drug Safety 1996, 14(5); 329-42.

4. Micromedex Drug database September 2004.

5. Use of weight for height indices in children to predict adult overweight: the Bogalusa Heart Study, Int J Obesity 1996 21:715-21.

NOT FOR CLINICAL USE

Appendix 12 : Drug interactions with cytotoxic agents

NOT FOR CLINICAL USE

Drug	Potential Interaction	Comments
Vincristine	Itraconazole	Causes inhibition of the cytochrome P450 3A4 enzyme system resulting in increased incidence of peripheral neuropathy[1]<br>Hyponatraemia associated with SIADH has been reported with concomittant use of vincristine and azole anti-fungal agents.[2]
	+ /- Nifedipine	Nifedipine has been reported to enhance these effects.
	Fluconazole/voriconazole	Fluconazole/voriconazole are not reported to have the same adverse effects although could be predicted to have similar adverse effects.
Anti-convulsants including phenytoin, carbamazepine, phenobarbitone	Possible reduced chemotherapy efficacy	An association between increased risk of relapse in children with B-lineage ALL and concomittant treatment with anticonvulsant therapy has been reported (EFS harzards ration 2.67 95% CI 1.5-4.76, p=0.0009)[3]<br>Many anti-convulsants induce hepatic enzyme activity. Significantly increased clearances of methotrexate and teniposide in patients receiving anit-convulsant therapy have been reported.3<br>**Also see Phenytoin/Dexamethasone interaction**
Phenytoin	Dexamethasone	Phenytoin and dexamethasone mutually lower the efficacy of the other drug: Phenytoin increases hepatic enzyme metabolism of dexamethasone and lowered levels of phenytoin are reported with concomittant dexamethasone therapy.<br><br>**RECOMMENDATION: Avoid the use of phenytoin, carbamazepine and phenobarbitone if possible. Possible alternative anticonvulsants include gabapentin which is renally excreted and does not induce hepatic enzymes. Clonazapam, Clobazam or Valproate have no known clinically relevant interactions with cytotoxic drugs NB. Valproate hepatotoxicity is reported.**
**Other Potential Interactions which require Close Monitoring**		
**Anti-coagulants**		
Warfarin	Concurrent chemotherapy, especially 6-MP and steroids, INCREASES INR<br>Cotrimoxazole DECREASES INR	The use of low molecular weight heparin for prophylactic therapy post thrombus formation would be preferable.[4]
6-mercaptopurine	Allopurinol	Avoid concurrent use. Can cause a 5 fold increase in AUC of 6-MP.
Methotrexate	Non Steroidal Anti-Inflammatory Drugs (NSAIDs) (including COX II inhibitors)	Increase in methotrexate levels due to competition for excretory pathways<br>**RECOMMENDATION: Avoid during high dose methotrexate. NB NSAIDs have adverse effects on platelet function**
	Penicillins, Co-Amoxiclav	Penicillins reduce methotrexate excretion.<br>**RECOMMENDATION: Avoid during high dose methotrexate.**
	Tetracyclines	Increased methotrexate toxicity through displacement of methotrexate from plasma binding sites.<br>NB. Tetracyclines should be avoided in children under 7 years due to discolouration of teeth
Cytarabine	Flucytosine	Uptake of flucytosine by fungi may be inhibited by cytarabine
Cyclophosphamide	Suxamethonium	Duration and effect of neuromuscular blockade may be increased. [5]

References

1. Sathiapalan R.K., El-Solh H. Enhanced vincristine neurotoxicity from drug interactions: case report and review of the literature. Paediatric Haematology and Oncology 2001, 18: 543-6.
2. Kamaluddin M., et al Potentiation of vincristine toxicity by itraconazole in children with lymphoid malignancy. Acta Paediatrica 2001 90:1204-08
3. Relling M.V., et al Adverse effects of anticonvulsants on efficacy of chemotherapy for ALL. Lancet 2000 356 285-90
4. Martin L.A., Mehta S.D., Diminishes anti-coagulant effects of warfarin with concomitant mercaptopurine therapy. Pharmacotherapy 2003. 23(2): 260-4
5. Kosegoglu V., et al Acquired pseudocholinestrerase deficiency after high-dose cyclophosphamide, Bone Marrow Transplantation, 1999 24(12): 1367-8

Appendix 13: Drug Toxicities and Dosage Modifications

13a. Dexamethasone

Note maximum daily dose during induction is 40mgs

Hypertension: Steroid should not be reduced. Sodium restriction and antihypertensives should be employed in an effort to control hypertension.

Malignant Hypertension: Reduce dose 33%. Sodium restriction and antihypertensive drugs may also be utilised.

Hyperglycemia: Steroids should not be reduced if the patient develops clinical signs of diabetes. Insulin therapy should be employed to control the blood glucose level such that signs and symptoms are minimal.

Pancreatitis: Do not modify dose.

Psychosis: Administer half dosage of steroid.

Suspected steroid-induced myopathy: Measure CPK with isoenzymes, consider EMG studies

Avascular necrosis: Contact trial coordinators if AVN develops before continuing therapy has begun. Omit further steroids if AVN develops during maintenance.

Varicella Zoster: Steroids should be held during active infection except during Induction. They should not be given during the incubation period following exposure to varicella.

Severe dexamethasone intolerance - change to Prednisolone 40 mg/m².

NOT FOR CLINICAL USE

13b. Vincristine

(See also drug interactions – Appendix 12)

Seizures: Hold 1 dose, then reinstitute.

Severe foot drop, paresis, abdominal pain, obstipation, or ileus:
Hold dose(s); institute aggressive regimen to treat constipation (except enemas if neutropenic), if present. When symptoms abate, resume at 1.0 mg/m²; escalate to full dose as tolerated.

Jaw pain: Treat with analgesics; do not modify vincristine dose.

Hyperbilirubinemia: Withhold if total bilirubin > 40. Administer 50% of dose if total bilirubin 25 - 40. Do not alter doses for raised transaminases.

13c. Asparaginase

Anaphylaxis or anaphylactoid reactions:

PEG-asparaginase should be discontinued if the patient develops a systemic allergic reaction (urticaria, wheezing, hypotension, etc.). Investigators may substitute Erwinia Asparaginase, 20,000 u given every 48 hours for 6 doses.

Symptomatic pancreatitis :

Discontinue L-asparaginase in the presence of symptomatic pancreatitis documented by an elevated serum amylase or lipase value or ultrasonographic abnormalities. Do not give any further asparaginase of any kind if there is a prior history of asparaginase induced pancreatitis.

Hyperglycemia : Do not modify dose. Insulin can be administered for hyperglycemia.

Ketoacidosis : Hold L-Asparaginase until blood glucose can be regulated with insulin.

Coagulopathy : When significant coagulopathy occurs, withhold L-asparaginase until resolved. Coagulopathy without bleeding is not an indication to withhold L-asparaginase. Routine clotting screens are not recommended. Management of Asparaginase associated thrombosis is described in Appendix 8.

Liver Dysfunction : For increase in hepatic transaminases (SGPT/ALT or SGOT/AST) to greater than 200, obtain total bilirubin. Withhold Asparaginase if total bilirubin > 40. Administer 50% dose if total bilirubin >25 and≤40. **Do not alter dose for abnormal transaminases**.

NOT FOR CLINICAL USE

13d. Idarubicin

Each dose of idarubicin of 10 mg/m² should be tabulated as the isotoxic equivalent of 50 mg/m² of daunorubicin or Adriamycin toward the lifetime maximum of 550 mg/m² in patients with no prior cardiac irradiation. An echocardiogram should precede anthracycline therapy. Prior anthracycline exposure and the initial baseline echocardiogram obtained prior to any anthracycline exposure should be reviewed.

Cardiac re-evaluation is recommended at a cumulative exposure of 270 mg/m² and each 50 mg/m² following. If the maximum cumulative dose is achieved or the shortening fraction on ECHO decreases to < 25% or the ejection fraction decreases to < 55%, inform Trial Coordinator.

Hyperbilirubinemia If total bilirubin > 120 omit dose; if > 90 but ≤ 120 give 25% of dose. If > 50 but ≤ 90 give 50% of dose, and if ≤ 50 give full dose.

Mucositis Maintain strict oral hygiene

Note: The use of Itraconazole as an anti-fungal prophylaxis during the first 4 weeks may potentiate anthracycline toxicity.

13e. Mitoxantrone

Each dose of mitoxantrone of 10 mg/m^2 should be tabulated as the isotoxic equivalent of 50 mg/m^2 of daunorubicin or Adriamycin toward the lifetime maximum of 550 mg/m^2 in patients with no prior cardiac irradiation. An echocardiogram should precede anthracycline therapy. Prior anthracycline exposure and the initial baseline echocardiogram obtained prior to any anthracycline exposure should be reviewed.

Cardiac re-evaluation is recommended at a cumulative exposure of 270 mg/m^2 and each 50 mg/m^2 following. If the maximum cumulative dose is achieved or the shortening fraction on ECHO decreases to < 25% or the ejection fraction decreases to < 55%, inform Trial Coordinator.

Hyperbilirubinemia If total bilirubin > 120 omit dose; if > 90 but ≤ 120 give 25% of dose. If > 50 but ≤ 90 give 50% of dose, and if ≤ 50 give full dose.

Note: The use of Itraconazole as an anti-fungal prophylaxis during the first 4 weeks may potentiate anthracycline toxicity.

13f. Intrathecal Methotrexate

Any significant neurotoxic reactions not due to lumbar puncture syndrome (low opening pressure, slow CSF flow, orthostatic symptoms) should be reported.

Systemic toxicity The dosage for IT methotrexate will not be reduced for systemic toxicity (myelosuppression, mucositis, etc.).

Uric acid > 0.4, phosphorus > 2.25, or creatinine > 90 -- DURING INDUCTION ONLY: Omit intrathecal methotrexate and substitute with intrathecal Ara-C 30mg <2yrs; 50mg 2-3yrs; 70mg >3yrs.

Hydrocephalus, microcephaly or known abnormality of CSF flow – inform Trial Coordinator

Viral, bacterial, or fungal meningitis Omit until resolved.

Seizure, paresis or organic brain syndrome attributed to intrathecal methotrexate. Omit intrathecal methotrexate and substitute with intrathecal Ara-C 30mg <2yrs; 50mg 2-3yrs; 70mg >3yrs.

NOT FOR CLINICAL USE

13g. Intravenous Methotrexate

**Liver Dysfunction (Grade 3-4)**	Omit IV MTX until toxicity grade 0-2. **Check LFT's only if patient jaundiced.**
**Kidney Dysfunction (Grade 3-4)**	Omit IV MTX until grade 0 toxicity (resolved). Resume at 100% dose.

Mucositis For grade 2 stomatitis of > 3 days duration: decrease next dose by 30%. For grade 3-4 stomatitis, withhold IV MTX until resolved; give next dose at 50% of the last given dose.

Consider culturing lesions for herpes simplex if mucositis persists or recurs and treating with Aciclovir.

13h. Oral High Dose Methotrexate

**Liver Dysfunction (Grade 3-4)**	Omit oral high dose MTX
**Kidney Dysfunction (Grade 3-4)**	Omit oral high dose MTX
**Mucositis**	For grade 3-4 stomatitis, omit oral high dose MTX

Consider culturing lesions for herpes simplex if mucositis persists or recurs and treating with Aciclovir.

13i. Cyclophosphamide

Prior history of gross haematuria or microscopic haematuria: Hydrate at 125 ml/m²/hr for 24 hours after dose and use Mesna 360 mg/m² pre, and 4, 7, 11 hours post dose.

Acute fluid retention treat with frusemide and saline; do not modify cyclophosphamide administration.

13j. High Dose Cytarabine (Ara-C)

Ara-C Syndrome	For fever, do not withhold Ara-C as this is likely with Ara-C. Obtain blood culture if central line present. For rash or conjunctivitis, withhold for grade 3-4 toxicity until resolved. If all 8 doses of Ara-C cannot be completed please report.
Liver Dysfunction	For increase in hepatic transaminases (SGPT/ALT or SGOT/AST) to greater than 200 U/L, obtain total bilirubin. Monitor SGPT/ALT or SGOT/AST and total bilirubin, before each course of Ara-C. Continue full dose therapy unless either of the following occur: 1) Bilirubin > 40;

NOT FOR CLINICAL USE

2) SGPT/ALT or SGOT/AST > 1000 on two determinations at least one week apart. If either of these occur, hold therapy with Ara-C and monitor as above, weekly. Restart at full dose therapy when the transaminase is less than 200 U/L if bilirubin is normal. Notify if the elevations persist for greater than 2 weeks. Exclude infectious hepatitis (A, B, C) for persistent (>1 month) elevations in SGPT/ALT or SGOT/AST above 200).

13k Etoposide

Kidney Dysfunction (Grade 3-4) Reduce dose to 75%

NB: For Liver Toxicity: Dosage adjustments are not required for elevated transaminase levels alone. Dosage adjustments are based on bilirubin levels, as defined for each drug. For a child with pre-existing liver disease, please speak to one of the coordinators.

13.l. Mercaptopurine

Hyperbilirubinaemia and Mucositis: As for oral methotrexate.

NOT FOR CLINICAL USE

Appendix 14: Health Economic Considerations

As a straight forward comparison, the proposed R3 is more expensive than R2 by almost £2000 per patient. This is almost entirely due to the cost of PEG-Asparaginase. However, there are potential savings which cannot be directly compared with the R2 protocol. The study rationalizes the approach to bone marrow transplantation in children with refractory or relapsed ALL and it is likely that fewer children will receive a transplant as a result of this protocol.

There are a number of biological studies being conducted to evaluate response to therapy and more importantly to understand the nature of resistance to disease and treatment outcomes. We strongly believe that the research tools being used in this study will be used in the future to design rational therapy for children with leukaemia and become part of up front protocols, achieving the dual target of increasing survival with decreasing toxicity.

NOT FOR CLINICAL USE

Appendix 15: Ethical Committees

The management of children with acute lymphoblastic leukaemia who relapse after or are refractory to front line therapy is unclear. This is because they are a heterogenous group, in whom it is difficult to prescribe a common therapeutic modality. The ALL R3 protocol, seeks to use the knowledge gained in the preceding R2 protocol to rationalise the treatment of children with relapsed or refractory disease.

The study basically wishes to define, based on minimal residual disease detection, those children who can be cured with chemotherapy alone. The study seeks to evaluate the use of minimal residual disease for risk stratification for treatment. This is based on the understanding that if disease cannot be detected at the level of 1:10,000 cells after induction, this indicates a good prognosis. Conversely, if disease is present at the level of 1:1000 cells after induction, these children do poorly even if transplanted. Children who have disease levels of less than 1:1000 but greater than 1:10,000, appear to do better after a bone marrow transplant.

Ideally, this question should be answered in a randomised fashion. However current frontline protocols are very successful (~80% survival), and the numbers relapsing are small. In order to recruit adequate numbers, patients from Australia, New Zealand and the Netherlands are being entered into the study. We have been collaborating with the International BFM group to develop a pan-European strategy. At the moment given the heterogeneity of the disease and the variance in practice in the different countries, a consensus chemotherapy protocol is not a realistic goal. However, the study design and aims are now common to both BFM and the CCLG. Therefore, the study will use identical time points, minimal residual disease detection techniques and treatment strategies to evaluate the outcome in this difficult group of patients. A yearly data share has been set up with the BFM and this data is sent to our independent Data Monitoring Committee.

A second non-randomised question is being asked. Children with high levels of disease prior to transplant will be offered an experimental cytoreduction module. First, we will evaluate the effectiveness of this schedule in decreasing the tumour load and secondly, the outcome after transplantation.

There is one randomised question being asked in ALL R3. During induction, there is a randomisation between two anthracyclines. *In vitro* assays suggest that both Idarubicin and Mitoxantrone have better activity against relapsed blast cells than Daunorubicin or Doxorubicin. Both drugs have been extensively used in the management of childhood acute leukaemias and have tolerable toxicity. The trial will investigate the use of Real-Time Polymerase Chain Reaction as a surrogate marker to evaluate the speed of response to therapy. If successful, this may be used to assess the effect of drugs in other window phase studies. The effect of the two drugs will also be analysed with regards to toxicity and survival.

ALLR3 is the first childhood ALL protocol in this country using molecular tools and asking biological questions. Material (bone marrow and peripheral blood) will be obtained at set time points for minimal residual disease detection which determines therapy. Additional material will be collected at these time points to carry out the biological studies designed to

NOT FOR CLINICAL USE

help us understand why these children relapse with the intent that the information will be used to design more specific therapy.

Parents/patients will be consented at diagnosis for storage of extra tissue and analysis. All tissue collected will be considered to be a gift and will be under the jurisdiction of the Childhood Leukaemia Working Party. The material will be sent processed and stored at the central repository of the UK DNA Banking Network (Medical Research Council) in Manchester. The samples will be processed, catalogued and stored. Access to this material will be regulated via the CLWP/CCLG Biological Committee and Leukaemia Cell Bank.

NOT FOR CLINICAL USE

Appendix 16: Patient Information Sheets and Informed Consent Forms

NOT FOR CLINICAL USE

This form needs to be on headed paper of the local hospital where treatment is being offered. Unheaded paper is not acceptable.

Information Sheet for Parents/Guardians
Version 6: August 2007

ALL R3, A Trial for Relapsed and Refractory Childhood Acute Lymphoblastic Leukaemia

Dear Parent / Guardian,
Your child has relapsed with acute lymphoblastic leukaemia or is not responding to treatment. We would like to invite your child to take part in a research study which aims not only to understand why some children with acute lymphoblastic leukaemia fail therapy but also to improve the outcome. Before you decide whether you would like your child to take part it is important for you to understand why the research is being done and what it will involve. Please take time to read the following information carefully and discuss it with others if you wish. Ask us if there is anything that is not clear or if you would like more information. Please take time in deciding whether or not you wish for your child to take part.

NOT FOR CLINICAL USE

Background
Your child has relapsed with leukaemia and will shortly be starting treatment. This treatment is different from what he/she has already received. We know that those children who relapse on treatment or are within 6 months of stopping treatment are at high risk and often require bone marrow transplantation. Others that relapse later are considered to be intermediate risk or standard risk. These definitions will be explained more clearly by your physician. For these children, we think that how quickly the disease responds to treatment this time, may help decide whether transplant or chemotherapy is a better option.

During the first phase of treatment we will be examining the effect of two drugs which have similar action and side effects, Mitoxantrone and Idarubicin. While research has shown that both these drugs are active against relapsed leukaemic cells, we do not know if one is better than the other. To monitor how your child is responding to treatment, we will be examining the marrow at frequent intervals to look for the presence of disease using very sensitive tests. If your child is intermediate or standard risk and has no or very low levels of disease after 35 days of treatment, then it is likely that he/she will do well on chemotherapy alone. For all other categories, a bone marrow transplant may also be an option.

1. Why Has My Child Been Chosen?
We will be asking the parents of all children with relapsed acute lymphoblastic leukaemia if they are willing to take part in this study. The more patients that take part, the more information we can gather that will help guide us in the treatment of children with relapsed leukaemia.

2. Does My Child Have To Take Part?
You are under no obligation to take part in these studies and are entitled to ask us to store material for your child's benefit only (ie. to confirm a diagnosis). Your doctor may wish to withdraw your child from this study if it is felt to be in their best interest. Any of these decisions will not have any influence on the treatment that your child receives and you may withdraw your child's participation at any stage without compromising your child's care.

3. What Will Happen If I My Child Does Take Part?
Your physician will explain to you whether your child falls into the standard, intermediate or high risk group. For high risk children, the best option appears to be a bone marrow transplant. First, chemotherapy will be used to control the disease. If a suitable donor is available, then your child will be offered a bone marrow transplant. For those considered to be intermediate or standard risk, we will monitor the level of disease in the bone marrow carefully using very sensitive tests. If your child has no or very low levels of disease after 35 days of treatment, then it is likely that he/she will do well on chemotherapy alone.

As we do not know whether Mitoxantrone or Idarubicin is more effective, we need to make comparisons. Your child will be put into one of two groups and then compared. The groups are selected by a computer which has no information about the individual – i.e. by chance. Children will therefore receive either Mitoxantrone or Idarubicin and their efficacy can be compared.

We will be collecting bone marrow samples at timed intervals to assess the outcome of treatment. We are asking your permission to obtain an extra sample at these time points. All samples will be collected when the routine tests are done, so there are no additional tests are necessary. Any left over sample is stored so that we may, if we need to, run other tests to confirm our results. These leftover samples can be frozen and kept for many years. We would like to ask you if we may also store some of this leftover sample to use for our research studies in the future.

4. What Projects Will These Specimens Be Used For?
During the study itself, these samples will be used to understand why certain children relapse and if there are ways we can determine better therapy. The stored samples may be used for research projects in the future. These projects are related to the treatment, diagnosis and genetics of childhood cancer. None of these projects would be for commercial gain and hence no samples would be sold or given to any commercial enterprise. Access to the stored samples will only be granted to researchers that have been recognised to be doing scientific research that will be valuable to future patients. Any research project will have to be approved by the United Kingdom Childhood Leukaemia Working Party and by an ethics committee. Should you consent, the samples will be accepted as a donation and they will be under the jurisdiction of the United Kingdom Childhood Leukaemia Working Party to use at their discretion. All rights to these samples will be waived and all information from the research will be kept by the Working Party. If you decide at any time that you do not wish to consent to excess material being stored any leftover specimens will be destroyed.

5. What Do I Have To Do?
We need your consent to be able to register your child in the study and then collect and store their treatment information for analysis.
We need your consent to be able to randomise the treatment at the start of therapy. We will also need your consent to be able to collect and store bone marrow and blood samples from your child.

6. What Are The Drugs Being Tested And What Are Their Main Side Effects?
All the chemotherapy drugs used in this protocol are well known; there are no 'experimental' drugs used. Your child will receive either Idarubicin or Mitoxantrone. Both drugs are given at 10mg/m^2, as an infusion into a vein over a 1 hour period. The drugs have similar toxicities. Idarubicin is red and Mitoxantrone blue and they can cause a temporary

NOT FOR CLINICAL USE

discolouration of urine or nails. Both drugs can make your child feel sick, suppress the bone marrow thus increasing the risk of infection and he/she will lose their hair.

Most drugs used in the treatment of childhood cancer have similar side effects and the commonest is an increased risk of infection as your child's ability to fight infections are considerably decreased during this time. He/she may also have a poor appetite and lose weight during the first weeks of therapy. Your physician will advise you about the precautions you need to take at this time and what should be done to tackle these problems.

7. What Are The Alternatives For Treatment?
This is the only protocol in the [United Kingdom/ANZCHOG/DCOG], recommended for the treatment of children with relapsed Acute Lymphoblastic Leukaemia and has been designed from our past experience and with wide consultation from other groups around the world. You may choose for your child not to be randomised for Idarubicin or Mitoxantrone and/or not consent for storage of tissue.

8. Are There Any Risks?
To our knowledge, there are no extra risks involved in receiving either Mitoxantrone or Idarubicin. There are also no extra risks involved in storing samples of blood, bone marrow or tissue for future tests or for research purposes.

9. Are There Any Benefits?
Whether you decide to take part or not, your child will receive the best possible medical care. By taking part in this study, we hope that only children who require a bone marrow transplant will receive one and thus no child will be exposed to more toxic treatment than is necessary. We also hope that the more intensive treatment may cure more children with relapsed acute lymphoblastic leukaemia. Finally, we hope that the scientific studies will shed light on why children relapse as well as improve their treatment.

10. What if new information becomes available?
Sometimes during the course of a research project, new information becomes available about the treatment. If this happens your doctor will tell you about this and discuss whether you wish to continue with the current study. If you decide to withdraw, your doctor will make arrangements for your care to continue. If you decide to continue you will be asked to sign a new consent form.

11. What Happens When The Study Stops?
The study will finish when we have enrolled enough patients to find the answers to the study questions. We anticipate it will take about seven years as relapsed leukaemia is still quite a rare condition. At the end, the data will be examined and the results will be used in the future to help format new and better treatments for children with relapsed leukaemia.

12. What If Something Goes Wrong?
The storage of extra material will not impact on your child's health. However, if your child has any serious side effects from the treatment, as with all other chemotherapy protocols, an alternative treatment plan will be devised by your consultant. If your child is harmed by taking part in this research project, there are no special compensation arrangements. If your child is harmed due to someone's negligence, then you may have grounds for a legal action but you may have to pay for it. Regardless of this, if you wish to complain, or have any concerns about any aspect of the way you have been approached or treated during the course

NOT FOR CLINICAL USE

of this study, the normal [National Health Service] complaints mechanisms should be available to you.

13. Who will have access to details about my child?
In accordance with the Data Protection Act of 1998 all information about your child and their illness will be kept strictly confidential. All data related to your participation in the ALLR3 trial for relapsed leukaemia will be stored under the care of the Cancer Research UK data register. This data will be used solely for research purposes.

Data that has been obtained as a result of your participation in ALLR3 may be used for other research projects into leukaemia and may also be given to regulatory authorities in the [UK/Australia/New Zealand/The Netherlands] such as the Office of National Statistics. Should this happen then all your child's personal details will be removed before the data is analysed so that he/she cannot be identified by name, address or GP details. Your treatment centre with your permission will let your GP know that your child is being treated on this study. Data collected from this study will be stored for a minimum of 15 years after the end of the trial. At any stage during this time the records may be audited to validate the trial data.

14. Can I find out what research is being done?
Yes. At any time in the future, you are more than welcome to ask your doctor and the researchers what studies are being undertaken and what results have been obtained.

15. What if I have any questions now or later?
If you have any questions please do not hesitate to ask any of the medical or research team at your treatment centre to answer them for you. If you have any queries regarding the storage of your data you should contact *(name of doctor at local centre, contact details)*

16. Who Is Organising This Research?
The study is being conducted by the United Kingdom Childrens Cancer and Leukaemia Group.

17. What if I have any concerns?
If you have any concerns or other questions about this study or the way it has been carried out, you should contact the (*name of doctor at local centre, contact details)*
or you may contact your local hospital complaints department.

Thank you for considering this proposal. If you agree to your child taking part, we will need you to sign a consent form. You will be given a copy of the consent form and this information sheet to keep.

NOT FOR CLINICAL USE

This form needs to be on headed paper of the local hospital where treatment is being offered. Unheaded paper is not acceptable.

Information Sheet for Child Aged 14 – 18 years
(Version 6 dated August 2007)

ALL R3, A Trial for Relapsed and Refractory Childhood Acute Lymphoblastic Leukaemia

Dear ,

You have already been treated for acute lymphoblastic leukaemia (ALL). You have now been told that your disease has come back. We would like to invite you to take part in a research study which aims not only to help us understand why the disease has returned but also to improve the chances of a cure. Please take time to read the following information carefully and discuss it with the doctors and nurses if you wish. Ask us if there is anything that is not clear or if you would like more information. Please take your time in deciding whether or not you wish to take part.

Background

Soon we will start giving you treatment again. We will have to give you different drugs from those that we gave you last time. Children who have a relapse of ALL can be divided into 3 groups - high, intermediate and standard risk. These definitions will be explained more clearly by your doctor. We think that how quickly the disease responds to treatment this time, may help decide whether transplant or chemotherapy is a better option.

During the first phase of treatment we will be examining the effect of two drugs which work in a similar way and have similar side effects. These are called Mitoxantrone and Idarubicin and both these drugs have proved to be active against relapsed leukaemic cells, but we do not know if one is better than the other. To monitor how you are responding to treatment, we will be examining your bone marrow several times to look for the presence of disease using very sensitive tests. After 35 days of treatment, depending on your bone marrow result, a decision will be made to give you either chemotherapy alone or a bone marrow transplant.

1. Why Have I Been Chosen?

We are asking all children with relapsed ALL, who meet the eligibility criteria, if they want to take part in this study. The more patients that take part in this study, the more information we can gather to help guide us in the treatment of children with relapsed leukaemia.

2. Do I Have To Take Part?

Taking part in the research is voluntary so it is up to you to decide whether or not you want to take part. If you do decide to take part you will be given this information sheet to keep and be asked to sign a consent form. If you decide to take part you are still free to withdraw at any time. Your doctor may also wish to withdraw you from this study if it is felt to be in your best interest. A decision to withdraw at any time, or a decision not to take part, will not affect the standard of care that you receive.

NOT FOR CLINICAL USE

3. What Will Happen If I Do Take Part?
Your doctor will explain to you whether you fall into the standard, intermediate or high risk group. For high risk children, the best option appears to be a bone marrow transplant. First, chemotherapy will be used to control your disease. If a suitable donor is available, then you will be offered a bone marrow transplant. If you are considered to be intermediate or standard risk, we will monitor the level of disease in the bone marrow carefully using very sensitive tests. If you have no or very low levels of disease after 35 days of treatment, then it is likely that you will do well on chemotherapy alone.

As we do not know whether Mitoxantrone or Idarubicin is more effective, we will need to make comparisons. You will be put into one of two groups and then compared with children from the other group. The groups are selected by a computer which has no information about you, therefore it is by chance. You will therefore receive either Mitoxantrone or Idarubicin so that we can identify which is most effective.

We will be collecting bone marrow samples at timed intervals to assess the success of the treatment. We are asking your permission to obtain extra samples at these time points so that no additional tests would need to be done. Any left over sample is stored, so that we may, if we need to, run other tests to confirm our results. These leftover samples can be frozen and kept for many years. We would like to ask you if we may also store some of this leftover sample to use for our ethically approved research studies in the future.

4. What Projects Will These Specimens Be Used For?
During the study itself, these samples will be used to understand why certain children relapse and if there are ways we can determine better therapy. The stored samples may be used for research projects in the future. These projects are related to the treatment, diagnosis and genetics of childhood cancer. None of these projects would be for commercial gain and hence no samples would be sold or given to any commercial enterprise. Access to the stored samples will only be granted to researchers that have been recognised to be doing scientific research that will be valuable to future patients. Any research projects will have to be approved by the United Kingdom Childrens Cancer and Leukaemia Group and by an ethics committee. Should you consent, the samples will be accepted as a donation and they will be under the jurisdiction of the United Kingdom Childrens Cancer and Leukaemia Group to use at their discretion. All rights to these samples will be waived and all information from the research will be kept by the CCLG. If you decide at any time that you do not wish to consent to excess material being stored any leftover specimens will be destroyed.

5. What Do I Have To Do?
We need your consent to register you onto the trial and also to collect and store information about your response to treatment on the trial.
We need your consent to be able to randomise the treatment at the start of therapy. We will also need your consent to be able to collect and store your bone marrow and blood samples. You may choose not be randomised for Idarubicin or Mitoxantrone and/or not consent for storage of tissue.

6. What Are The Drugs Being Tested And What Are Their Main Side Effects?
All the chemotherapy drugs used in this protocol are well known, there are no experimental drugs used. You will receive either Idarubicin or Mitoxantrone. Both drugs are given as an infusion into a vein over a 1 hour period. The drugs have similar side effects. Idarubicin is red and Mitoxantrone blue, and they can cause a temporary discolouration of urine or nails.

NOT FOR CLINICAL USE

Both drugs can make you feel sick, they will decrease your ability to fight infections and you will lose your hair.

Most drugs used in the treatment of childhood cancer have similar side effects and the commonest is an increased risk of infection as you are less able to fight infections during this time. You may also have a poor appetite and lose weight during the first weeks of therapy. Your doctor will advise you about the precautions you need to take and what should be done to tackle these problems.

7. What Are The Alternatives For Treatment?
This is the only protocol in the [United Kingdom/Australia/New Zealand/The Netherlands], recommended for the treatment of children with relapsed ALL and has been designed from our past experience and with wide consultation from other groups around the world.

8. Are There Any Risks?
To our knowledge, there are no extra risks involved in receiving either Mitoxantrone or Idarubicin. There are also no extra risks involved in storing samples of blood, bone marrow or tissue for possible tests or for research purposes.

9. Are There Any Benefits?
Whether you decide to take part or not, you will receive the best possible medical care. By taking part in this study, we hope that only children who require a bone marrow transplant will receive one, so that no child will be exposed to unnecessary treatment. We also hope that the more intensive treatment may cure more children with relapsed ALL. We hope that the information we obtain will make the treatment even better for children in the future and help us to understand why some children relapse.

10. What if new information becomes available?
Sometimes during the course of a research project, new information becomes available about the treatment. If this happens your doctor will tell you about this and discuss whether you wish to continue with the current study. If you decide to withdraw your doctor will make arrangements for your care to continue. If you decide to continue on this study, you will be asked to sign a new consent form.

11. What Happens When The Study Stops?
The study will finish when we have enrolled enough patients to find the answers to the study questions. We anticipate that it will take about seven years, as relapsed leukaemia is still quite a rare condition. At the end, the data will be examined and the results will be used in the future to help plan new and better treatments for children with relapsed leukaemia.

12. **What If Something Goes Wrong?**
The storage of extra material will not impact on your health. However, if you have any serious side effects from the treatment, as with all other chemotherapy protocols, an alternative treatment plan will be devised by your consultant. If you wish to complain, or have any concerns about any aspect of the way you have been approached or treated during the course of this study, the normal National Health Service complaints mechanisms should be available to you, just ask your doctor or nurse.

NOT FOR CLINICAL USE

13. Who will have access to details about me?

In accordance with the Data Protection Act of 1998 all information about_you and your illness will be kept strictly confidential. All data related to your participation in the ALLR3 trial for relapsed leukaemia will be stored under the care of the Cancer Research UK data register. This data will be used solely for research purposes as detailed in this information sheet. Data that has been obtained as a result of your participation in ALLR3 may be used for other research projects into leukaemia and may be given to other regulatory authorities. Should this happen then all your personal details will be removed before the data is analysed so that you cannot be identified by name, address or GP details. Your treatment centre, with your permission, will let your GP know that you are being treated on this study. The data will be stored for a minimum of 15 years after the end of the trial. At any stage during this time the records may be audited to validate the trial data

14. Can I find out what research is being done?

Yes. At any time in the future, you are more than welcome to ask your doctor and the researchers what studies are being undertaken and what results have been obtained.

15. What if I have any questions now or later?

If you have any questions please do not hesitate to ask any of the medical or research team at your treatment centre to answer them for you.

16. Who Is Organising This Research?

The study is being conducted by the United Kingdom Childrens Cancer and Leukaemia Group.

17. What if I have any concerns?

If you have any concerns or other questions about this study or the way it has been carried out, you should contact *(name of doctor at local centre, contact details)* or you may contact your local hospital complaints department.

Thank you for reading this information. If you agree to taking part, you and your parent/guardian should sign a consent form. You should be given a copy of the consent form and this information sheet to keep.

NOT FOR CLINICAL USE

(This form needs to be on headed paper of the local hospital where treatment is being offered. Unheaded paper is not acceptable.)

Information Sheet for Child Aged 8-14 years

(Version 6 dated August 2007)

ALL R3, A Trial for Relapsed and Refractory Childhood Acute Lymphoblastic Leukaemia

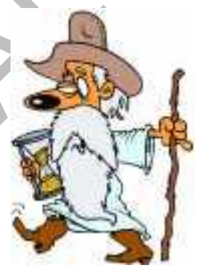

Dear ,

You have already been treated for acute lymphoblastic leukaemia, which we call 'ALL'. You have now been told that your disease has come back. We would like to invite you to take part in a research study which hopes to improve how successfully we treat your disease and also to help us understand why it came back. Please take time to read the following information carefully and discuss it with your parents or guardian, nurses and doctors if you wish. Ask us if there is anything that is not clear or if you would like more information. Please take your time in deciding whether or not you wish to take part.

NOT FOR CLINICAL USE

Background

Soon we will start giving you treatment again. This time, to make the disease go away, we will have to give you different drugs, which we call chemotherapy, from those we gave you last time. If your disease has come back quickly or if it does not respond to treatment quickly, you may need a form of treatment called a bone marrow transplant. If you do need this, we will talk to you about it first.

We would also like to store a little bit of your bone marrow whenever we need to look at it. This will be used by the scientists to understand why your disease came back and improve the way we treat your disease.

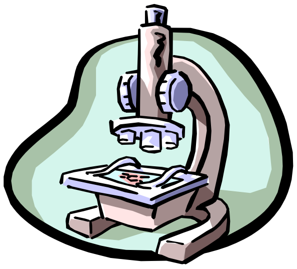

1. Why Have I Been Chosen?

We will be asking all children with relapsed ALL if they would be willing to take part in this study. The more patients that take part, the more information we can gather that will help guide us in the treatment of children with relapsed leukaemia.

2. Do I Have To Take Part?

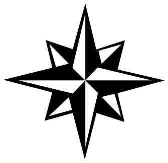

No. It is up to you and your parents or guardian to decide whether or not to take part. If you do decide to take part,

NOT FOR CLINICAL USE

you and your parents or guardian will be asked to sign a consent form. You will be given a copy of the consent form and this information sheet to keep. You can change your mind at any time and might decide not to take part after all, don't worry because your doctor will not mind at all.

3. What Will Happen If I Do Take Part?

You will receive chemotherapy for 14 weeks. For most of this time, we will have to keep you in hospital, though you will go home for a few days every now and then. We will be asking your parents or guardian if you will take part in a special study called a randomised study. We need to do this because we don't always know which is the best drug, so we need to compare one with the other. We would like to treat half the children with one drug and half with the other. During the period of chemotherapy, you will go to theatre a few times so that we can collect a sample of bone marrow from you. This will tell us if you are responding to treatment well. If you need a bone marrow transplant, this will be done after the 14 weeks of chemotherapy. If you do not need one, then you will be treated for 2 years with the chemotherapy drugs. Sometimes you will need to be given blood and platelets.

NOT FOR CLINICAL USE

4. Are There Any Other Choices?

Should you or your parents or guardian decide not to take part in this study, then your doctor will help you decide what other treatment is available.

5. What Are The Side Effects?

This is the same as last time. You may feel a bit sick, not feel like eating, have a high temperature and your hair might fall out.

6. Will There Be Any Other Problems?

No, we don't think so.

7. What Are The Benefits?

Whatever you and your parents or guardian decide, you will only get the very best treatment from your doctors and nurses. By_taking part in this study we hope you will receive the best treatment available. We hope that the information we get will make the treatment even better for children like you in the future.

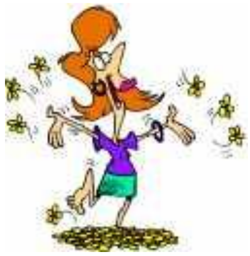

8. Will anyone else know that I am taking part in this study?

The only people who will know that you are taking part in this study will be the team of doctors and nurses looking

NOT FOR CLINICAL USE

after you and members of the United Kingdom Childrens Cancer and Leukaemia Group who collect information on all patients like yourself on this study.

9. What will happen to the results of the study?

The results of the study are looked at on a regular basis. When the study is finished, the results will be printed in a special sort of newspaper for doctors. Your name will not appear on any report.

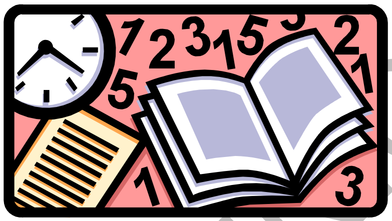

10. What if I have any questions now or later?

If you have any questions, don't be afraid to talk to somebody about them. Doctors and nurses get asked questions all the time, **so** they won't mind. This is a lot of information to take in but thank you for reading this letter.

Contact: Name of doctor at centre, contact details

NOT FOR CLINICAL USE

(This form needs to be on headed paper of the local hospital where treatment is being offered. Unheaded paper is not acceptable.)

Information Sheet for Child Aged under 8 years

(Version 6 dated August 2007)

ALL R3, A Trial for Relapsed and Refractory Childhood Acute Lymphoblastic Leukaemia

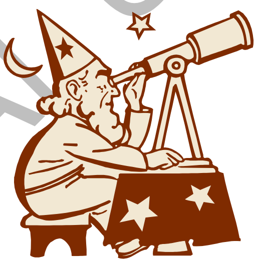

Dear ,

You have already been treated for acute lymphoblastic leukaemia, which we tend to call 'ALL'. You have now been told that your disease has come back. We would like to invite you to take part in a study which hopes to improve the way we treat your disease and also to help us understand why it came back.

Background

To make the disease go away, we will have to give you different drugs from those we gave you last time which are called chemotherapy.

NOT FOR CLINICAL USE

1. Why Have I Been Chosen?

We will be asking all children with relapsed ALL if they would be willing to take part in this study. The more people that take part, the more information we can gather that will help us in the treatment of children with relapsed leukaemia.

2. Do I Have To Take Part?

You do not have to take part in the study. If you and your parents decide to take part, you can still change your minds at any time and your doctor will not mind at all.

3. What Will Happen If I Do Take Part?

You will have chemotherapy for 14 weeks and for most of this time, we will have to keep you in hospital, although you will go home for a few days sometimes. You will go to theatre a few times so that we can collect a sample of bone marrow from you. You may also need a treatment called a transplant which is like having a blood transfusion. You will need to stay in a special_room afterwards until you are well enough to go home. If you do not need one, then you will be treated for 2 years with the chemotherapy drugs.

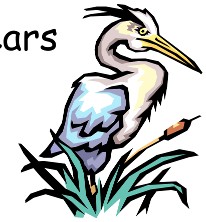

NOT FOR CLINICAL USE

4. Are There Any Other Choices?

Should you or your parents decide not to take part in this study, then your doctor will help you decide what other treatment is available.

5. What Are The Side Effects?

These might be the same as last time. You may feel a bit sick, not feel like eating, have a high temperature and your hair might fall out.

6. What Are The Benefits?

Whatever you and your parents decide, you will only get the very best treatment from your doctors and nurses. We hope that the information we get will make the treatment even better for children like you in the future.

7. Will anyone else know that I am taking part in this study?

The only people who will know that you are taking part in this study will be the team of doctors and nurses looking after you and a group of people who collect information on all the children like you in this study.

NOT FOR CLINICAL USE

8. What will happen to the results of the study?

When the study is finished, the results will be printed in a special sort of newspaper for doctors but your name will not be in it.

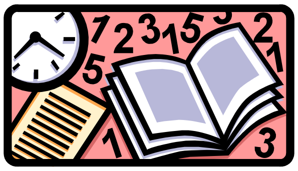

9. If I have any questions now or later?

If you have any questions, don't be afraid to talk to somebody about them. Doctors and nurses get asked questions all the time, so they won't mind.

Thank you for listening to or reading this with a grown up.

Contact : (Name of doctor at centre, contact details)

NOT FOR CLINICAL USE

(To be printed on local Hospital / Institution headed paper)

GP INFORMATION SHEET
(Version 1, August 2007)

ALLR3, An International Collaborative Trial for Relapsed and Refractory Acute Lymphoblastic Leukaemia (ALL)

Chief Investigator : Professor Vaskar Saha
Local Investigator:

Dear Dr ,

Re: Name:
D.O.B:
Hospital Number:

The above patient has relapsed with acute lymphoblastic leukaemia and has agreed to take part in the ALLR3, phase III study.

1.What is the purpose of the study?
Your patient has relapsed with leukaemia and will shortly be starting treatment. This treatment is different from what he/she has already received. We know that those children who relapse on treatment or are within 6 months of stopping treatment are at high risk and often require bone marrow transplantation. Others that relapse later are considered to be intermediate risk or standard risk. For these children, we think that how quickly the disease responds to treatment this time, may help decide whether transplant or chemotherapy is a better option.

During the first phase of treatment we will be examining the effect of two drugs which have similar action and side effects, Mitoxantrone and Idarubicin. While research has shown that both these drugs are active against relapsed leukaemic cells, we do not know if one is better than the other. To monitor how your patient is responding to treatment, we will be examining the marrow at frequent intervals to look for the presence of disease using very sensitive tests. If your patient is intermediate or standard risk and has no or very low levels of disease after 35 days of treatment, then it is likely that he/she will do well on chemotherapy alone. For all other categories, a bone marrow transplant may also be an option.

1. Why has my patient been chosen?
Your patient has relapsed with relapsed acute lymphoblastic leukaemia and fulfils the eligibility criteria for this study.

2. Does My Patient Have To Take Part?
It is up to your patient and/or their parents/guardian to decide whether or not they want to take part in this study. They will have been given information sheets and asked to sign a consent form. There is no obligation for the child to take part in this study and they are entitled to ask us to store material for their benefit only (ie. to confirm a diagnosis). Any of these decisions will not have any influence on the treatment that the

NOT FOR CLINICAL USE

patient receives and they may withdraw participation at any stage without compromising their care.

3. What Will Happen If My Patient Does Take Part?
Your patient will fall into either the standard, intermediate or high risk group. For high risk children, the best option appears to be a bone marrow transplant. First, chemotherapy will be used to control the disease. If a suitable donor is available, then your patient will be offered a bone marrow transplant. For those considered to be intermediate or standard risk, we will monitor the level of disease in the bone marrow carefully using very sensitive tests. If your patient has no or very low levels of disease after 35 days of treatment, then it is likely that he/she will do well on chemotherapy alone.

As we do not know whether Mitoxantrone or Idarubicin is more effective, we need to make comparisons. Your patient will be asked if they would like to participate in the randomisation so that the two drugs can be compared.

The study is also asking biological questions and we will be collecting bone marrow samples at timed intervals to assess the outcome of treatment. We are asking permission to obtain an extra sample at these time points. All samples will be collected when the routine tests are done, so no additional tests are necessary. Any left over sample is stored so that we may, if we need to, run other tests to confirm our results. These leftover samples can be frozen and kept for many years.

4. What Projects Will These Specimens Be Used For?
During the study itself, these samples will be used to understand why certain children relapse and if there are ways we can determine better therapy. The stored samples may be used for research projects in the future. These projects are related to the treatment, diagnosis and genetics of childhood cancer. None of these projects would be for commercial gain and hence no samples would be sold or given to any commercial enterprise. Access to the stored samples will only be granted to researchers that have been recognised to be doing scientific research that will be valuable to future patients. Any research project will have to be approved by the United Kingdom Childhood Leukaemia Working Party and by an ethics committee. Should your patient consent, the samples will be accepted as a donation and they will be under the jurisdiction of the United Kingdom Childrens Cancer and Leukaemia Group [CCLG] to use at their discretion. All rights to these samples will be waived and all information from the research will be kept by the CCLG. If your patient decides at any time that they do not wish to consent to excess material being stored any leftover specimens will be destroyed.

5. What Are The Drugs Being Tested And What Are Their Main Side Effects?
All the chemotherapy drugs used in this protocol are well known; there are no 'experimental' drugs used. Your patient will receive either Idarubicin or Mitoxantrone. Both drugs are given at 10mg/m^2, as an infusion into a vein over a 1 hour period. The drugs have similar toxicities. Idarubicin is red and Mitoxantrone blue and they can cause a temporary discolouration of urine or nails. Both drugs can make your patient feel sick, suppress the bone marrow thus increasing the risk of infection and he/she will lose their hair.

NOT FOR CLINICAL USE

Most drugs used in the treatment of childhood cancer have similar side effects and the commonest is an increased risk of infection as your patient's ability to fight infections are considerably decreased during this time. He/she may also have a poor appetite and lose weight during the first weeks of therapy.

6. What Are The Alternatives For Treatment?
This is the only protocol in the United Kingdom recommended for the treatment of children with relapsed Acute Lymphoblastic Leukaemia and has been designed from our past experience and with wide consultation from other groups around the world.

7. Are There Any Risks?
To our knowledge, there are no extra risks involved in participating in this study nor from receiving either Mitoxantrone or Idarubicin. There are also no extra risks involved in storing samples of blood, bone marrow or tissue for future tests or for research purposes.

8. Are There Any Benefits?
Whether your patient decides to take part or not, they will receive the best possible medical care. By taking part in this study, we hope that only children who require a bone marrow transplant will receive one and thus no child will be exposed to more toxic treatment than is necessary. We also hope that the more intensive treatment may cure more children with relapsed acute lymphoblastic leukaemia. Finally, we hope that the scientific studies will shed light on why children relapse as well as improve their treatment.

9. What if new information becomes available?
Sometimes during the course of a research project, new information becomes available about the treatment. If this happens the Paediatric Consultant will tell your patient about this and discuss whether they wish to continue with the current study. If they decide to withdraw, the Consultant will make arrangements for their care to continue.

10. What Happens When The Study Stops?
The study will finish when we have enrolled enough patients to find the answers to the study questions. We anticipate it will take about seven years as relapsed leukaemia is still quite a rare condition. At the end, the data will be examined and the results will be used in the future to help format new and better treatments for children with relapsed leukaemia.

11. What If Something Goes Wrong?
The storage of extra material will not impact on your child's health. However, if your child has any serious side effects from the treatment, as with all other chemotherapy protocols, an alternative treatment plan will be devised by your consultant. If your child is harmed by taking part in this research project, there are no special compensation arrangements. If your child is harmed due to someone's negligence, then you may have grounds for a legal action but you may have to pay for it. Regardless of this, if you wish to complain, or have any concerns about any aspect of the way you have been approached or treated during the course of this study, the normal [National Health Service] complaints mechanisms should be available to you.

NOT FOR CLINICAL USE

12. Who will have access to details about my patient?
In accordance with the Data Protection Act of 1998 all information about your patient and their illness will be kept strictly confidential. All data related to their participation in the ALLR3 trial for relapsed leukaemia will be stored under the care of the Cancer Research UK data register. This data will be used solely for research purposes.

Data that has been obtained as a result of their participation in ALLR3 may be used for other research projects into leukaemia and may also be given to regulatory authorities in the [UK/Australia/New Zealand/The Netherlands] such as the Office of National Statistics. Should this happen then all of their personal details will be removed before the data is analysed so that he/she cannot be identified by name, address or GP details. Data collected from this study will be stored for a minimum of 15 years after the end of the trial. At any stage during this time the records may be audited to validate the trial data.

13. Who Is Organising This Research?
The study is being conducted by the United Kingdom Childrens Cancer and Leukaemia Groupy.
Further information about this study may be obtained from the paediatric oncology centre treating your patient or from one of the study coordinators :

Professor Vaskar Saha : vaskar.saha@cancer.org.uk

Dr Phil Darbyshire : phil.darbyshire@bhamchildrens.wmids.nhs.uk

NOT FOR CLINICAL USE

Centre Number:
Study Number:
Patient Identification Number for this trial:

PARENT/CHILD CONSENT FORM

Version 4, Dated August 2007

Title of Project: ALLR3, A Protocol for Relapsed and Refractory Acute Lymphoblastic Leukaemia of Childhood

Name of Researcher:

Name of Patient:

Date of Birth:

Please Note. Consent is considered to have been given only if the boxes have been initialled.

	Please Initial Box
I confirm that I have read and understand the information sheet(s) version….. Dated August 2007 for ALLR3 and have had the opportunity to ask questions.	☐
I understand that my/my child's participation is voluntary and I am / he/she is free to withdraw at any time, without giving any reason, without my/his/her medical care or legal rights being affected.	☐
I understand that sections of my/my child's medical notes may be looked at by responsible individuals from the United Kingdom Childrens Cancer and Leukaemia Group or regulatory authorities, where it is relevant to my/my child's taking part in research. I give permission for these individuals to have access to my/my child's records.	☐
I agree/do not agree to my/my child's GP being informed about my registration and subsequent treatment on the ALLR3 trial.	☐
I agree/do not agree for data collected about me/my child to be stored on an electronic database.	☐
I agree/do not agree for additional tissue to be collected, stored and analysed for me/my child for use on ALLR3 study and used for future ethicall approved studies.	☐
I accept/do not accept for my child the randomisations that been offered in this trial.	☐
I agree/ do not agree for my child to take part in the above study.	☐

________________	________________	______________________
Name of patient	Date	Signature
________________	________________	______________________
Name of parent/guardian	Date	Signature
________________	________________	______________________
Name of person taking consent (if different from researcher)	Date	Signature
________________	________________	______________________
Researcher	Date	Signature

Appendix 17: Common Toxicity Criteria

GRADES

Criteria	1 (mild)	2 (moderate)	3 (severe)	4 (Very severe)	5
**Blood/Bone Marrow**					
WBC / x10^9/L	<LLN - 3.0	2.0 - 2.9	1.0 - 1.9	<1.0	Death
ANC/ x10^9/L	< LLN - 1.5	< 1.5-1.0	< 1.0 - 0.5	<0.5	Death
LYMPHS/x10^9/L	<0.8	0.8 - 0.5	0.5 - 0.2	<0.2	Death
PLT/x10^9/L	< LLN - 75.0	< 75.0 - 50.0	< 50.0 - 25.0	< 25.0	Death
HGB g/dl	<LLN - 10.0	< 10.0 -8.0	< 8.0 - 6.5	< 6.5	Death
MARROW CELLULARITY	mildly hypo or ≤ 25% reduction from normal for age	mod.hypo or > 25% - ≤ 50% reduction from normal for age	severe;y hypo or > 50 -≤ 75 % from normal for age		Death
**Hemorrage/ Bleeding**					
HEMORRHAGE	mild without transfusion		Transfusion indicated	Catastrophic bleeding, requiring major non-elective intervention	Death
THROMBOSIS		DVT, no anticoag required	DVT, anticoag required	embolic event inc Pulm Embolism	Death
**Coagulation**					
FIBRINOGEN	<1.0 - 0.75 x LLN or < 25% decrease	<0.75 - 0.5 x LLN or 25-<50% decrease	< 0.5 - 0.25 x LLN or 50 - < 75% decrease	< 0.25 x LLN or 75% decrease	Death
INR	≥ 1 - 1.5 x N	> 1.5 - 2 x N	> 2 x N		
PTT	> 1 - 1.5 x ULN	> 1.5 - 2 x ULN	> 2 x ULN		
**Infection**					
INFECTION*	mild	moderate	severe	life threatening	Death
**Allergy**					
ALLERGIC REACTION	Transient flushing or rash, drug fever < 38C	Rash; flushing; urticaria; dyspnea; drug fever ≥ 38C	sympotatic bronchospasm with or without urticaria;allergy related oedema; hypotension	anaphylaxis	Death
**Constitutional Symptoms**					
FEVER	38.0-39.0C	> 39.0 -40.0C	>40C for ≤ 24hrs	> 40.0 C for > 24 hrs	Death
WEIGHT CHANGE	5 to < 10% from baseline; intervention not indicated	10 - < 20% from baseline; nutritional support indicated	≥20.0% of baseline		
ALOPECIA	Thinning	Complete			
**Dermatology/ Skin**					
RASH/ DESQUAMATION	Macular or papular eruption or erythema	macular/ papular eruption or erythema with pruritis; localised desquamation or other lesions < 50% body	severe, generalized erythrodema or macular, papular eruption; desquamation ≥ 50% body	Generalised exfoliative, ulcerative or bullous dermatitis	Death
INJECTION SITE REACTION / EXTRAVASATION CHANGES	pain; itching; erythema	pain or swelling, with inflammation or phlebitis	Ulceration or necrosis that is severe; operative intervention indicated		Death
**Ocular / Visual**					
CONJUNCTIVITIS	Asymptomatic or minimally symptomatic but not interfering with function	Symptomatic and interfering with function but not ADL**; topical antibiotics or other topical intervention indicated	Symptomatic, interfering with ADL**; operative intervention indictaed		
CORNEAL INFLAMMATION / ULCERATION	abnormal ophthalmic changes only; intervention not indicated	symptomatic, interfering with function but not ADL**	symptomatic and interfering with ADL**	Perforation or blindness (20/200 or worse)	Death
VISION	Symptomatic not interfering with function	Symptomatic interfering with function but not with ADL**	Symtoms interfering with ADL**	blindness	
**Gastrointestinal**					
STOMATITIS	Minimal symptoms, normal diet	Symptomatic but can eat and swallow modified diet	Symptomatoc and unable to adequately aliment or hydrate orally	Symptoms associated with life threateniong consequences	Death
NAUSEA	Loss of appetite without alteration in eating habits	Decreased intake, no sig weight loss, dehydration or malnutrition; IV fluids indicated < 24 hrs	Inadequate oral intake; IV fluids, tube feeding or TPN indicated ≥ 24 hrs	Life-threatening consequences	Death
VOMITING	1 episode in 24 hrs	2-5 episodes in 24 hours; IV fluids indicated	≥ 6 episodes in 24 hrs; IV fluids or TPN indicated ≥24 hrs	Life-threatening consequences	Death
DIARRH0EA	increase of < 4 stools/day	Increase of 4-6 stools per day; IV fluids indicated < 24 hrs; not interfering with ADL**	Increase of ≥7 stools/day; IV fluids ≥ 24 hrs; hospitalisation; interfering with ADL**	Life threatening consequences (e.g. hemodynamic collapse)	Death
CONSTIPATION	Occasional symptoms; occasional stool softener or diet mod or enema	Persistant symptoms with regular use of laxatives or enemas indicated	Symtoms interfering with ADL**; obstipation with manual evacuation indicated	Life threatening consequences (e.g. obstruction, toxic megacolon)	Death
**Hepatic / Pancreas**					
PANCREATITIS	Asmptomatic, enzyem elevartion and /or radiographic findings	symptomatic, medical intervention indicated	Interventional radiology or operative intervention indicated	Life threatening consequences (e.g. circulatory failure, hemorrhage, sepsis)	Death
PANCREAS (imaging)	somnolency	size increased <2x	size increased > 2	hemorrhage- pseudocyst	
LIVER-CLINICAL		Jaundice	Asterixis	encephalopathy or hepatic coma	Death
**Metabolic / Laboratory**					
BILIRUBIN	> ULN - 1.5 x ULN	>1.5 - 3.0 x ULN	>3.0- 10.0 x ULN	>10.0 x ULN	Death
AST, SGOT	> ULN - 2.5 x ULN	> 2.5 - 5.0 x ULN	> 5.0 - 20.0 x ULN	> 20.0 x ULN	Death
ALK PHOSPHATE	> ULN - 2.5 x ULN	> 2.5 - 5.0 x ULN	> 5.0 - 20.0 x ULN	> 20.0 x ULN	Death
ALBUMIN (hypoalbuminemia)	< LLN - 3 g/dl	<3 - 2 g/dl	< 2 g/dl		Death
HAEMATURIA	micro only	intermittent, gross, no clots	persistent, gross or clots may req transf	necrosis or deep bladder ulceration	Death
PROTEINURIA	1 + or 0.15 -1.0 g/24 hours	2+ to 3+ or 1.1 - 3.5 g/24 hrs	4+ or> 3.5g/24 hrs	nephrotic syndrome	Death
CREATININE	> ULN - 1.5 x ULN	> 1.5 - 3.0 x ULN	> 3.0 - 6.0 x ULN	> 6.0 x ULN	Death

Criteria	1 (mild)	2 (moderate)	3 (severe)	4 (Very severe)	5
**Metabolic / Laboratory Cont.**					
HYPERCALCAEMIA	> ULN - 2.9.mmol/L	> 2.9 - 3.1 mmol/L	> 3.1 - 3.4 mmol/L		Death
HYPOCALCAEMIA	> LLN - 2.0.mmol/L	> 2.0 - 1.75 mmol/L	< 1.75 - 1.5 mmol/L	< 1.5 mmol/L	Death
HYPERMAGNESEMIA	> ULN - 1.23 mmol/L		> 1.23- 3.30 mmol/L	> 3.30 mmol/L	Death
HYPOMAGNESEMIA	< LLN - 0.5 mmol/L	< 0.5 - 0.4 mmol /L	< 0.4 - 0.3 mmol / L	<0.3 mmol/L	Death
HYPERKALEMIA	> ULN - 5.5 mmol/L	> 5.5 - 6.0 mmol/L	> 6.0 - 7.0 mmol/L	>7.0 mmol/L	Death
HYPOKALEMIA	<LLN - 3mmol/L		< 3.0 - 2.5 mmol/L	< 2.5 mmol/L	Death
HYPERNATREMIA	> ULN - 150 mmol/L	>150 -155 mmol/L	>155 -160 mmol/L	>160 mmol/L	Death
HYPONATREMIA	< LLN - 130 mmol/L		< 130 - 120 mmol / L	<120 mmol/L	Death
HYPERGLYCEMIA	> ULN - 8.9 mmol/L	> 8.9 - 13.9	>13.9 - 27.8 mmol/L	> 27.8 mmol or acidosis	Death
HYPOGLYCEMIA	< LLN - 3.0.mmol/L	< 3.0 - 2.2. mmol/L	< 2.2 - 1.7 mmol / L	< 1.7 mmol/L	Death
AMYLASE	> ULN - 1.5 x ULN	1.5-2.0 x ULN	2.1-5.0 x ULN	>5.0 x ULN	Death
**Cardiac**					
CARD.RHYTHM	asympt./transient	recur/persist but does not require treatment	symptomatic and requires treatment	Life threatening (e.g. hypotension, syncope)	Death
LEFT VENTRICULAR DIASTOLIC DYSFUNCTION	Asymptomatic, therapy not indicated	Asymptomatic, therapy indicated	Symptomatic CHF responsive to intervention	Refractory CHF, poorly controlled;; intervention such as ventricular assist deveice indicated	Death
LEFT VENTRICULAR SYSTOLIC DYSFUNCTION	Asymptomatic, retsing ejection (EF) <60 - 50%; shortening fraction(SF) < 30 - 24%	Asymptomatic, resting EF < 50 - 40 %, SF < 24 - 15%	Symptomatic CHF responsive to intervention; EF < 40 - 20% SF < 15%	Refractory CHF or poorly controlled; EF < 20%; intervention such as ventricular assist device, ventricular reduction surgery indicated	Death
PERICARDIAL EFFUSION	Asymptomatic effusion		Effusion with physiologic consequences	Life threatening consequences (e.g. tamponade); emergency intervention indicated	Death
HYPERTENSION	Asymptomatic, transient (<24hrs) BP increase > ULN; intervention not indicated	Recurrent or persistant (>24hrs) BP > ULN; monotherapy maybe indicated	Requiring more than one drug or more intensive therapy than previously	Life threatening consequences (e.g. hypertensive crisis)	Death
HYPOTENSION	changes, intervention not indicated	Brief (<24hrs) fluid replacement or other therapy; no physiologic consequences	Sustained ($\geq$ 24hrs) therapy, resolves wihtout persisting physiologic consequences	Shock (e.g. acidemia; impairment of vital organ function)	Death
**Respiratory**					
DYSPNEA	abn PFTs/asymp/ dyspnea on exertion	dyspnea on sig exert.	dyspnea at N activ.	dyspnoea at rest; intubation / ventilator indicated	Death
VITAL CAP.	90-75% of predicted value	<75 - 50% of predicted value	<50 - 25% of predicted value	< 25% of predicted value	Death
PNEUMONITIS/PULMONARY INFILTRATES	Asymptomatic	Sympotomatic, not interfering with ADL**	Symptomatic , interfering with ADL**; Oxygen req	Life threatening; ventilatory support indicated	Death
HYPOXIA		Decreased O2 saturation with exercise (pulse oximetry <88%) or intermittent oxygen required	Decreased O2 saturation at rest;continuous oxygen required	Life threatening; ventilatory support indicated	Death
**Neurology**					
MOOD ALTERATION	Mild mood alteration not interfering with function	Moderate mood alterationinterfering with functio, but not interfering with ADL**; medication indicated	Severe mood alteration interfering with ADL**	Suicidal ideation; danger to self or others	Death
SOMNOLENCE/ DEPRESSED LEVEL OF CONSCIOUSNESS		Somnolence or sedation interfering with function, but not interfering with ADL**	Obtundation or stupor; difficult to arouse; interfering with ADL**	Coma	Death
NEUROPATHY - SENSORY	Asymptomatic;loss of deep tendon reflexes or pasthesia but not interfering with function	Sensory alteration or paresthesia, interfering with function, but not interfering with ADL**	Sensory alteration or paresthesia interfering with ADL**	disabling	Death
NEUROPATHY - MOTOR	Asyptomatic, weakness on exam / testing only	Symptomatic weakness interfering with function, but not interefering with ADL**	Weakness interfering with ADL**; bracing or assistance to walk indicated (e.g.stick)	Life threatening; disabling (e.g.paralysis)	Death
**Pain**					
HEADACHE	mild pain not interfering with function	Moderate pain: pain or analgesics interfering with function, but not interfering with ADL**	Severe pain; pain or analgesics severely interfering with ADL**	disabling	Death
**Auditory**					
HEARING - subj		Hearing loss not req hearing aid or intervention (I.e not interfering with ADL**)	Hearing loss requiring hearing aid or intervention (I.e. interfering with ADL**)	Profound bilateral hearing loss (>90dB)	
HEARING - obj	Threshold shift or loss of 15 - 25 dB relative to baseline; or subjective change in the absence of a Grade 1 threshold shift	Threshold shift or loss of > 25 - 90 dB	Hearing loss suffecient to indictae therapeutic intervention, including hearing aids (e.g. $\geq$ 20dB bilateral HL in the speech frequencies; $\geq$ 30 dB unilateral HL; and requiring additional speech - language related services)	Audiologic indictaion for cochlear implant and requiring additional speech - language related services	
**Performance**					
PERFORMANCE (Lansky Score)	mild restriction (70<90)	ambulatory up 50% (50<70)	bed or wheelchair > 50 % waking hrs (30<50)	no self care (< 30)	

** Activities of daily living

* Use clinical judgement. For example, Grade I would include a line infection, or fever settling within 24-48 hrs of antibiotics in an otherwise well child;
Grade II would be fever settling within 96 hrs; Grade III, taking longer than 96 hrs, or Grade II criteria and an unwell child;

Death is graded as Grade V

Appendix 18: Declaration of Helsinki

Policy

WORLD MEDICAL ASSOCIATION DECLARATION OF HELSINKI

Ethical Principles for Medical Research Involving Human Subjects

Adopted by the 18th WMA General Assembly, Helsinki, Finland, June 1964, and amended by the
29th WMA General Assembly, Tokyo, Japan, October 1975
35th WMA General Assembly, Venice, Italy, October 1983
41st WMA General Assembly, Hong Kong, September 1989
48th WMA General Assembly, Somerset West, Republic of South Africa, October 1996 and the
52nd WMA General Assembly, Edinburgh, Scotland, October 2000

Note of Clarification on Paragraph 29 added by the WMA General Assembly, Washington 2002
Note of Clarification on Paragraph 30 added by the WMA General Assembly, Tokyo 2004

A. INTRODUCTION

1. The World Medical Association has developed the Declaration of Helsinki as a statement of ethical principles to provide guidance to physicians and other participants in medical research involving human subjects. Medical research involving human subjects includes research on identifiable human material or identifiable data.

2. It is the duty of the physician to promote and safeguard the health of the people. The physician's knowledge and conscience are dedicated to the fulfillment of this duty.

3. The Declaration of Geneva of the World Medical Association binds the physician with the words, "The health of my patient will be my first consideration," and the International Code of Medical Ethics declares that, "A physician shall act only in the patient's interest when providing medical care which might have the effect of weakening the physical and mental condition of the patient."

4. Medical progress is based on research which ultimately must rest in part on experimentation involving human subjects.

5. In medical research on human subjects, considerations related to the well-being of the human subject should take precedence over the interests of science and society.

6. The primary purpose of medical research involving human subjects is to improve prophylactic, diagnostic and therapeutic procedures and the understanding of the aetiology and pathogenesis of disease. Even the best proven prophylactic, diagnostic, and therapeutic methods must continuously be challenged through research for their effectiveness, efficiency, accessibility and quality.

7. In current medical practice and in medical research, most prophylactic, diagnostic and therapeutic procedures involve risks and burdens.

NOT FOR CLINICAL USE

8. Medical research is subject to ethical standards that promote respect for all human beings and protect their health and rights. Some research populations are vulnerable and need special protection. The particular needs of the economically and medically disadvantaged must be recognized. Special attention is also required for those who cannot give or refuse consent for themselves, for those who may be subject to giving consent under duress, for those who will not benefit personally from the research and for those for whom the research is combined with care.

9. Research Investigators should be aware of the ethical, legal and regulatory requirements for research on human subjects in their own countries as well as applicable international requirements. No national ethical, legal or regulatory requirement should be allowed to reduce or eliminate any of the protections for human subjects set forth in this Declaration.

B. BASIC PRINCIPLES FOR ALL MEDICAL RESEARCH

10. It is the duty of the physician in medical research to protect the life, health, privacy, and dignity of the human subject.

11. Medical research involving human subjects must conform to generally accepted scientific principles, be based on a thorough knowledge of the scientific literature, other relevant sources of information, and on adequate laboratory and, where appropriate, animal experimentation.

12. Appropriate caution must be exercised in the conduct of research which may affect the environment, and the welfare of animals used for research must be respected.

13. The design and performance of each experimental procedure involving human subjects should be clearly formulated in an experimental protocol. This protocol should be submitted for consideration, comment, guidance, and where appropriate, approval to a specially appointed ethical review committee, which must be independent of the investigator, the sponsor or any other kind of undue influence. This independent committee should be in conformity with the laws and regulations of the country in which the research experiment is performed.
The committee has the right to monitor ongoing trials. The researcher has the obligation to provide monitoring information to the committee, especially any serious adverse events. The researcher should also submit to the committee, for review, information regarding funding, sponsors, institutional affiliations, other potential conflicts of interest and incentives for subjects.

14. The research protocol should always contain a statement of the ethical considerations involved and should indicate that there is compliance with the principles enunciated in this Declaration.

15. Medical research involving human subjects should be conducted only by scientifically qualified persons and under the supervision of a clinically competent medical person. The responsibility for the human subject must always rest with a medically qualified person and never rest on the subject of the research, even though the subject has given consent.

NOT FOR CLINICAL USE

16. Every medical research project involving human subjects should be preceded by careful assessment of predictable risks and burdens in comparison with foreseeable benefits to the subject or to others. This does not preclude the participation of healthy volunteers in medical research. The design of all studies should be publicly available.

17. Physicians should abstain from engaging in research projects involving human subjects unless they are confident that the risks involved have been adequately assessed and can be satisfactorily managed. Physicians should cease any investigation if the risks are found to outweigh the potential benefits or if there is conclusive proof of positive and beneficial results.

18. Medical research involving human subjects should only be conducted if the importance of the objective outweighs the inherent risks and burdens to the subject. This is especially important when the human subjects are healthy volunteers.

19. Medical research is only justified if there is a reasonable likelihood that the populations in which the research is carried out stand to benefit from the results of the research.

20. The subjects must be volunteers and informed participants in the research project.

21. The right of research subjects to safeguard their integrity must always be respected. Every precaution should be taken to respect the privacy of the subject, the confidentiality of the patient's information and to minimize the impact of the study on the subject's physical and mental integrity and on the personality of the subject.

22. In any research on human beings, each potential subject must be adequately informed of the aims, methods, sources of funding, any possible conflicts of interest, institutional affiliations of the researcher, the anticipated benefits and potential risks of the study and the discomfort it may entail. The subject should be informed of the right to abstain from participation in the study or to withdraw consent to participate at any time without reprisal. After ensuring that the subject has understood the information, the physician should then obtain the subject's freely-given informed consent, preferably in writing. If the consent cannot be obtained in writing, the non-written consent must be formally documented and witnessed.

23. When obtaining informed consent for the research project the physician should be particularly cautious if the subject is in a dependent relationship with the physician or may consent under duress. In that case the informed consent should be obtained by a well-informed physician who is not engaged in the investigation and who is completely independent of this relationship.

24. For a research subject who is legally incompetent, physically or mentally incapable of giving consent or is a legally incompetent minor, the investigator must obtain informed consent from the legally authorized representative in accordance with applicable law. These groups should not be included in research unless the research is necessary to promote the health of the population represented and this research cannot instead be performed on legally competent persons.

NOT FOR CLINICAL USE

25. When a subject deemed legally incompetent, such as a minor child, is able to give assent to decisions about participation in research, the investigator must obtain that assent in addition to the consent of the legally authorized representative.

26. Research on individuals from whom it is not possible to obtain consent, including proxy or advance consent, should be done only if the physical/mental condition that prevents obtaining informed consent is a necessary characteristic of the research population. The specific reasons for involving research subjects with a condition that renders them unable to give informed consent should be stated in the experimental protocol for consideration and approval of the review committee. The protocol should state that consent to remain in the research should be obtained as soon as possible from the individual or a legally authorized surrogate.

27. Both authors and publishers have ethical obligations. In publication of the results of research, the investigators are obliged to preserve the accuracy of the results. Negative as well as positive results should be published or otherwise publicly available. Sources of funding, institutional affiliations and any possible conflicts of interest should be declared in the publication. Reports of experimentation not in accordance with the principles laid down in this Declaration should not be accepted for publication.

C. ADDITIONAL PRINCIPLES FOR MEDICAL RESEARCH COMBINED WITH MEDICAL CARE

28. The physician may combine medical research with medical care, only to the extent that the research is justified by its potential prophylactic, diagnostic or therapeutic value. When medical research is combined with medical care, additional standards apply to protect the patients who are research subjects.

29. The benefits, risks, burdens and effectiveness of a new method should be tested against those of the best current prophylactic, diagnostic, and therapeutic methods. This does not exclude the use of placebo, or no treatment, in studies where no proven prophylactic, diagnostic or therapeutic method exists.

30. At the conclusion of the study, every patient entered into the study should be assured of access to the best proven prophylactic, diagnostic and therapeutic methods identified by the study.

31. The physician should fully inform the patient which aspects of the care are related to the research. The refusal of a patient to participate in a study must never interfere with the patient-physician relationship.

32. In the treatment of a patient, where proven prophylactic, diagnostic and therapeutic methods do not exist or have been ineffective, the physician, with informed consent from the patient, must be free to use unproven or new prophylactic, diagnostic and therapeutic measures, if in the physician's judgement it offers hope of saving life, re-establishing health or alleviating suffering. Where possible, these measures should be made the object of research, designed to evaluate their safety and efficacy. In all cases, new information should be recorded and, where appropriate, published. The other relevant

NOT FOR CLINICAL USE

guidelines of this Declaration should be followed.

1 **Note of clarification on paragraph 29 of the WMA Declaration of Helsinki**
The WMA hereby reaffirms its position that extreme care must be taken in making use of a placebo-controlled trial and that in general this methodology should only be used in the absence of existing proven therapy. However, a placebo-controlled trial may be ethically acceptable, even if proven therapy is available, under the following circumstances:
- Where for compelling and scientifically sound methodological reasons its use is necessary to determine the efficacy or safety of a prophylactic, diagnostic or therapeutic method; or
- Where a prophylactic, diagnostic or therapeutic method is being investigated for a minor condition and the patients who receive placebo will not be subject to any additional risk of serious or irreversible harm.

All other provisions of the Declaration of Helsinki must be adhered to, especially the need for appropriate ethical and scientific review.

2 **Note of clarification on paragraph 30 of the WMA Declaration of Helsinki**
The WMA hereby reaffirms its position that it is necessary during the study planning process to identify post-trial access by study participants to prophylactic, diagnostic and therapeutic procedures identified as beneficial in the study or access to other appropriate care. Post-trial access arrangements or other care must be described in the study protocol so the ethical review committee may consider such arrangements during its review.

9.10.2004

NOT FOR CLINICAL USE
